# The unique reactivity of 5,6-unsubstituted 1,4-dihydropyridine in the Huisgen 1,4-diploar cycloaddition and formal [2 + 2] cycloaddition

**DOI:** 10.3762/bjoc.19.73

**Published:** 2023-06-29

**Authors:** Xiu-Yu Chen, Hui Zheng, Ying Han, Jing Sun, Chao-Guo Yan

**Affiliations:** 1 College of Chemistry & Chemical Engineering, Yangzhou University, Jiangsu, Yangzhou 225002, China

**Keywords:** 1,4-dihydropyridine, electron-withdrawing alkyne, formal [2 + 2] cycloaddition, Huisgen's 1,4-dipole, isoquinoline, isoquinolino[1,2-*f*][1,6]naphthyridine

## Abstract

The three-component reaction of isoquinolines, dialkyl acetylenedicarboxylates, and 5,6-unsubstituted 1,4-dihydropyridines in acetonitrile at room temperature afforded functionalized isoquinolino[1,2-*f*][1,6]naphthyridines in good yields and with high diastereoselectivity. More importantly, the formal [2 + 2] cycloaddition reaction of dialkyl acetylenedicarboxylates and 5,6-unsubstituted 1,4-dihydropyridines in refluxing acetonitrile gave unique 2-azabicyclo[4.2.0]octa-3,7-dienes as major products and 1,3a,4,6a-tetrahydrocyclopenta[*b*]pyrroles as minor products via further rearrangement.

## Introduction

Among various well-known cycloaddition reactions such as the 1,3-dipolar cycloaddition reaction, Diels–Alder reaction, and the Povarov reaction, the cycloaddition reaction of Huisgen 1,4-dipoles with activated alkenes received increasing attention [1–3]. The well-known Huisgen 1,4-dipoles have a special kind of zwitterionic intermediates and are usually prepared by a nucleophilic addition of pyridine, quinoline, isoquinoline and other aza-arenes to electron-deficient alkynes [4–8]. The reactive Huisgen 1,4-dipoles have been widely employed as one of the most valuable synthons to construct diverse carbocyclic and heterocyclic systems as well as many open-chain compounds [9–15]. In recent years, in situ generated Huisgen 1,4-dipoles were also widely employed to design highly efficient multicomponent and domino reactions [16–25]. Recently, much attention has been devoted to the development of new domino reactions containing reactive Huisgen 1,4-dipoles as key components for the assembly of many biologically important nitrogen-containing six-membered heterocyclic compounds [26–30].

**Scheme 1:**
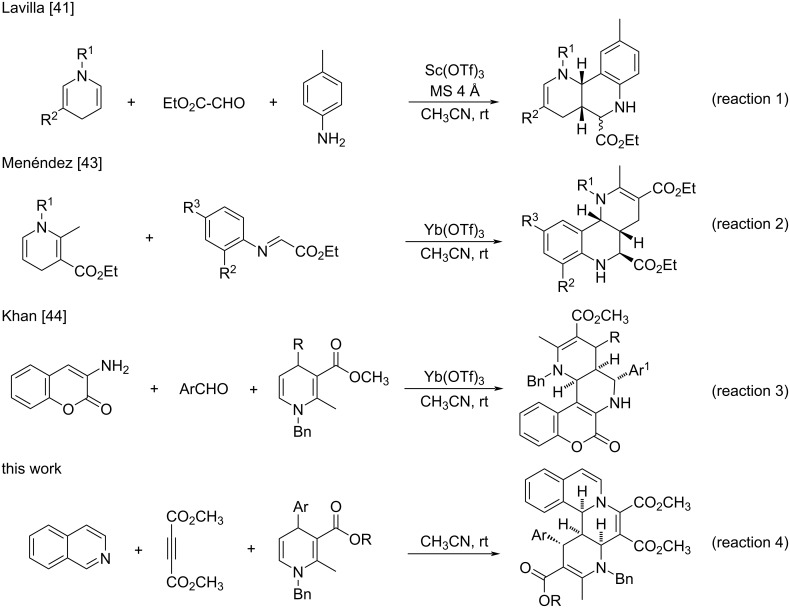
Various cycloaddition reactions of 5,6-unsymmetric 1,4-dihydropyridines.

The 5,6-unsubstituted 1,4-dihydropyridines can be easily prepared from the three-component reaction of an arylamine, cinnamaldehyde, and methyl acetoacetate [31–34]. The unsubstituted C=C double bond in 5,6-unsubstituted 1,4-dihydropyridines exhibits high reactivity and could act as activated alkene to take part in various cycloaddition reactions [35–40]. For example, Lavilla and co-workers developed a Sc(OTf)_3_-catalyzed three-component reaction of 5,6-unsubstituted 1,4-dihydropyridines, arylamines and ethyl glyoxylate for the preparation of various pyrido-fused tetrahydroquinolines (reaction 1 in Scheme 1) [41–42]. Menéndez and co-workers reported a Yb(OTf)_3_-mediated Povarov reaction of imines and *N*-alkyl-1,4-dihydropyridines for the synthesis of hexahydrobenzo[*h*][1,6]naphthyridines (reaction 2 in Scheme 1) [43]. Khan and co-workers employed a one-pot Povarov reaction of 3-aminocoumarins, aldehydes, and 5,6-unsubstituted 1,4-dihydropyridine derivatives for the construction of exo-hexahydrochromeno[3,4-*h*][1,6]naphthyridine-3-carboxylate derivatives (reaction 3 in Scheme 1) [44]. In these reactions, the 5,6-unsubstituted 1,4-dihydropyridines usually behaved as an activated electron-rich dienophile. Inspired by these elegant synthetic methodologies and in continuation of our aim to develop well-known Huisgen 1,4-dipoles for the construction of diverse nitrogen-containing heterocyclic compounds [45–64], herein, we wish to report the use of 5,6-unsubstituted 1,4-dihydropyridines as electron-deficient alkenes in the Huisgen 1,4-diploar cycloaddition and as electron-rich alkenes in formal [2 + 2] cycloadditions for the efficient synthesis of isoquinolino[1,2-*f*][1,6]naphthyridine and 2-azabicyclo[4.2.0]octa-3,7-diene derivatives.

## Results and Discussion

Initially, the reaction conditions were briefly examined by using isoquinoline (**1**), dimethyl acetylenedicarboxylate (DMAD, **2**) and 5,6-unsubstituted 1,4-dihydropyridine **3** as standard reaction (Table 1). The three-component reaction was carried out in common solvents such as ethanol, methanol, dichloromethane, and chloroform at room temperature for two hours. The expected isoquinolino[1,2-*f*][1,6]naphthyridine derivative **4a** was successfully obtained in 35%, 40% 70% and 65% yields, respectively (Table 1, entries 1–4). The reaction in acetonitrile afforded the product **4a** in 75% yield (Table 1, entry 5). When the reaction time was extended to 12 h at room temperature, the yield of product **4a** did not increase (Table 1, entry 6). When the reaction was carried out in acetonitrile at elevated temperatures, the yield of product **4a** decreased to 68% and 55% (Table 1, entries 7 and 8). Therefore, the optimal reaction conditions for this three-component reaction were simply carrying out the reaction in acetonitrile at room temperature for two hours.

**Table 1:** Optimizing the reaction conditions.^a^

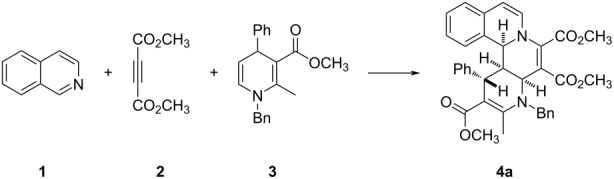				

Entry	Solvent	Temp. [°C]	Time [h]	Yield [%]^b^

1	EtOH	rt	2	35
2	MeOH	rt	2	40
3	CH_2_Cl_2_	rt	2	70
4	CHCl_3_	rt	2	65
**5**	**MeCN**	**rt**	**2**	**75**
6	MeCN	rt	12	72
7	MeCN	50	2	68
8	MeCN	80	2	55

^a^Reaction conditions: isoquinoline (0.5 mmol), DMAD (0.6 mmol), 5,6-unsubstituted 1,4-dihydropyridine (0.5 mmol), solvent (5.0 mL). ^b^Isolated yields.

**Figure 1:**
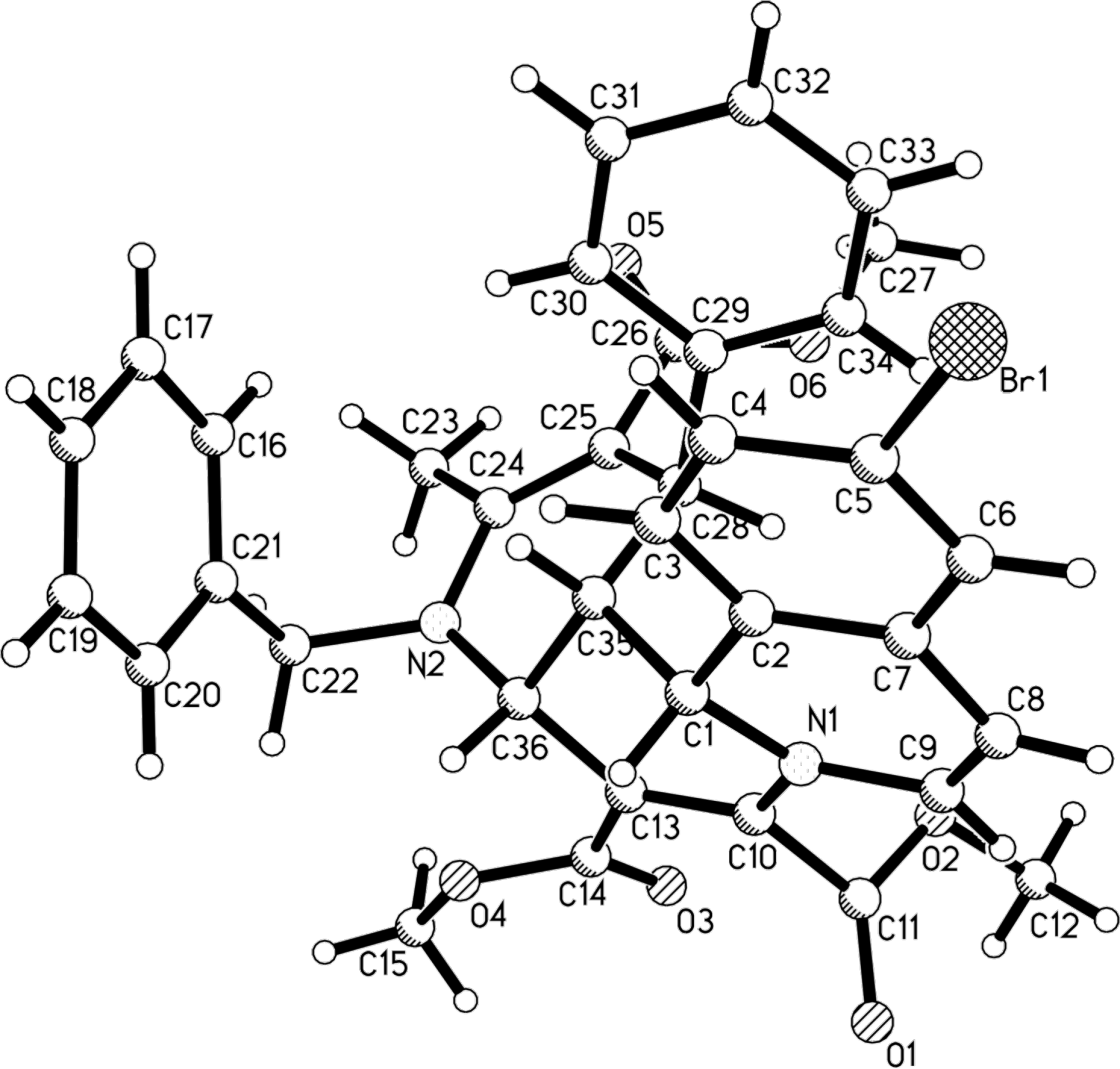
Single crystal structure of the compound **4k**.

Under the optimal reaction conditions, various substrates were employed in the reaction for developing the scope of the reaction and the results are summarized in Table 2. It can be seen that all reactions gave the desired isoquinolino[1,2-*f*][1,6]naphthyridine derivatives **4a–o** in good to excellent yields. Isoquinoline itself and its 4-, 5-, and 6-bromo-substituted derivatives were successfully used in the reaction. Dimethyl or diethyl acetylenedicarboxylates gave the products in comparable yields in the reaction. The 5,6-unsubstituted 1,4-dihydropyridines with an *N*-benzyl group usually gave the products in good yields (Table 2, entries 1–12). It should be pointed out that 6-unsubstituted 1,4-dihydropyridines with an *N*-(3,4-(CH_3_O)_2_C_6_H_3_CH_2_CH_2_) group also afforded the desired product **4h** in 88% yield (Table 2, entry 8). Even 5,6-unsubstituted 1,4-dihydropyridines with an *N-n-*Bu group also gave the products **4m** and **4n** in satisfactory yields. At last, 5,6-unsubstituted 1,4-dihydropyridines derived from the condensation of acetylacetone also afforded the expected product **4o** in 65% yield. The chemical structures of the obtained isoquinoline[2,1-*h*][1,7]naphthyridines **4a–o** were fully characterized by various spectroscopy methods and further confirmed by determination of the single crystal structure of compound **4k** (Figure 1, CCDC 2059918). Though there are four chiral centers in the product structure of the isoquinolino[1,2-*f*][1,6]naphthyridine, the ^1^H NMR spectra of the products all showed that only one diastereomer was produced in the reaction, which showed that this reaction has a high diastereoselectivity. From Figure 1, it can be seen that the three protons and the phenyl group have *cis*-configuration in the hexahydro-1,6-naphthyridyl ring.

**Table 2:** Synthesis of isoquinolino[1,2-*f*][1,6]naphthyridines **4a**–**o**.^a^

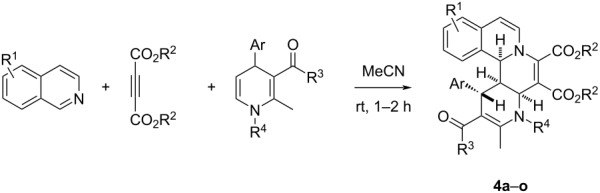							

Entry	Compd	R^1^	R^2^	Ar	R^3^	R^4^	Yield [%]^b^

1	**4a**	H	CH_3_	C_6_H_5_	OCH_3_	Bn	75
2	**4b**	H	CH_3_	C_6_H_5_	OEt	Bn	65
3	**4c**	H	CH_3_	*p*-FC_6_H_4_	OCH_3_	Bn	58
4	**4d**	H	CH_3_	*p-*CH_3_OC_6_H_4_	OCH_3_	Bn	88
5	**4e**	H	C_2_H_5_	C_6_H_5_	OCH_3_	Bn	80
6	**4f**	H	C_2_H_5_	C_6_H_5_	OC_2_H_5_	Bn	82
7	**4g**	H	CH_3_	C_6_H_5_	OCH_3_	*p*-CH_3_OC_6_H_4_CH_2_	84
8	**4h**	H	CH_3_	C_6_H_5_	OCH_3_	3,4-(CH_3_O)_2_C_6_H_3_(CH_2_)_2_	88
9	**4i**	4-Br	CH_3_	C_6_H_5_	OCH_3_	Bn	78
10	**4j**	5-Br	CH_3_	C_6_H_5_	OCH_3_	Bn	67
11	**4k**	6-Br	CH_3_	C_6_H_5_	OCH_3_	Bn	65
12	**4l**	4-Br	CH_3_	C_6_H_5_	OC_2_H_5_	Bn	74
13	**4m**	H	CH_3_	C_6_H_5_	OCH_3_	*n*-Bu	77
14	**4n**	5-Br	CH_3_	C_6_H_5_	OCH_3_	*n*-Bu	78
15	**4o**	H	CH_3_	C_6_H_5_	CH_3_	Bn	65

^a^Reaction conditions: isoquinoline (0.5 mmol), dialkyl acetylenedicarboxylate (0.6 mmol), 5,6-unsubstituted 1,4-dihydropyridine (0.5 mmol), CH_3_CN (5.0 mL), rt, 2 h. ^b^Isolated yields.

**Figure 2:**
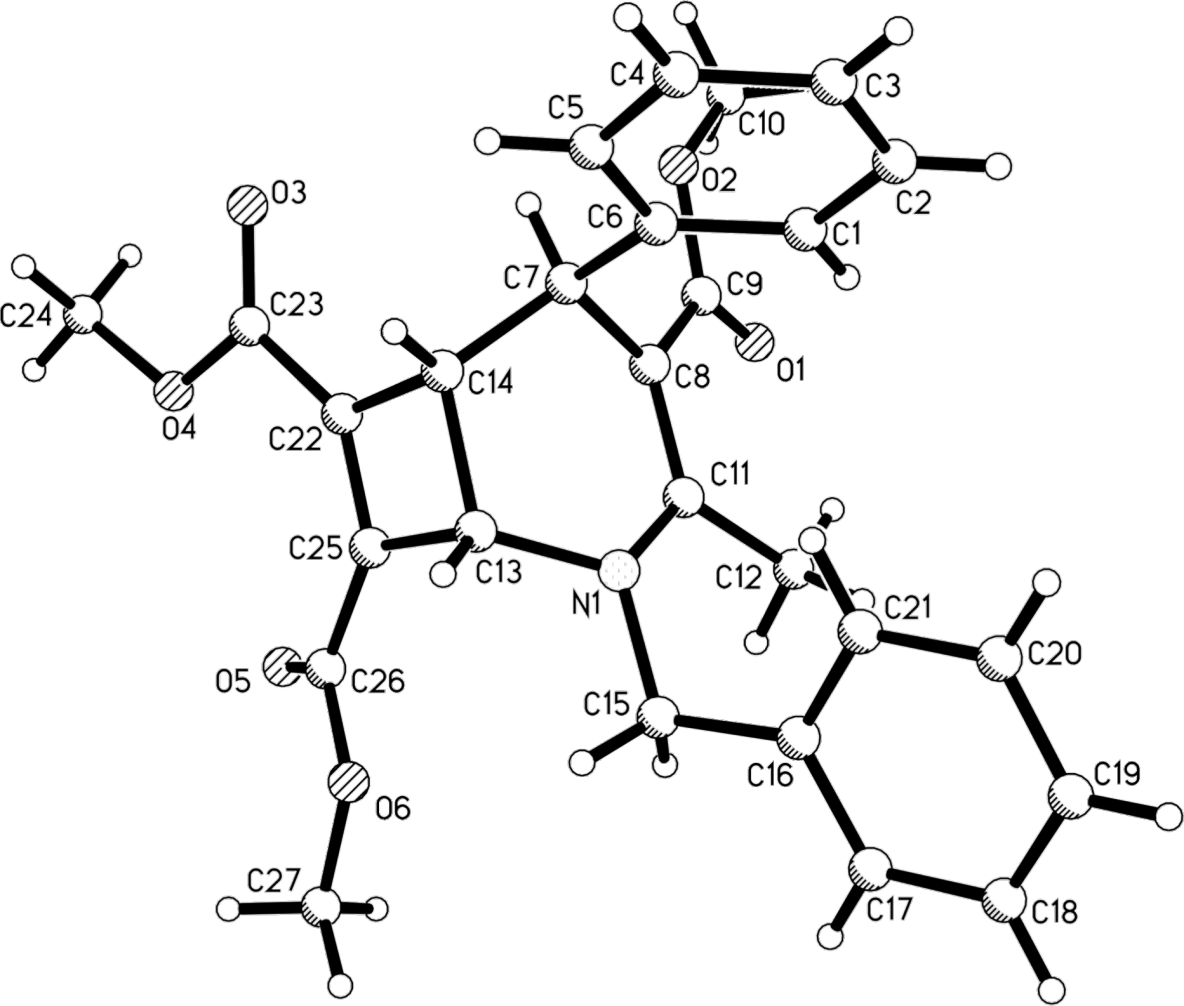
Single crystal structure of compound **5a**.

**Figure 3:**
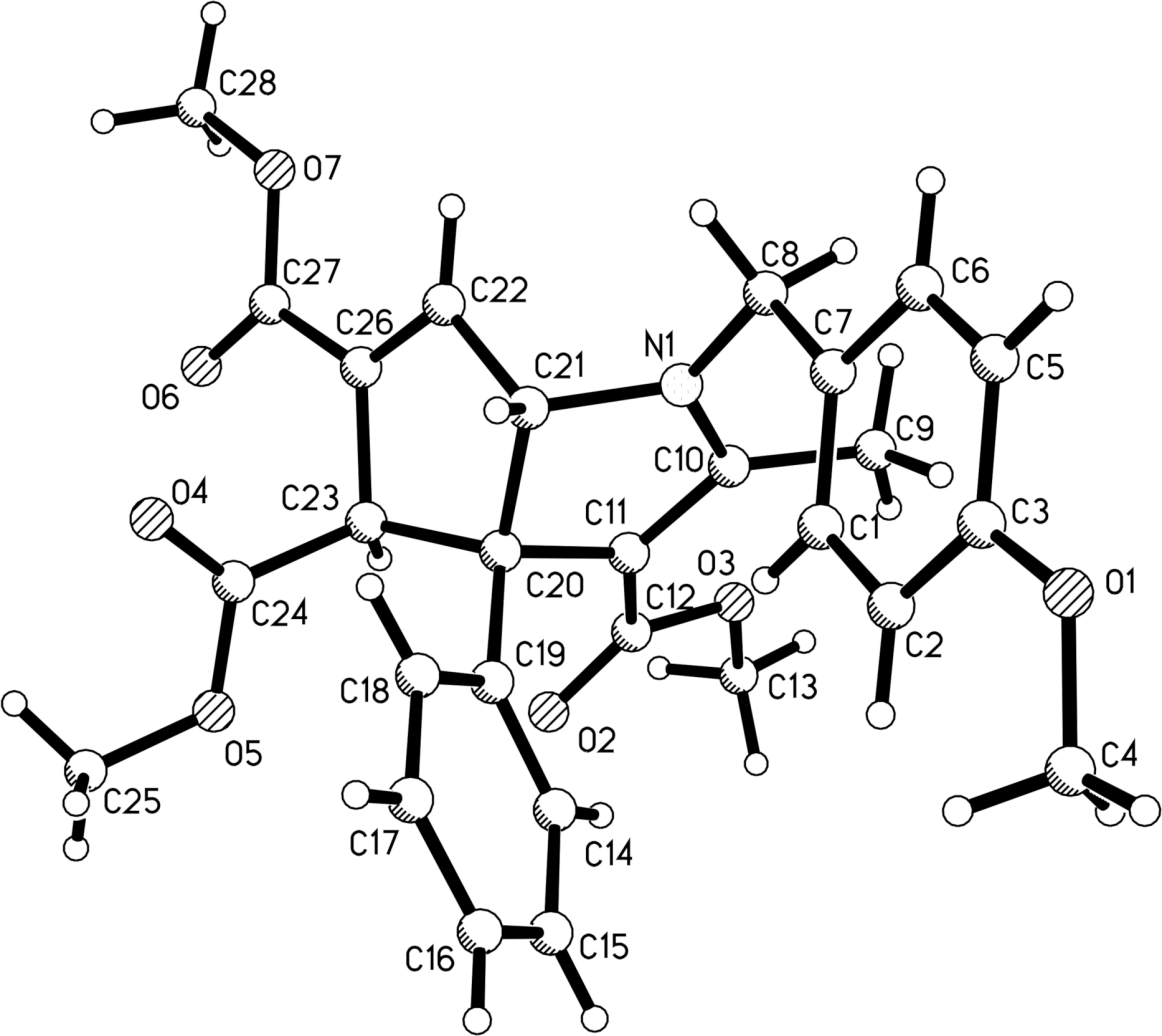
Single crystal structure of compound **6f**.

During the investigation of the above three-component reaction, we found that the three-component reaction of isoquinoline, dimethyl acetylenedicarboxylate and 5,6-unsubstituted 1,4-dihydropyridines with *N*–Ar groups did not give the above isoquinolino[1,2-*f*][1,6]naphthyridines, but the unique 2-azabicyclo[4.2.0]octa-3,7-diene-7,8-dicarboxylates were isolated in moderate yields. These products were obviously produced from the formal [2 + 2] cycloaddition between dimethyl acetylenedicarboxylate and the 5,6-unsubstituted 1,4-dihydropyridine. Therefore, the reactions of dimethyl acetylenedicarboxylate and 5,6-unsubstituted 1,4-dihydropyridines were carefully explored. After adjusting the reaction conditions, we were pleased to find that 2-azabicyclo[4.2.0]octa-3,7-dienes **5a–o** could be successfully obtained in moderate to good yields by carrying out the reaction of dimethyl acetylenedicarboxylate and 5,6-unsubstituted 1,4-dihydropyridines in refluxing acetonitrile for three hours (Table 3). As can be seen, unsymmetric 1,4-dihydropyridines with *N*–Bn and *N*-4-(CH_3_OC_6_H_4_CH_2_) groups can be successfully employed in the reaction (Table 3, entries 1–10). Additionally, 5,6-unsubstituted 1,4-dihydropyridines with various *N*–Ar groups also gave the expected products **5k–o** in good yields. Sometimes, as unexpected byproducts, 1,3a,4,6a-tetrahydrocyclopenta[*b*]pyrrole derivatives **6e**, **6f**, **6i**, **6k**, **6l**, and **6m** were isolated in 23–39% yield from the reaction mixture. In other cases, the corresponding 1,3a,4,6a-tetrahydrocyclopenta[*b*]pyrrole derivatives could not be isolated due to too low yields. By analyzing the chemical structures of the 1,3a,4,6a-tetrahydrocyclopenta[*b*]pyrrole derivatives **6**, it was found that the 1,4-dihydropyridinyl ring of the substrate was converted to a fused pyrrole ring, which might be a result from a rearrangement process of the formed 2-azabicyclo[4.2.0]octa-3,7-diene-7,8-dicarboxylates **5a–o** at elevated temperature. The chemical structures of both bicyclic compounds **5a–o** and **6a–o** were fully characterized by various spectroscopy methods. The single crystal structures of compounds **5a** (Figure 2) and **6f** (Figure 3) were successfully determined by X-ray diffraction analysis. From Figure 2 (compound **5a**), it can be seen that the cyclobutenyl ring and the 1,4-dihydropyridyl ring exist on the fused position. The two protons at the bridged position and the phenyl group are *cis*-configured. From Figure 3 (compound **6f**), it can be seen that the fused pyrrole ring and the cyclopentyl ring are butterfly shaped. The unusual feature is that the C=C double bond is not located between the two carbon atoms substituted with the methoxycarbonyl groups, but between the methylene carbon atom and one carbon atom connected with an electron-withdrawing methoxycarbonyl group. The aryl group and the neighbouring methoxycarbonyl group are *cis-*configured.

**Table 3:** Synthesis of the bicyclic compounds **5a–o** and **6a–o**.^a^

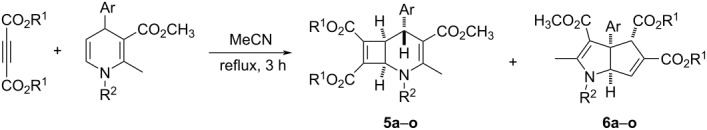							

Entry	R^1^	Ar	R^2^	Compd	Yield (%)^b^	Compd	Yield (%)^b^

1	CH_3_	C_6_H_5_	Bn	**5a**	89%	**6a**	–
2	CH_2_CH_3_	C_6_H_5_	Bn	**5b**	70%	**6b**	–
3	CH_3_	*o*-CH_3_OC_6_H_4_	Bn	**5c**	80%	**6c**	–
4	CH_2_CH_3_	*o*-CH_3_OC_6_H_4_	Bn	**5d**	65%	**6d**	–
5	CH_2_CH_3_	*p*-NO_2_C_6_H_4_	Bn	**5e**	36%	**6e**	35%
6	CH_3_	C_6_H_5_	*p*-CH_3_OC_6_H_4_CH_2_	**5f**	40%	**6f**	23%
7	CH_3_	*o*-CH_3_OC_6_H_4_	*p*-CH_3_OC_6_H_4_CH_2_	**5g**	57%	**6g**	–
8	CH_2_CH_3_	*o*-CH_3_OC_6_H_4_	*p*-CH_3_OC_6_H_4_CH_2_	**5h**	62%	**6h**	–
9	CH_2_CH_3_	*p*-NO_2_C_6_H_4_	*p*-CH_3_OC_6_H_4_CH_2_	**5i**	41%	**6i**	39%
10	CH_3_	*p*-NO_2_C_6_H_4_	*p*-CH_3_OC_6_H_4_CH_2_	**5j**	64%	**6j**	–
11	CH_3_	C_6_H_5_	*p*-CH_3_OC_6_H_4_	**5k**	35%	**6k**	36%
12	CH_2_CH_3_	C_6_H_5_	*p*-CH_3_C_6_H_4_	**5l**	31%	**6l**	33%
13	CH_3_	C_6_H_5_	*p*-BrC_6_H_4_	**5m**	33%	**6m**	35%
14	CH_3_	C_6_H_5_	*m*-ClC_6_H_4_	**5n**	55%	**6n**	–
15	CH_3_	*o*-CH_3_OC_6_H_4_	*o*-CH_3_C_6_H_4_	**5o**	64%	**6o**	–

^a^Reaction conditions: dialkyl acetylenedicarboxylate (0.9 mmol), 5,6-unsubstituted 1,4-dihydropyridine (0.3 mmol), CH_3_CN (5.0 mL), reflux, 3 h. ^b^Isolated yields.

**Scheme 2:**
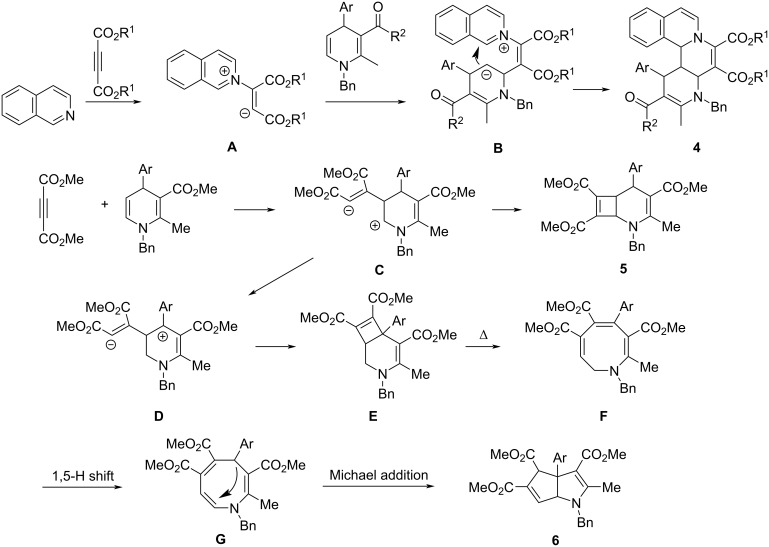
Plausible reaction mechanism for the various products **4**, **5**, and **6**.

For explaining the formation of the various cyclic compounds, a plausible reaction mechanism was proposed on the base of the previously reported works [41–44] and the present experiments (Scheme 2). Initially, the nucleophilic addition of isoquinoline to dimethyl acetylenedicarboxylate gives the well-known Huisgen 1,4-dipole **A**. Secondly, Michael addition of the 1,4-dipole **A** to 5,6-unsubstituted 1,4-dihydropyridine gives the adduct intermediate **B**. At last, the intramolecular coupling of the negative and the positive charges in intermediate **B** directly affords isoquinolino[1,2-*f*][1,6]naphthyridines **4**. Because all reactions in this process are retro-equilibrium reactions, the most thermodynamically stable diastereomer is preferentially produced in the reaction. In the absence of isoquinoline, the 5,6-unsubstituted 1,4-dihydropyridine acts as an active enamine, which adds to dimethyl acetylenedicarboxylate to give the adduct **C**. Then, the direct coupling of the positive charge and the negative charge affords the 2-azabicyclo[4.2.0]octa-3,7-diene **5**. On the other hand, a carbenium ion **D** can be formed by migration of a hydrogen atom in intermediate **C**, which in turn converts into a fused bicyclic intermediate **E** by a charge coupling process. At elevated temperature, the ring-opening of the unstable cyclobutenyl ring gives a 1,2-dihydroazocine intermediate **F**, which is transformed into a 1,4-dihydroazocine intermediate **G** by a 1,5-H shift process. At last, the tetrahydrocyclopenta[*b*]pyrrole **6** is formed by an intramolecular Michael addition process.

## Conclusion

In summary, we have investigated the three-component reaction of isoquinoline, dialkyl acetylenedicarboxylate and 5,6-unsubstituted 1,4-dihydropyridines. This reaction successfully provided an efficient protocol for the synthesis of functionalized isoquinolino[1,2-*f*][1,6]naphthyridines in good yields and with high diastereoselectivity. We also found that the unique 2-azabicyclo[4.2.0]octa-3,7-diene and 1,3a,4,6a-tetrahydrocyclopenta[*b*]pyrrole derivatives can be conveniently produced by the cycloaddition reaction of dialkyl acetylenedicarboxylates and 5,6-unsubstituted 1,4-dihydropyridines. The advantages of the reaction include the use of readily available starting materials, simple reaction conditions, without using any catalyst, high molecular diversity and atomic economy. Therefore, this reaction not only successfully developed unprecedented synthetic reactivity of the electron-deficient alkynes, but also provides efficient synthetic methodologies for complex nitrogen-containing heterocycles. The potential application of this reaction in organic synthesis and medicinal chemistry might be significant.

## Experimental

### General procedure for the three-component reaction of isoquinoline, dialkyl acetylenedicarboxylate and 5,6-unsubstituted 1,4-dihydropyridine

A mixture of isoquinoline (0.5 mmol), dialkyl acetylenedicarboxylate (0.6 mmol), 5,6-unsubstituted 1,4-dihydropyridine (0.5 mmol) in acetonitrile (5.0 mL) was stirred at room temperature for two hours. After removing the solvent by rotatory evaporation at reduced pressure, the residue was subjected to column chromatography with petroleum ether and ethyl acetate (v/v = 5:1) as eluent to give the pure product for analysis.

**Trimethyl 4-benzyl-3-methyl-1-phenyl-4,4a,13b,13c-tetrahydro-1*H*-isoquinolino[1,2-*f*][1,6]naphthyridine-2,5,6-tricarboxylate (4a)**: orange solid, 75%; mp 174–175 °C; ^1^H NMR (400 MHz, CDCl_3_) δ 7.48–7.42 (m, 4H, ArH), 7.39–7.35 (m, 1H, ArH), 6.80 (t, *J* = 7.6 Hz, 1H, ArH), 6.75–6.71 (m, 4H, ArH), 6.63 (d, *J* = 6.4 Hz, 2H, ArH), 6.39 (t, *J* = 7.6 Hz, 1H, ArH), 6.22 (d, *J* = 8.0 Hz, 1H, ArH), 5.91 (d, *J* = 8.0 Hz, 1H, CH), 5.43 (d, *J* = 8.0 Hz, 1H, CH), 5.25 (s, 1H, CH), 5.04 (d, *J* = 15.6 Hz, 1H, CH_2_), 4.60 (d, *J* = 6.0 Hz, 1H, CH), 4.45 (d, *J* = 15.6 Hz, 1H, CH_2_), 4.17 (d, *J* = 8.8 Hz, 1H, CH), 3.93 (s, 3H, OCH_3_), 3.77 (s, 3H, OCH_3_), 3.28 (s, 3H, OCH_3_), 2.60 (t, *J* = 7.6 Hz, 1H, CH), 2.44 (s, 3H, CH_3_) ppm; ^13^C NMR (100 MHz, CDCl_3_) δ 168.7, 166.2, 164.9, 150.2, 146.1, 145.7, 138.7, 129.3, 128.8, 128.1, 128.0, 127.8, 127.5, 127.3, 127.0, 126.3, 126.1, 125.1, 124.7, 124.6, 106.3, 104.6, 102.8, 61.3, 57.6, 56.0, 53.0, 51.6, 50.0, 44.8, 39.5, 17.9 ppm. IR (KBr) ν: 3732, 3023, 2952, 2843, 1967, 1737, 1678, 1556, 1372, 1147, 1097, 1010, 957, 866, 759 cm^−1^; HRESIMS (*m*/*z*): [M + Na]^+^ calcd for C_36_H_34_NaN_2_O_6_, 613.2315; found, 613.2309.

### General procedure for the reaction of dialkyl acetylenedicarboxylate and 5,6-unsubstituted 1,4-dihydropyridine

A mixture of dialkyl acetylenedicarboxylate (0.9 mmol), 5,6-unsubstituted 1,4-dihydropyridine (0.3 mmol) in acetonitrile (5.0 mL) was heated a reflux for three hours. After removing the solvent by rotatory evaporation at reduced pressure, the residue was subjected to column chromatography with petroleum ether and ethyl acetate (v/v = 6:1) as eluent to give the pure product for analysis.

**7,8-Diethyl 4-methyl 2-benzyl-3-methyl-5-(4-nitrophenyl)-2-azabicyclo[4.2.0]octa-3,7-diene-4,7,8-tricarboxylate (5e)**: yellow solid, 36%; mp 161–163 °C; ^1^H NMR (400 MHz, CDCl_3_) δ 8.07–8.05 (m, 2H, ArH), 7.37–7.34 (m, 2H, ArH), 7.33–7.29 (m, 3H, ArH), 7.13–7.10 (m, 2H, ArH) 4.86 (d, *J* = 15.2 Hz, 1H, CH_2_), 4.59 (s, 1H, CH), 4.48 (d, *J* = 15.2 Hz, 1H, CH_2_), 4.37–4.34 (m, 1H, CH), 4.34–4.31 (m, 2H, CH_2_), 4.31–4.26 (m, 2H, CH_2_), 3.71 (d, *J* = 4.4 Hz, 1H, CH), 3.63 (s, 3H, OCH_3_), 2.51 (s, 3H, CH_3_), 1.40–1.37 (m, 3H, CH_3_), 1.37–1.33 (m, 3H, CH_3_) ppm; ^13^C NMR (100 MHz, CDCl_3_) δ 169.3, 160.9, 160.7, 156.2, 152.0, 146.4, 144.5, 137.2, 136.1, 128.7, 128.5, 128.1, 127.9, 123.4, 101.1, 61.5, 61.4, 56.6, 53.8, 51.8, 51.0, 38.9, 17.3, 14.1, 14.1 ppm; IR (KBr) ν: 3746, 2983, 2945, 1729, 1651, 1557, 1434, 1347, 1251, 1129, 1088, 841, 732, 709 cm^−1^; HRESIMS (*m*/*z*): [M + H]^+^) calcd for C_29_H_31_N_2_O_8_, 535.2075; found, 535.2076.

**4,5-Diethyl 3-methyl 1-benzyl-2-methyl-3a-(4-nitrophenyl)-1,3a,4,6a-tetrahydrocyclopenta[*b*]pyrrole-3,4,5-tricarboxylate (6e)**: yellow solid, 35%; mp 153–155 °C; ^1^H NMR (400 MHz, CDCl_3_) δ 7.98 (d, *J* = 8.8 Hz, ArH), 7.45–7.37 (m, 5H, ArH), 7.30–7.27 (m, 2H, ArH), 6.70 (s, 1H, CH_2_), 5.07–5.05 (m, 1H, CH), 5.00–4.98 (m, 1H, CH), 4.76 (d, *J* = 15.2 Hz, 1H, CH_2_), 4.37 (d, *J* = 15.6 Hz, 1H, CH_2_), 4.29–4.21 (m, 1H, CH_2_), 4.21–4.14 (m, 1H, CH_2_), 3.91–3.82 (m, 1H, CH_2_), 3.82–3.72 (m, 1H, CH_2_), 3.69 (s, 3H, OCH_3_), 2.34 (s, 3H, CH_3_), 1.29 (t, *J* = 7.2 Hz, CH_3_), 0.99 (t, *J* = 7.2 Hz, CH_3_) ppm; ^13^C NMR (100 MHz, CDCl_3_) δ 172.2, 166.1, 163.5, 158.0, 149.9, 146.3, 137.8, 137.4, 135. 9, 129.2, 128.5, 128.0, 127.2, 123.0, 101.9, 75.5, 62.9, 61.1, 61.0, 58.3, 50.3, 49.2, 14.1, 13.8, 13.4 ppm; IR (KBr) ν: 3069, 2981, 1736, 1660, 1552, 1514, 1344, 1222, 1121, 1040, 914, 854, 769, 704 cm^−1^; HRESIMS (*m*/*z*): [M + Na]^+^ calcd for C_29_H_30_NaN_2_O_8_, 557.1894; found, 557.1891.

## Supporting Information

The crystallographic data of compounds **4k** (CCDC 2260340), **5a** (CCDC 2260341), and **5f** (CCDC 2260342) have been deposited at the Cambridge Crystallographic Data Center (https://www.ccdc.cam.ac.uk).

File 1 Characterization data and ^1^H NMR, ^13^C NMR, HRMS spectra of the synthesized compounds.
