# Supplementary material for: The unique reactivity of 5,6-unsubstituted 1,4-dihydropyridine in the Huisgen 1,4-diploar cycloaddition and formal [2 + 2] cycloaddition
Source: Beilstein J Org Chem. 2023 Jun 29;19:982–90. doi: 10.3762/bjoc.19.73 (PMC10315888; doi:10.3762/bjoc.19.73)
Supplement: File 1 — Characterization data and 1H NMR, 13C NMR, HRMS spectra of the synthesized compounds. [file Beilstein_J_Org_Chem-19-982-s001.pdf]

BEILSTEIN JOURNAL OF ORGANIC CHEMISTRY

Supporting Information

for

The unique reactivity of 5,6-unsubstituted 1,4-dihydropyridine in the Huisgen 1,4-diploar cycloaddition and formal [2 + 2] cycloaddition

Xiu-Yu Chen, Hui Zheng, Ying Han, Jing Sun and Chao-Guo Yan

Beilstein J. Org. Chem. **2023,** *19,* 982–990. doi:10.3762/bjoc.19.73

Characterization data and ^{1}H NMR, ^{13}C NMR, HRMS spectra of the synthesized compounds

License and Terms: This is a supporting information file under the terms of the Creative Commons Attribution License (https://creativecommons.org/licenses/by/4.0). Please note that the reuse, redistribution and reproduction in particular requires that the author(s) and source are credited and that individual graphics may be subject to special legal provisions.
The license is subject to the *Beilstein Journal of Organic Chemistry* terms and conditions: (https://www.beilstein-journals.org/bjoc/terms)

Trimethyl 4-benzyl-3-methyl-1-phenyl-4,4a,13b,13c-tetrahydro-1*H*-isoquinolino[1,2-*f*][1,6]naphthyridine-2,5,6-tricarboxylate (4a): orange solid, 75%, m.p. 174-175 °C; ^{1}H NMR (400 MHz, $CDCl_3$) δ 7.48-7.42 (m, 4H, ArH), 7.39-7.35 (m, 1H, ArH), 6.80 (t, *J* = 7.6 Hz, 1H, ArH), 6.75-6.71 (m, 4H, ArH), 6.63 (d, *J* = 6.4 Hz, 2H, ArH), 6.39 (t, *J* = 7.6 Hz, 1H, ArH), 6.22 (d, *J* = 8.0 Hz, 1H, ArH), 5.91 (d, *J* = 8.0 Hz, 1H, CH), 5.43 (d, *J* = 8.0 Hz, 1H, CH), 5.25 (s, 1H, CH), 5.04 (d, *J* = 15.6 Hz, 1H, CH_2), 4.60 (d, *J* = 6.0 Hz, 1H, CH), 4.45 (d, *J* = 15.6 Hz, 1H, CH_2), 4.17 (d, *J* = 8.8 Hz, 1H, CH), 3.93 (s, 3H, OCH_3), 3.77 (s, 3H, OCH_3), 3.28 (s, 3H, OCH_3), 2.60 (t, *J* = 7.6 Hz, 1H, CH), 2.44 (s, 3H, CH_3) ppm. ^{13}C NMR (100 MHz, $CDCl_3$) δ 168.7, 166.2, 164.9, 150.2, 146.1, 145.7, 138.7, 129.3, 128.8, 128.1, 128.0, 127.8, 127.5, 127.3, 127.0, 126.3, 126.1, 125.1, 124.7, 124.6, 106.3, 104.6, 102.8, 61.3, 57.6, 56.0, 53.0, 51.6, 50.0, 44.8, 39.5, 17.9 ppm. IR (KBr) υ: 3732, 3023, 2952, 2843, 1967, 1737, 1678, 1556, 1372, 1147, 1097, 1010, 957, 866, 759 cm^{-1}; MS (*m*/*z*): HRMS (ESI) Calcd. for $C_{36}H_{34}NaN_2O_6$ ([M+Na]$^+$): 613.2315, found: 613.2309.

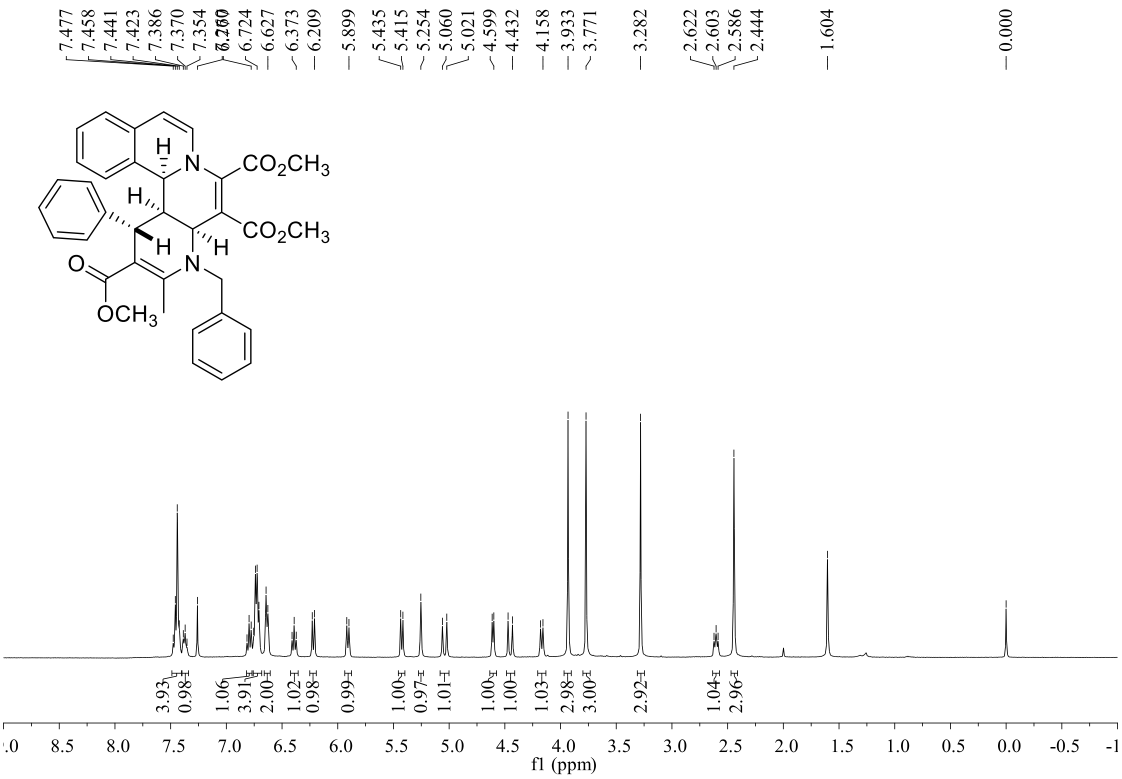

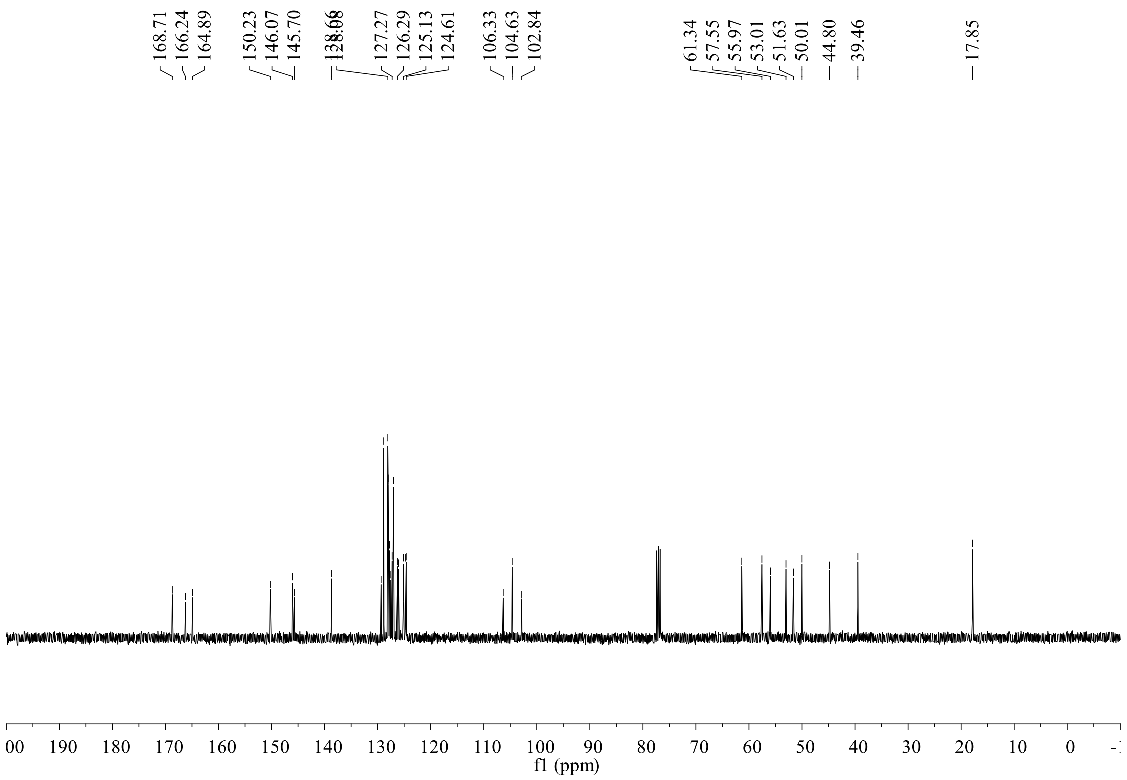
168.71
166.24
164.89
150.23
146.07
145.70
128.66
128.08
127.27
126.29
125.13
124.61
106.33
104.63
102.84
61.34
57.55
55.97
53.01
51.63
50.01
44.80
39.46
17.85
f1 (ppm)

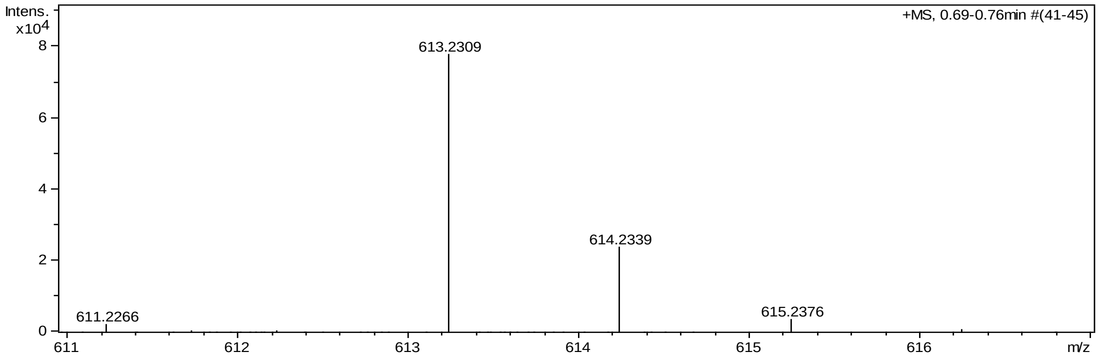
Intens.
x10^4
+MS, 0.69-0.76min #(41-45)
613.2309
614.2339
615.2376
611.2266
m/z

2-Ethyl 5,6-dimethyl 4-benzyl-3-methyl-1-phenyl-4,4a,13b,13c-tetrahydro-1*H*-isoquinolino[1,2-*f*][1,6]naphthyridine-2,5,6-tricarboxylate (4b): orange solid, 65%, m.p. 216-218 °C; ^{1}H NMR (400 MHz, $CDCl_3$) δ 7.48-7.43 (m, 4H, ArH), 7.39-7.35 (m, 1H, ArH), 6.81 (t, *J* = 7.6 Hz, 1H, ArH), 6.77-6.70 (m, 4H, ArH), 6.65 (d, *J* = 7.6 Hz, 2H, ArH), 6.40 (t, *J* = 7.6 Hz, 1H, ArH), 6.23 (d, *J* = 8.0 Hz, 1H, ArH), 5.91 (d, *J* = 8.0 Hz, 1H, CH), 5.43 (d, *J* = 7.6 Hz, 1H, CH), 5.26 (s, 1H, CH), 5.05 (d, *J* = 16.0 Hz, 1H, CH_2), 4.60 (d, *J* = 5.6 Hz, 1H, CH), 4.44 (d, *J* = 15.6 Hz, 1H, CH_2), 4.19 (d, *J* = 8.8 Hz, 1H, CH), 3.94 (s, 3H, OCH_3), 3.79-3.74 (m, 5H, OCH_3, OCH_2), 2.58 (t, *J* = 7.6 Hz, 1H, CH), 2.45 (s, 3H, CH_3), 0.85 (t, *J* = 7.2 Hz, 3H, CH_3) ppm. ^{13}C NMR (100 MHz, $CDCl_3$) δ 168.2, 166.3, 164.9, 150.2, 146.0, 145.7, 138.7, 129.3, 128.8, 128.2, 128.1, 127.7, 127.6, 127.3, 127.0, 126.3, 126.0, 125.1, 124.7, 124.6, 106.3, 104.6, 102.9, 61.4, 58.7, 57.6, 56.0, 53.0, 51.6, 44.7, 39.4, 17.8, 13.9 ppm. IR (KBr) υ: 3765, 3043, 2892, 2823, 1953, 1734, 1634, 1556, 1352, 1217, 1097, 1011, 943, 876, 754 cm^{-1}; MS (*m*/*z*): HRMS (ESI) Calcd. for $C_{37}H_{36}NaN_2O_6$ ([M+Na]$^+$): 627.2471, found: 627.2473.

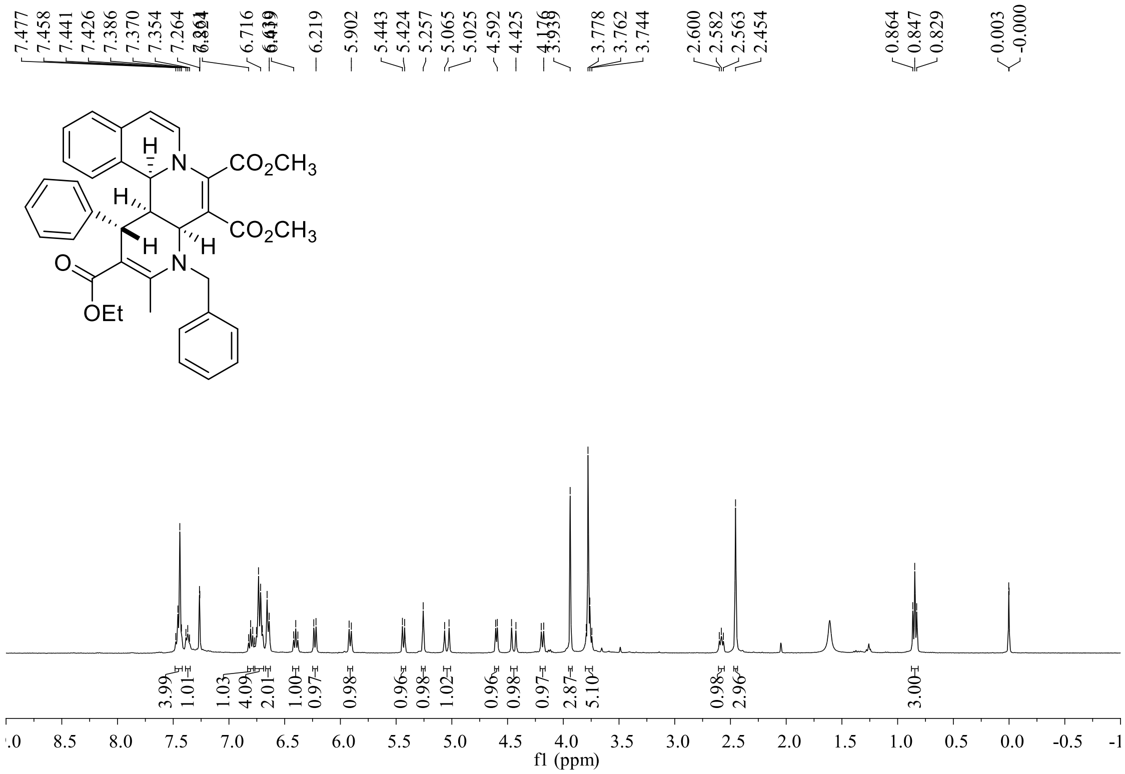

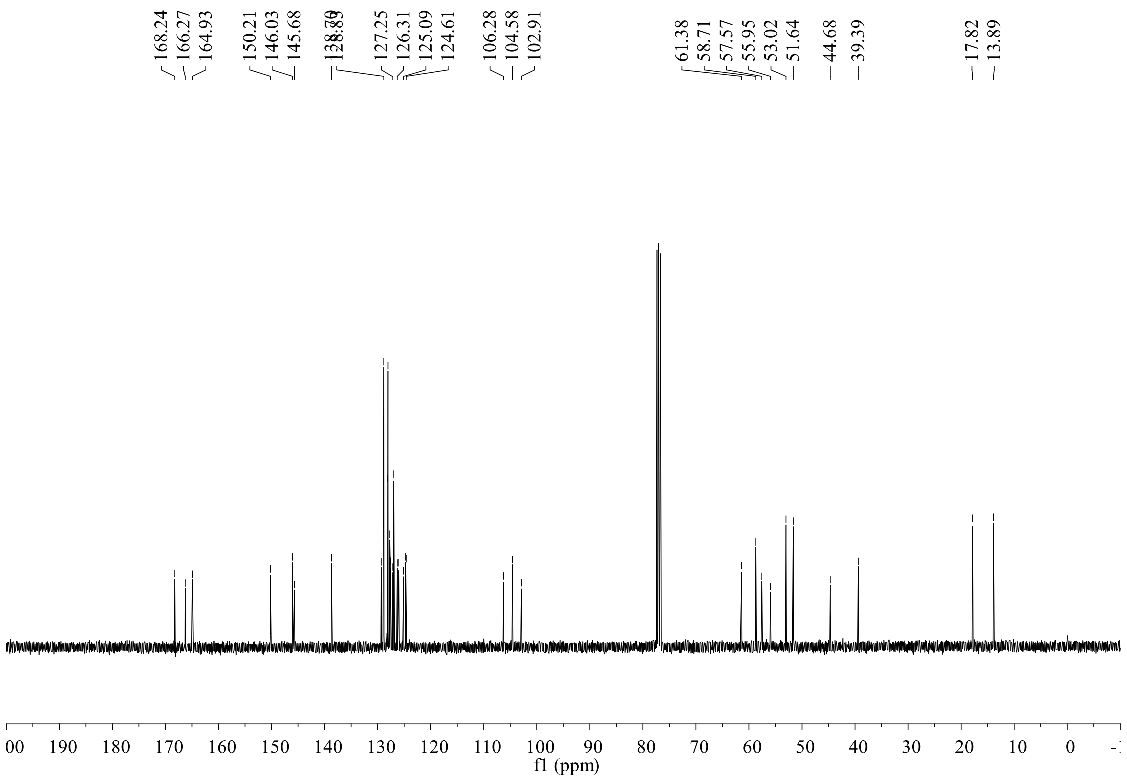
168.24
166.27
164.93
150.21
146.03
145.68
127.25
126.31
125.09
124.61
106.28
104.58
102.91
61.38
58.71
57.57
55.95
53.02
51.64
44.68
39.39
17.82
13.89
f1 (ppm)

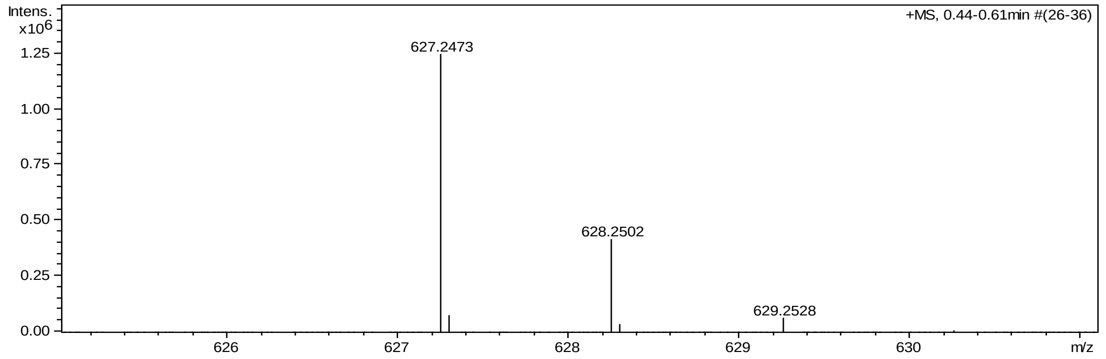
Intens.
x10^6
+MS, 0.44-0.61min #(26-36)
627.2473
628.2502
629.2528
m/z

Trimethyl 4-benzyl-1-(4-fluorophenyl)-3-methyl-4,4a,13b,13c-tetrahydro-1*H*-isoquinolino[1,2-*f*][1,6]naphthyridine-2,5,6-tricarboxylate (4c): orange solid, 58%, m.p. 218-220 ºC; ^{1}H NMR (400 MHz, $CDCl_3$) δ 7.48-7.37 (m, 5H, ArH), 6.84 (t, *J* = 7.6 Hz, 1H, ArH), 6.72 (t, *J* = 7.6 Hz, 1H, ArH), 6.58-6.54 (m, 2H, ArH), 6.46 (d, *J* = 7.6 Hz, 1H, ArH), 6.40 (t, *J* = 8.0 Hz, 2H, ArH), 6.21 (d, *J* = 8.0 Hz, 1H, ArH), 5.89 (d, *J* = 7.6 Hz, 1H, CH), 5.42 (d, *J* = 8.0 Hz, 1H, CH), 5.26 (s, 1H, CH), 5.04 (d, *J* = 15.6 Hz, 1H, CH_2), 4.61 (d, *J* = 6.0 Hz, 1H, CH), 4.44 (d, *J* = 15.6 Hz, 1H, CH_2), 4.15 (d, *J* = 8.4 Hz, 1H, CH), 3.94 (s, 3H, OCH_3), 3.78 (s, 3H, OCH_3), 3.30 (s, 3H, OCH_3), 2.49 (t, *J* = 7.6 Hz, 1H, CH), 2.45 (s, 3H, CH_3) ppm. ^{13}C NMR (100 MHz, $CDCl_3$) δ 168.9, 166.2, 164.8, 161.6, 159.2, 150.2, 145.6, 141.8, 141.8, 138.6, 129.3, 129.2, 128.9, 128.1, 127.9, 127.4, 126.4, 126.1, 125.1, 124.7, 113.8, 113.6, 106.1, 104.6, 102.7, 61.2, 57.4, 56.0, 53.1, 51.7, 50.1, 44.6, 38.7, 18.0 ppm. IR (KBr) υ: 3756, 3021, 2932, 2873, 2012, 1723, 1638, 1526, 1312, 1247, 1132, 1034, 965, 865, 743 cm^{-1}; MS (*m/z*): HRMS (ESI) Calcd. for $C_{36}H_{34}NaN_2O_5$ ([M+Na]$^+$): 631.2215, found: 631.2218.

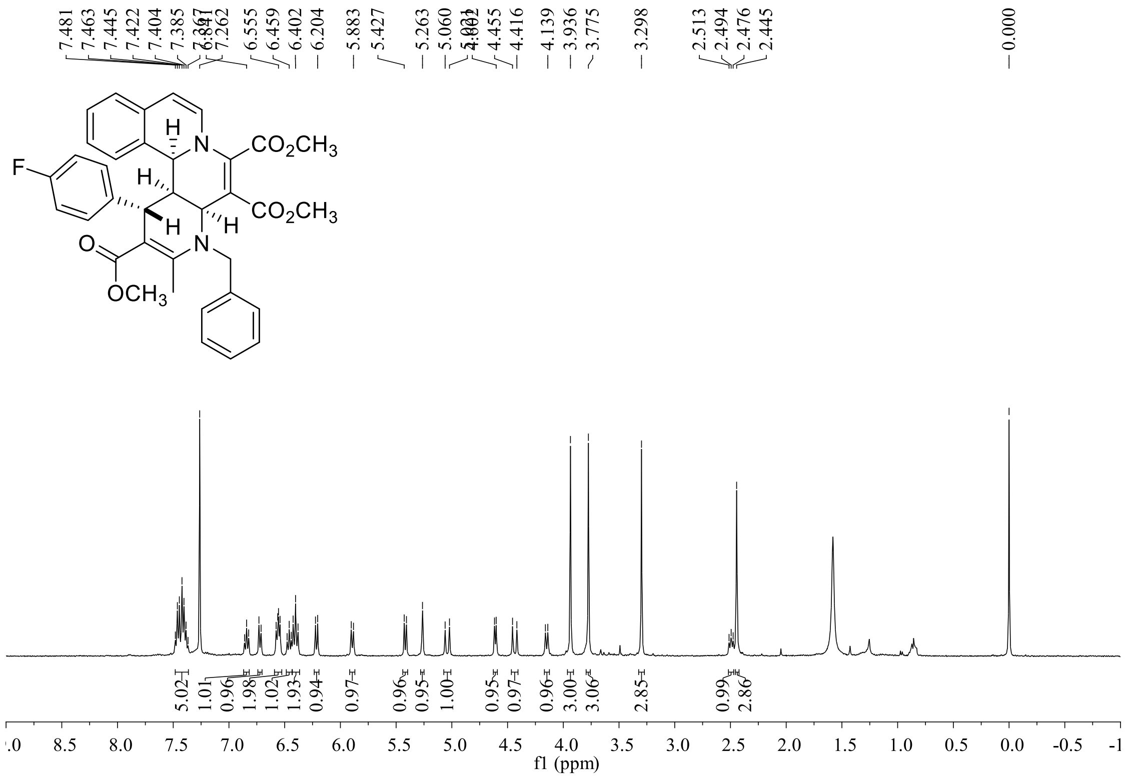

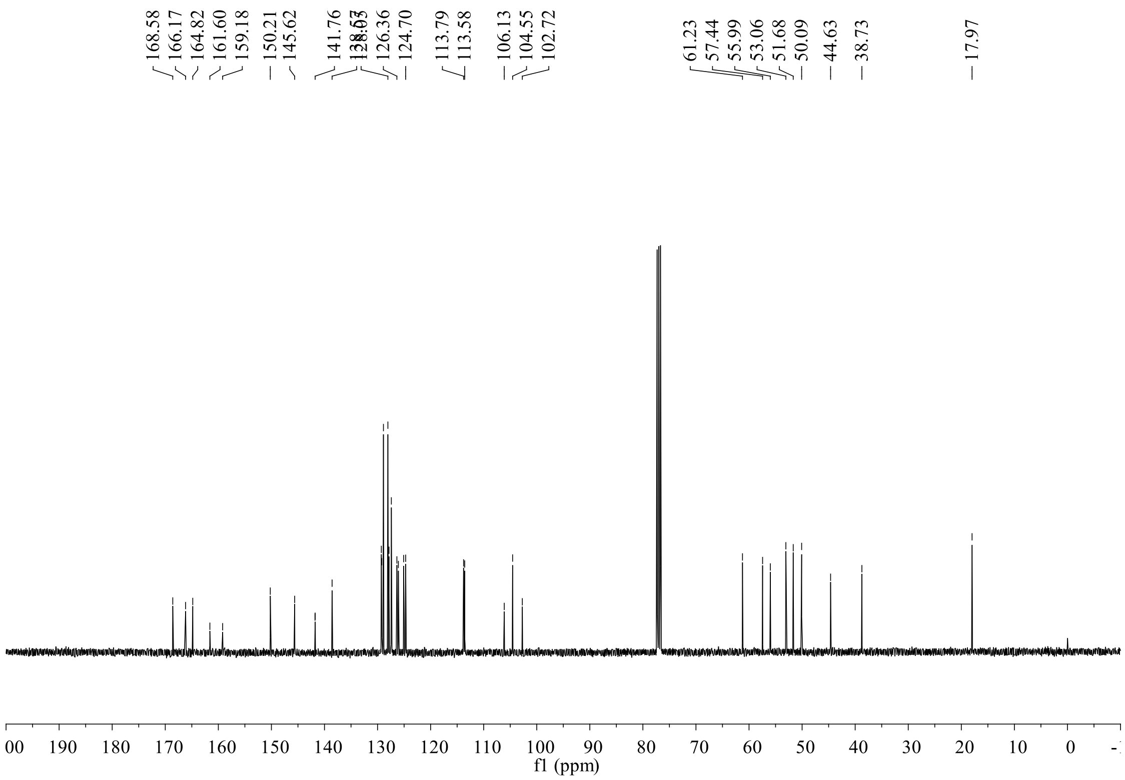
168.58
166.17
164.82
161.60
159.18
150.21
145.62
141.76
126.36
124.70
113.79
113.58
106.13
104.55
102.72
61.23
57.44
55.99
53.06
51.68
50.09
44.63
38.73
17.97
f1 (ppm)

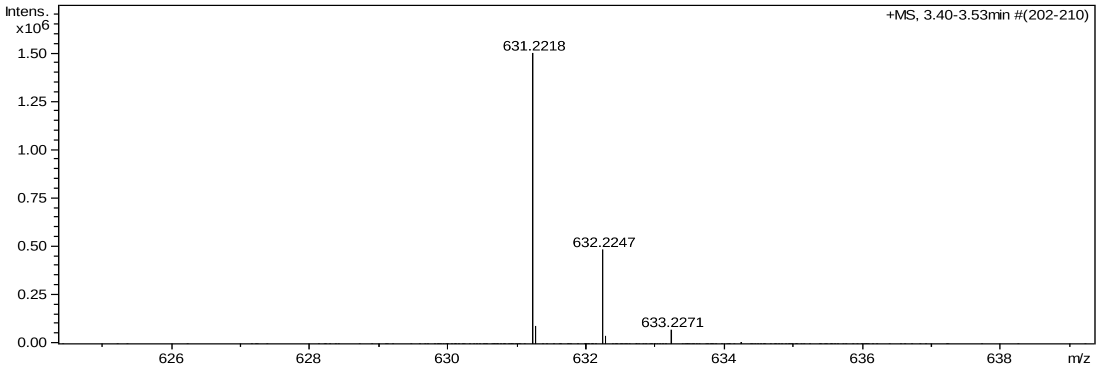
Intens.
x10^6
+MS, 3.40-3.53min #(202-210)
631.2218
632.2247
633.2271
m/z

Trimethyl 4-benzyl-1-(4-methoxyphenyl)-3-methyl-4,4a,13b,13c-tetrahydro-1*H*-isoquinolino[1,2-*f*][1,6]naphthyridine-2,5,6-tricarboxylate (4d): orange solid, 88%, m.p. 169-171 °C; ^{1}H NMR (400 MHz, $CDCl_3$) δ 7.48-7.41 (m, 4H, ArH), 7.37 (t, *J* = 7.2 Hz, 1H, ArH), 6.81 (t, *J* = 7.6 Hz, 1H, ArH), 6.72 (t, *J* = 7.6 Hz, 1H, ArH), 6.54 (d, *J* = 8.4 Hz, 2H, ArH), 6.45 (t, *J* = 8.0 Hz, 1H, ArH), 6.28 (t, *J* = 8.4 Hz, 2H, ArH), 6.21 (d, *J* = 8.0 Hz, 1H, ArH), 5.91 (d, *J* = 7.6 Hz, 1H, CH), 5.42 (d, *J* = 8.0 Hz, 1H, CH), 5.25 (s, 1H, CH), 5.03 (d, *J* = 16.0 Hz, 1H, CH_2), 4.58 (d, *J* = 6.0 Hz, 1H, CH), 4.44 (d, *J* = 16.0 Hz, 1H, CH_2), 4.12 (d, *J* = 8.8 Hz, 1H, CH), 3.94 (s, 3H, OCH_3), 3.77 (s, 3H, OCH_3), 3.63 (s, 3H, OCH_3), 3.31 (s, 3H, OCH_3), 2.54 (t, *J* = 7.6 Hz, 1H, CH), 2.43 (s, 3H, CH_3) ppm. ^{13}C NMR (100 MHz, $CDCl_3$) δ 168.8, 166.3, 164.9, 156.8, 149.8, 145.6, 138.7, 138.1, 129.3, 128.8, 128.0, 127.7, 127.6, 127.2, 126.3, 126.1, 125.2, 124.6, 112.6, 106.5, 104.6, 102.8, 61.3, 57.5, 55.9, 55.1, 53.0, 51.6, 50.1, 44.6, 38.6, 17.9 ppm. IR (KBr) υ: 3722, 3013, 2923, 2823, 1943, 1817, 1672, 1536, 1422, 1157, 1045, 1021, 927, 836 cm^{-1}; MS (*m*/*z*): HRMS (ESI) Calcd. for $C_{37}H_{36}NaN_2O_7$ ($[M+Na]^+$): 643.2420, found: 643.2421.

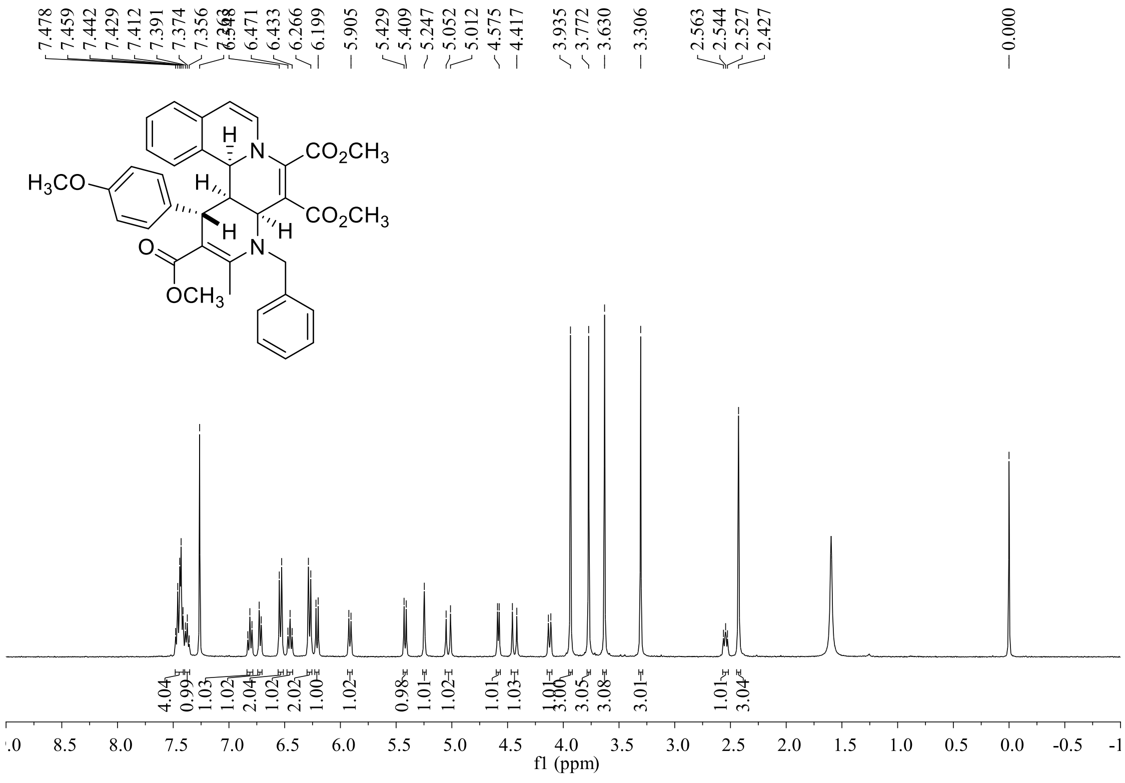

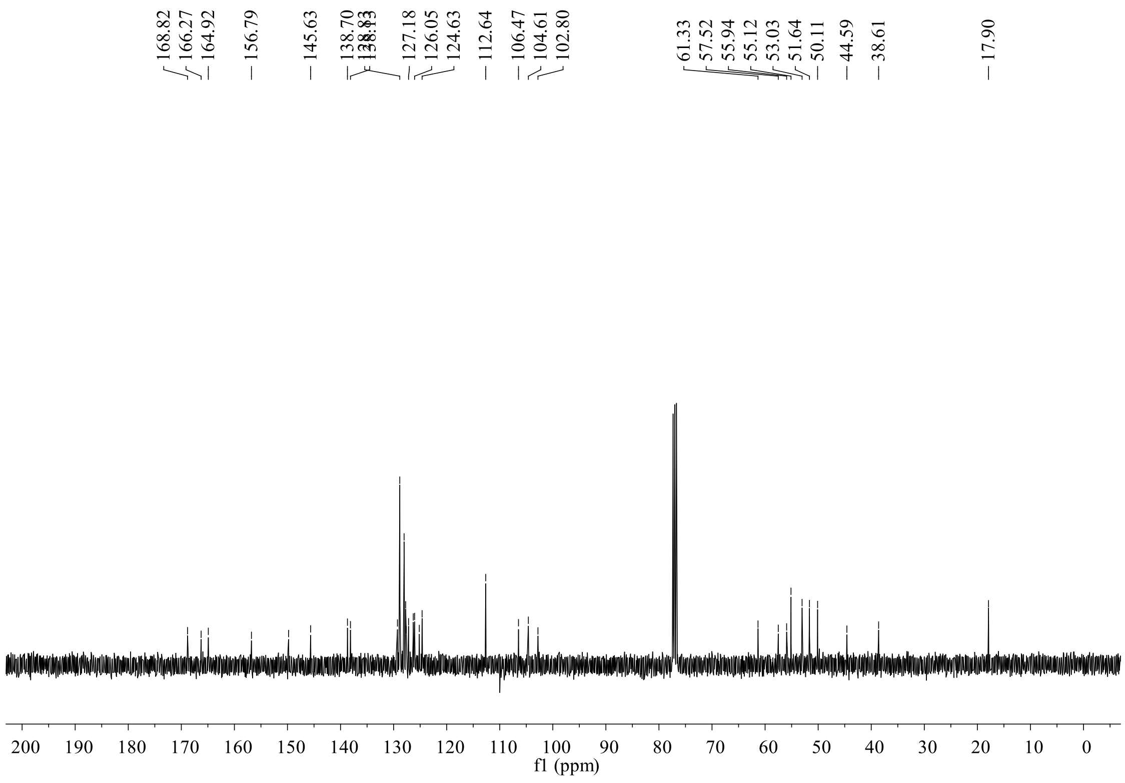

168.82
166.27
164.92
156.79
145.63
138.70
138.13
127.18
126.05
124.63
112.64
106.47
104.61
102.80
61.33
57.52
55.94
55.12
53.03
51.64
50.11
44.59
38.61
17.90
200 190 180 170 160 150 140 130 120 110 100 90 80 70 60 50 40 30 20 10 0
f1 (ppm)

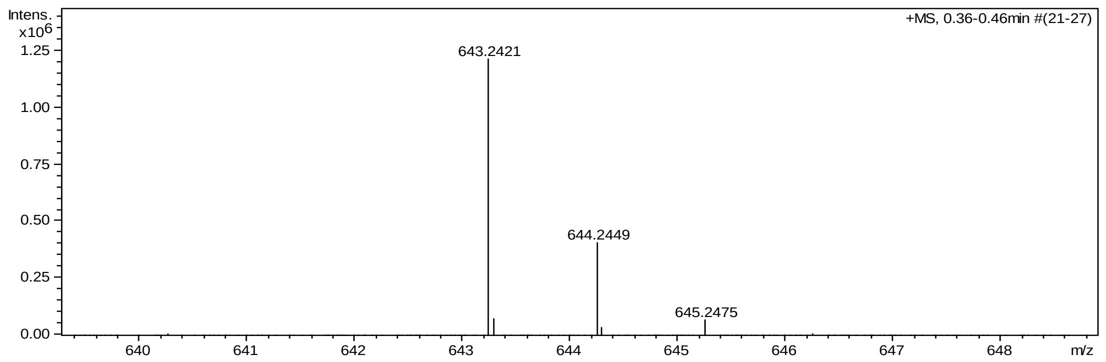

Intens.
x10^6
+MS, 0.36-0.46min #(21-27)
643.2421
644.2449
645.2475
1.25
1.00
0.75
0.50
0.25
0.00
640 641 642 643 644 645 646 647 648 m/z

5,6-Diethyl 2-methyl 4-benzyl-3-methyl-1-phenyl-4,4a,13b,13c-tetrahydro-1*H*-isoquinolino[1,2-*f*][1,6]naphthyridine-2,5,6-tricarboxylate (4e): orange solid, 80%, m.p. 175-177 °C; ^{1}H NMR (400 MHz, $CDCl_3$) δ 7.48-7.42 (m, 4H, ArH), 7.40-7.36 (m, 1H, ArH), 6.79 (t, *J* = 7.6 Hz, 1H, ArH), 6.75-6.70 (m, 4H, ArH), 6.63-6.61 (m, 2H, ArH), 6.38 (t, *J* = 7.2 Hz, 1H, ArH), 6.26 (t, *J* = 8.0 Hz, 1H, ArH), 5.89 (d, *J* = 7.6 Hz, 1H, CH), 5.41 (d, *J* = 8.0 Hz, 1H, CH), 5.25 (s, 1H, CH), 5.04 (d, *J* = 15.6 Hz, 1H, CH_2), 4.61 (d, *J* = 6.0 Hz, 1H, CH), 4.45 (d, *J* = 16.0 Hz, 1H, CH_2), 4.43-4.34 (m, 2H, OCH_2), 4.29-4.19 (m, 2H, OCH_2), 4.18 (d, *J* = 8.4 Hz, 1H, CH), 3.29 (s, 3H, OCH_3), 2.56 (t, *J* = 7.6 Hz, 1H, CH), 2.46 (s, 3H, CH_3), 1.42-1.34 (m, 6H, CH_3) ppm. ^{13}C NMR (100 MHz, $CDCl_3$) δ 168.7, 165.9, 164.3, 150.2, 146.1, 145.6, 138.6, 129.5, 128.8, 128.1, 128.0, 127.8, 127.6, 127.2, 127.0, 126.2, 126.1, 125.1, 124.7, 124.5, 106.5, 104.3, 103.1, 62.3, 61.3, 60.4, 57.5, 56.1, 50.0, 44.8, 39.4, 17.9, 14.4, 13.9 ppm. IR (KBr) υ: 3785, 3045, 2982, 2901, 2012, 1787, 1648, 1526, 1412, 1247, 1097, 957, 863, 769 cm^{-1}; MS (*m*/*z*): HRMS (ESI) Calcd. for $C_{38}H_{38}NaN_2O_6$ ([M+Na]$^+$): 641.2628, found: 641.2632.

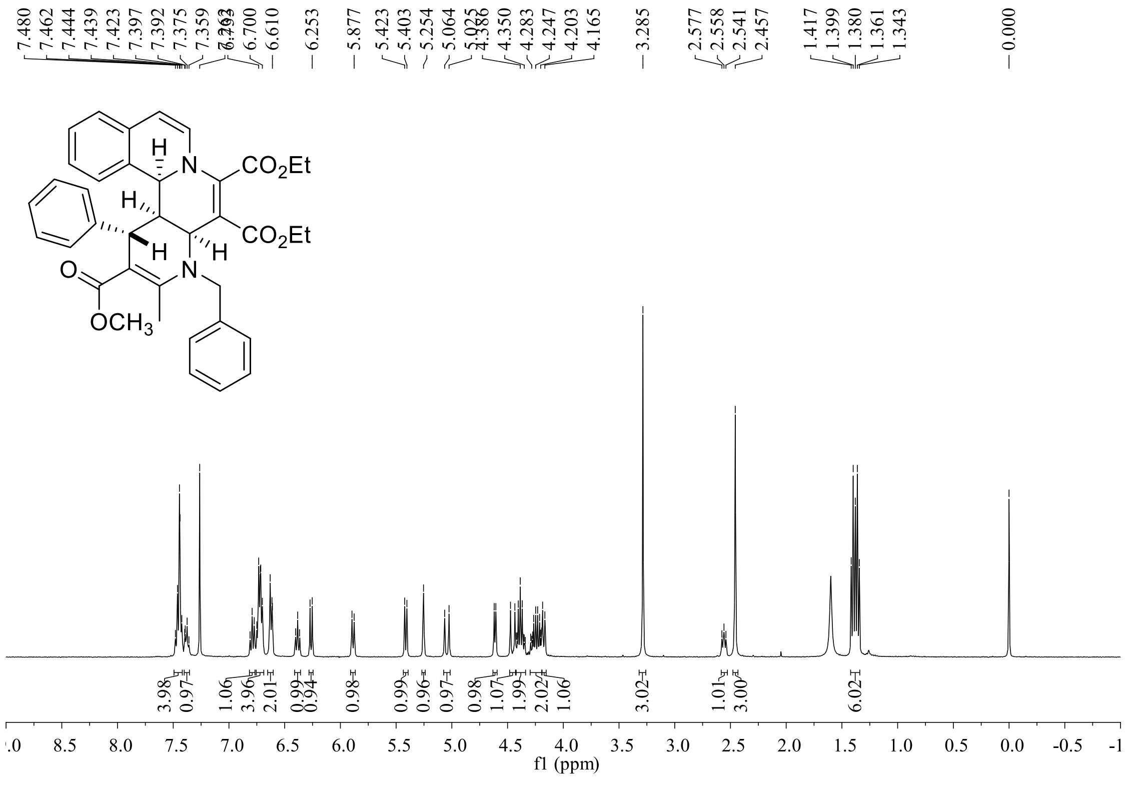

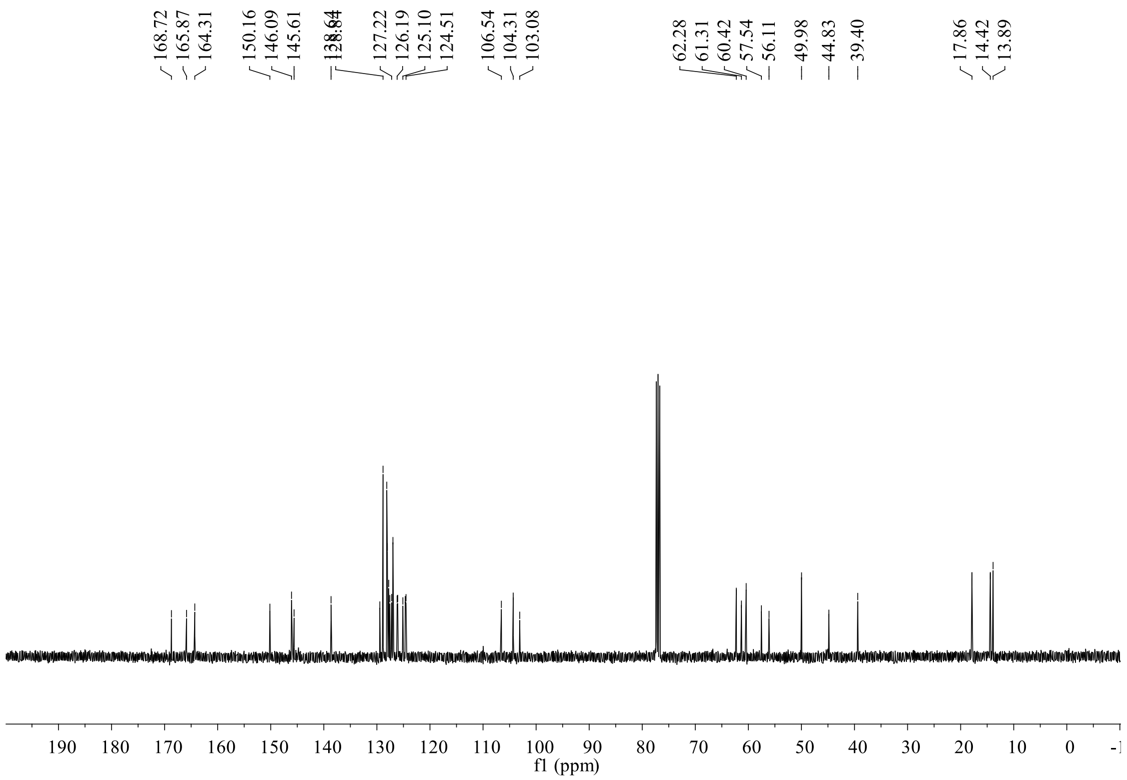
168.72
165.87
164.31
150.16
146.09
145.61
127.22
126.19
125.10
124.51
106.54
104.31
103.08
62.28
61.31
60.42
57.54
56.11
49.98
44.83
39.40
17.86
14.42
13.89
190 180 170 160 150 140 130 120 110 100 90 80 70 60 50 40 30 20 10 0
f1 (ppm)

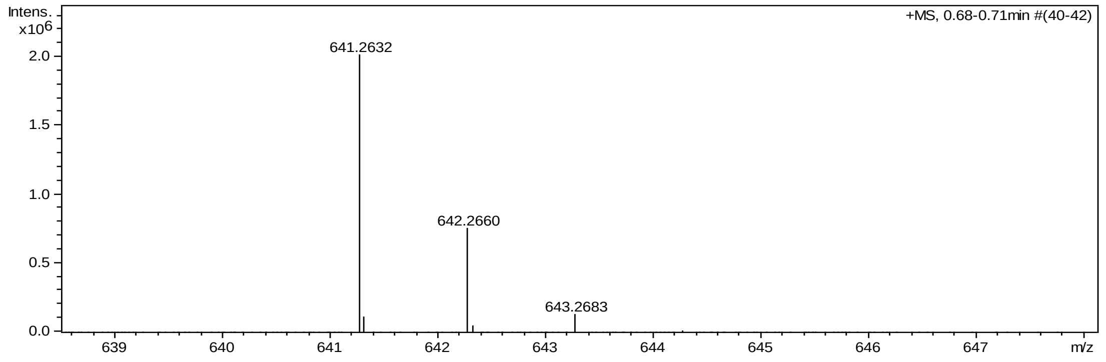
Intens.
x10^6
+MS, 0.68-0.71min #(40-42)
641.2632
642.2660
643.2683
2.0
1.5
1.0
0.5
0.0
639 640 641 642 643 644 645 646 647
m/z

Triethyl 4-benzyl-3-methyl-1-phenyl-4,4a,13b,13c-tetrahydro-1*H*-isoquinolino[1,2-*f*][1,6]naphthyridine-2,5,6-tricarboxylate (4f): orange solid, 82%, m.p. 174-176 ºC; ^{1}H NMR (400 MHz, $CDCl_3$) δ 7.48-7.42 (m, 4H, ArH), 7.39-7.35 (m, 1H, ArH), 6.80 (t, *J* = 7.6 Hz, 1H, ArH), 6.75-6.69 (m, 4H, ArH), 6.63 (d, *J* = 6.4 Hz, 2H, ArH), 6.39 (t, *J* = 7.6 Hz, 1H, ArH), 6.27 (d, *J* = 8.0 Hz, 1H, ArH), 5.88 (d, *J* = 7.6 Hz, 1H, CH), 5.42 (d, *J* = 8.0 Hz, 1H, CH), 5.25 (s, 1H, CH), 5.05 (d, *J* = 16.0 Hz, 1H, CH_2), 4.60 (d, *J* = 6.0 Hz, 1H, CH), 4.44 (d, *J* = 15.6 Hz, 1H, CH_2), 4.41-4.34 (m, 2H, OCH_2), 4.29-4.21 (m, 2H, OCH_2), 4.19 (d, *J* = 8.0 Hz, 1H, CH), 3.80-3.72 (m, 2H, OCH_2), 2.53 (t, *J* = 7.2 Hz, 1H, CH), 2.47 (s, 3H, CH_3), 1.41-1.34 (m, 6H, CH_3), 0.86 (t, *J* = 7.2 Hz, 3H, CH_3) ppm. ^{13}C NMR (100 MHz, $CDCl_3$) δ 168.3, 165.9, 164.4, 150.1, 146.1, 145.6, 138.7, 129.4, 128.8, 128.2, 128.1, 127.7, 127.6, 127.2, 126.9, 126.2, 126.0, 125.1, 124.7, 124.5, 106.5, 104.3, 103.2, 62.2, 61.3, 60.4, 58.7, 57.6, 56.1, 44.7, 39.4, 17.8, 14.4, 13.9, 13.8 ppm. IR (KBr) υ: 3722, 3053, 2912, 2853, 1965, 1727, 1688, 1523, 1372, 1247, 1127, 1040, 957, 759 cm^{-1}; MS (*m/z*): HRMS (ESI) Calcd. for $C_{39}H_{40}NaN_2O_6$ ($[M+Na]^+$): 655.2784, found: 655.2774.

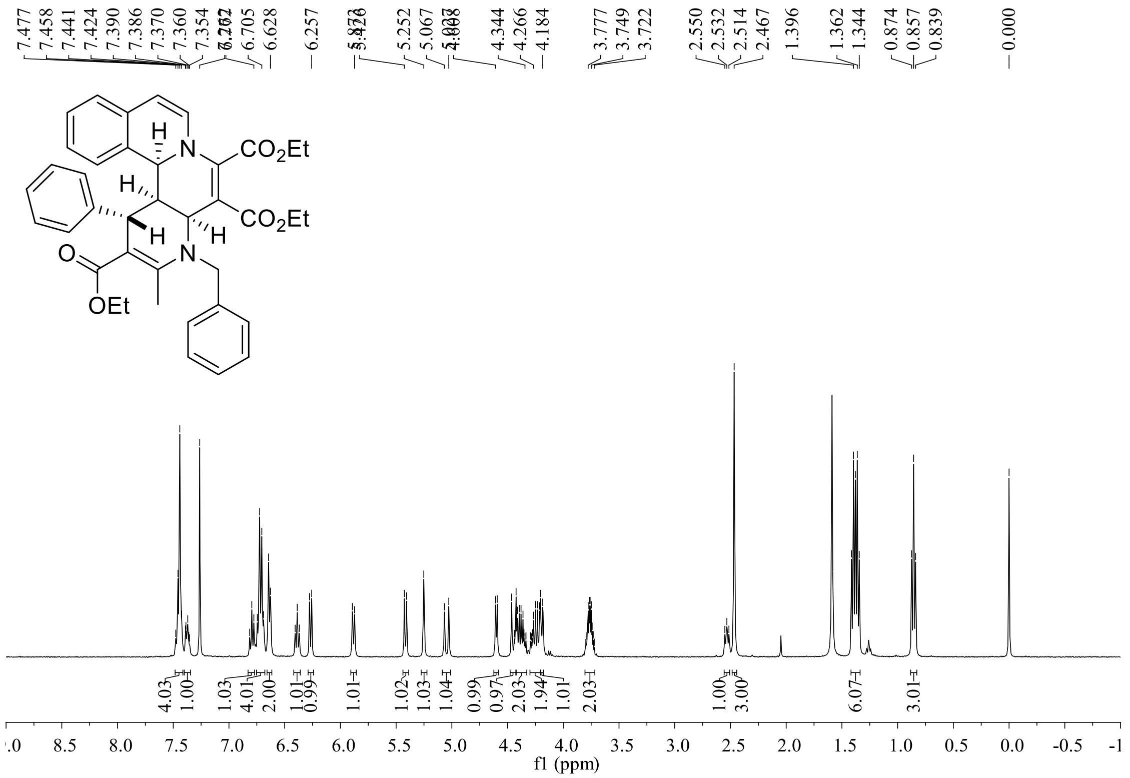

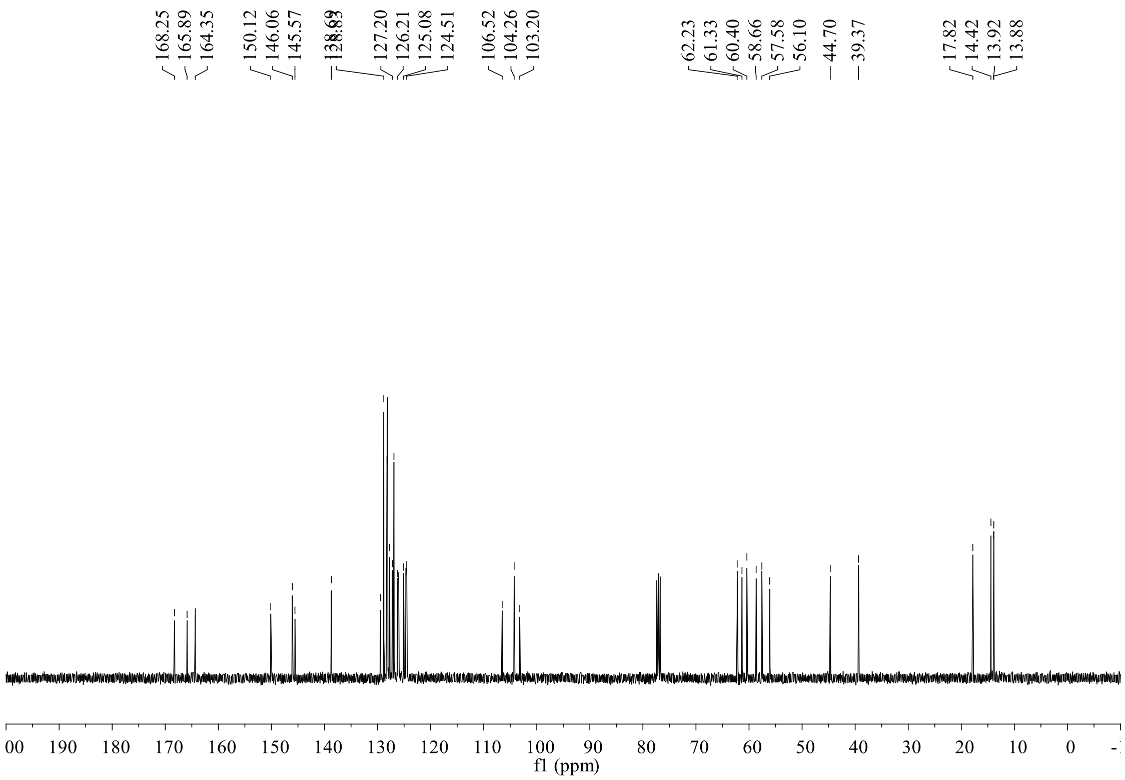
168.25
165.89
164.35
150.12
146.06
145.57
128.69
128.53
127.20
126.21
125.08
124.51
106.52
104.26
103.20
62.23
61.33
60.40
58.66
57.58
56.10
44.70
39.37
17.82
14.42
13.92
13.88
f1 (ppm)

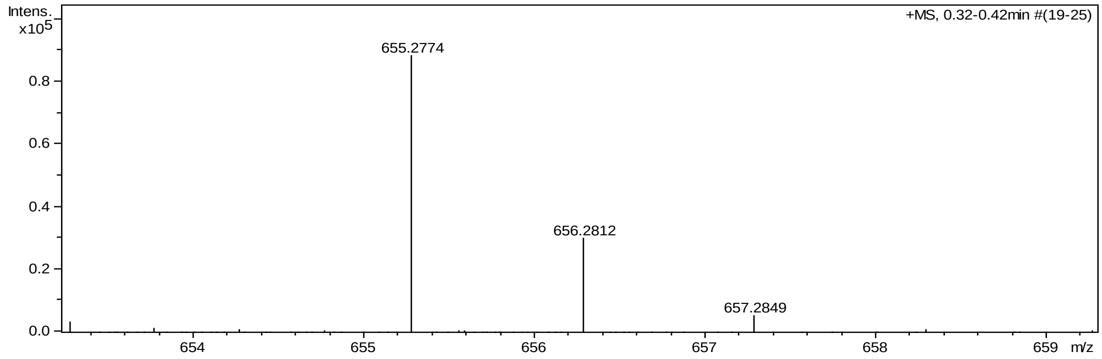
Intens.
x10^5
+MS, 0.32-0.42min #(19-25)
655.2774
656.2812
657.2849
m/z

Trimethyl 4-(4-methoxybenzyl)-3-methyl-1-phenyl-4,4a,13b,13c-tetrahydro-1*H*-isoquinolino[1,2-*f*][1,6]naphthyridine-2,5,6-tricarboxylate (4g): orange solid, 84%, m.p. 199-201 ºC; ^{1}H NMR (400 MHz, $CDCl_3$) δ 7.35 (d, *J* = 8.4 Hz, 2H, ArH), 6.98 (d, *J* = 8.4 Hz, 2H, ArH), 6.81 (t, *J* = 7.6 Hz, 1H, ArH), 6.76-6.72 (m, 4H, ArH), 6.61 (d, *J* = 6.4 Hz, 2H, ArH), 6.41 (t, *J* = 7.6 Hz, 1H, ArH), 6.22 (d, *J* = 8.0 Hz, 1H, ArH), 5.94 (d, *J* = 8.0 Hz, 1H, CH), 5.43 (d, *J* = 8.0 Hz, 1H, CH), 5.26 (s, 1H, CH), 4.97 (d, *J* = 15.6 Hz, 1H, CH_2), 4.59 (d, *J* = 6.0 Hz, 1H, CH), 4.39 (d, *J* = 15.2 Hz, 1H, CH_2), 3.93 (s, 3H, OCH_3), 3.86 (s, 3H, OCH_3), 3.77 (s, 3H, OCH_3), 3.29 (s, 3H, OCH_3), 2.60 (t, *J* = 7.6 Hz, 1H, CH), 2.44 (s, 3H, CH_3) ppm. ^{13}C NMR (100 MHz, $CDCl_3$) δ 168.7, 166.3, 164.9, 159.2, 150.4, 146.2, 145.7, 130.7, 129.3, 128.0, 127.5, 127.3, 127.0, 126.3, 126.1, 125.2, 124.7, 124.6, 114.2, 106.3, 104.6, 102.9, 61.4, 57.3, 55.4, 55.3, 53.0, 51.6, 50.0, 45.0, 39.4, 17.9 ppm. IR (KBr) υ: 3742, 3063, 2947, 2842, 1947, 1787, 1638, 1526, 1392, 1117, 1097, 1020, 987, 876, 739 cm^{-1}; MS (*m*/*z*): HRMS (ESI) Calcd. for $C_{37}H_{36}NaN_2O_7$ ($[M+Na]^+$): 643.2420, found: 643.2420.

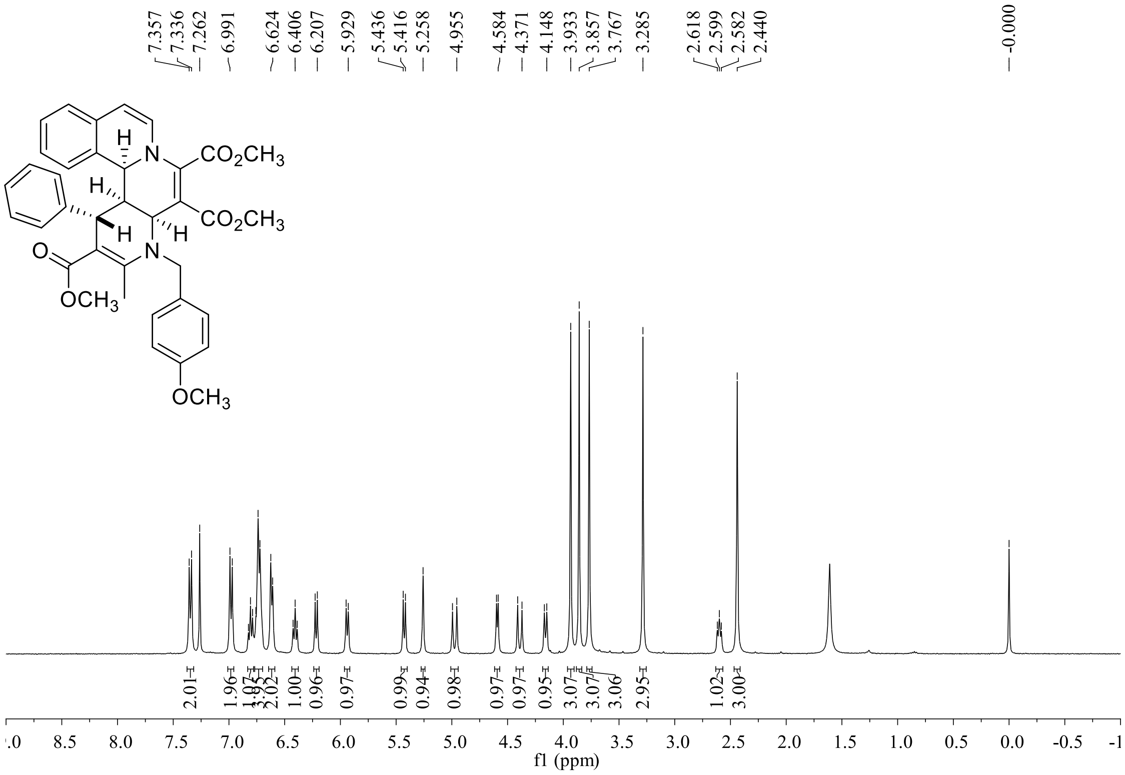

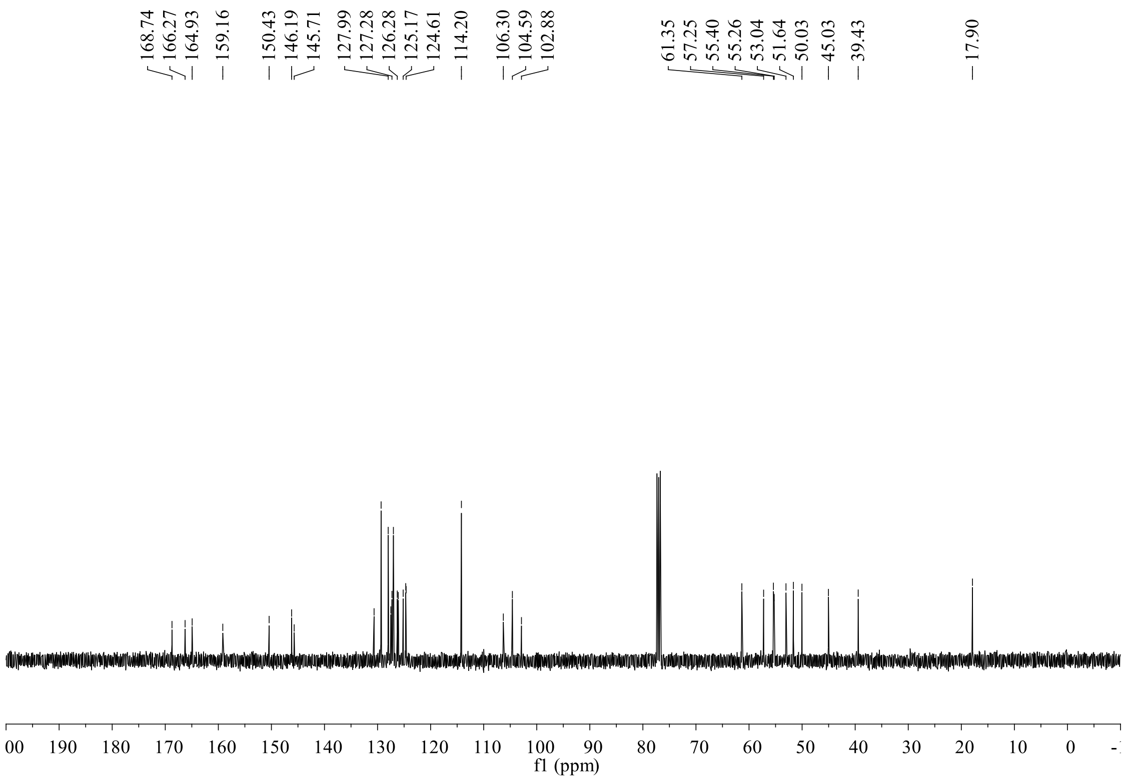
168.74
166.27
164.93
159.16
150.43
146.19
145.71
127.99
127.28
126.28
125.17
124.61
114.20
106.30
104.59
102.88
61.35
57.25
55.40
55.26
53.04
51.64
50.03
45.03
39.43
17.90
f1 (ppm)

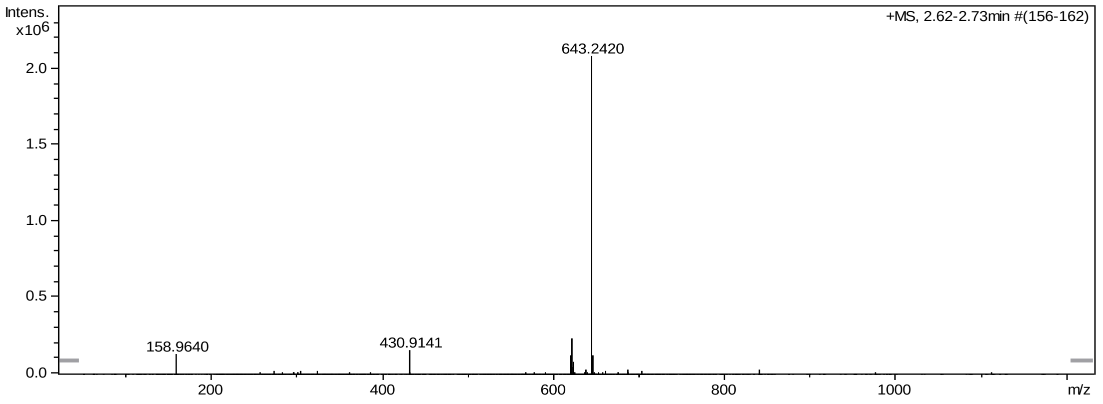
Intens.
x10^6
+MS, 2.62-2.73min #(156-162)
643.2420
158.9640
430.9141
m/z

Trimethyl 4-(3,4-dimethoxyphenethyl)-3-methyl-1-phenyl-4,4a,13b,13c-tetrahydro-1*H*-isoquinolino[1,2-*f*][1,6]naphthyridine-2,5,6-tricarboxylate (4h): orange solid, 88%, m.p. 191-193 °C; ^{1}H NMR (400 MHz, $CDCl_3$) δ 6.92-6.90 (m, 1H, ArH), 6.85 (s, 1H, ArH), 6.84-6.81 (m, 2H, ArH), 6.79-6.78 (m, 3H, ArH), 6.75-6.69 (m, 3H, ArH), 6.47-6.43 (m, 1H, ArH), 6.20 (d, *J* = 8.0 Hz, 1H, ArH), 6.00 (d, *J* = 8.0 Hz, 1H, CH), 5.43 (d, *J* = 8.0 Hz, 1H, CH), 5.20 (s, 1H, CH), 4.42 (d, *J* = 6.0 Hz, 1H, CH), 4.21-4.16 (m, 1H, CH_2), 4.14 (d, *J* = 8.4 Hz, 1H, CH), 3.92 (s, 3H, OCH_3), 3.88 (s, 3H, OCH_3), 3.84 (s, 3H, OCH_3), 3.70 (s, 3H, OCH_3), 3.68-3.62 (m, 1H, CH_2), 3.27 (s, 3H, OCH_3), 3.01-2.95 (m, 1H, CH_2), 2.92-2.87 (m, 1H, CH_2), 2.52 (t, *J* = 8.0 Hz, 1H, CH), 2.45 (s, 3H, CH_3) ppm. ^{13}C NMR (100 MHz, $CDCl_3$) δ 168.9, 166.2, 164.9, 150.3, 149.1, 147.8, 146.4, 145.7, 131.4, 129.3, 128.0, 127.6, 127.3, 127.0, 126.3, 126.1, 125.3, 124.8, 124.6, 120.9, 112.0, 111.3, 105.3, 104.6, 102.6, 61.5, 59.2, 55.9, 55.8, 54.1, 53.0, 51.6, 50.0, 45.3, 39.5, 36.7, 17.0 ppm. IR (KBr) υ: 3727, 3056, 2990, 2944, 2903, 2835, 2585, 1741, 1680, 1575, 1515, 1437, 1374, 1231, 1147, 1101, 1020, 976, 872, 823 cm^{-1}; MS (*m*/*z*): HRMS (ESI) Calcd. for $C_{39}H_{40}NaN_2O_8$ ([M+Na]$^+$): 687.2682, found: 687.2663.

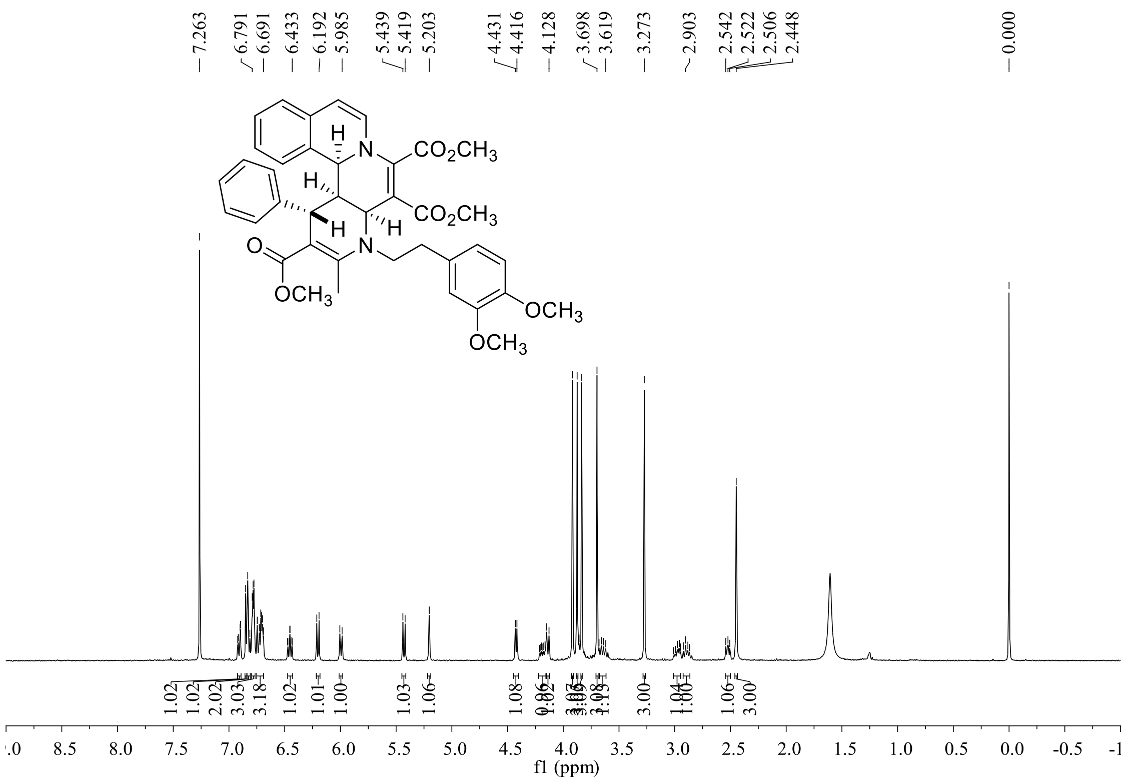

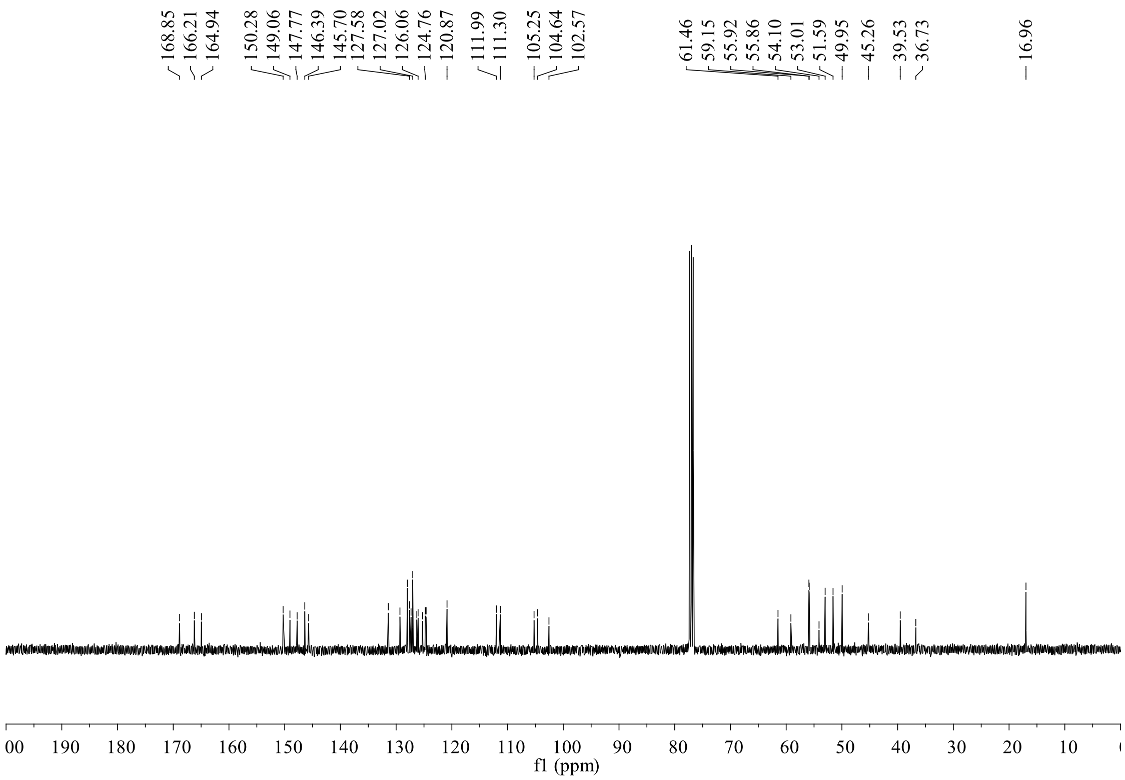
168.85
166.21
164.94
150.28
149.06
147.77
146.39
145.70
127.58
127.02
126.06
124.76
120.87
111.99
111.30
105.25
104.64
102.57
61.46
59.15
55.92
55.86
54.10
53.01
51.59
49.95
45.26
39.53
36.73
16.96
f1 (ppm)

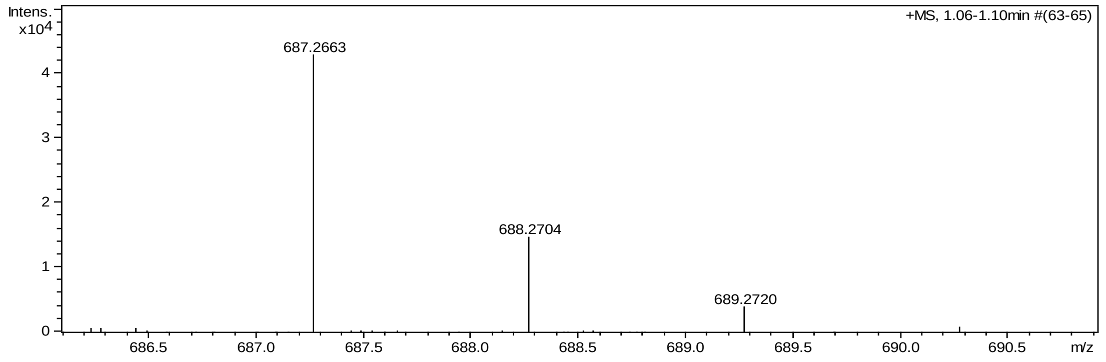
Intens.
x10^4
+MS, 1.06-1.10min #(63-65)
687.2663
688.2704
689.2720
m/z

Trimethyl 4-benzyl-9-bromo-3-methyl-1-phenyl-4,4a,13b,13c-tetrahydro-1*H*-isoquinolino[1,2-*f*][1,6]naphthyridine-2,5,6-tricarboxylate (4i): orange solid, 78%, m.p. 196-198 ºC; ^{1}H NMR (400 MHz, $CDCl_3$) δ 7.48-7.43 (m, 3H, ArH), 7.41-7.36 (m, 2H, ArH), 7.17 (d, *J* = 8.0 Hz, 1H, ArH), 6.88 (t, *J* = 7.6 Hz, 1H, ArH), 6.75-6.73 (m, 3H, ArH), 6.62 (d, *J* = 6.0 Hz, 2H, ArH), 6.58 (s, 1H, ArH), 6.45 (t, *J* = 7.6 Hz, 1H, CH), 5.87 (d, *J* = 8.0 Hz, 1H, CH), 5.28 (s, 1H, CH), 5.03 (d, *J* = 16.0 Hz, 1H, CH_2), 4.57 (d, *J* = 6.0 Hz, 1H, CH), 4.42 (d, *J* = 15.6 Hz, 1H, CH_2), 4.15 (d, *J* = 8.8 Hz, 1H, CH), 3.96 (s, 3H, OCH_3), 3.78 (s, 3H, OCH_3), 3.29 (s, 3H, OCH_3), 2.55 (t, *J* = 7.6 Hz, 1H, CH), 2.44 (s, 3H, CH_3) ppm. ^{13}C NMR (100 MHz, $CDCl_3$) δ 168.6, 166.0, 164.4, 150.1, 145.6, 144.1, 138.4, 128.9, 128.5, 128.1, 128.0, 127.8, 127.6, 127.3, 127.2, 127.1, 124.9, 124.7, 106.3, 104.7, 99.8, 61.5, 57.2, 55.8, 53.3, 51.8, 50.1, 44.6, 39.4, 17.9 ppm. IR (KBr) υ: 3721, 3012, 2932, 2823, 1976, 1741, 1645, 1523, 1327, 1127, 1027, 957, 826 cm^{-1}; MS (*m*/*z*): HRMS (ESI) Calcd. for $C_{36}H_{33}BrNaN_2O_6$ ($[M+Na]^+$): 691.1420, found: 691.1423.

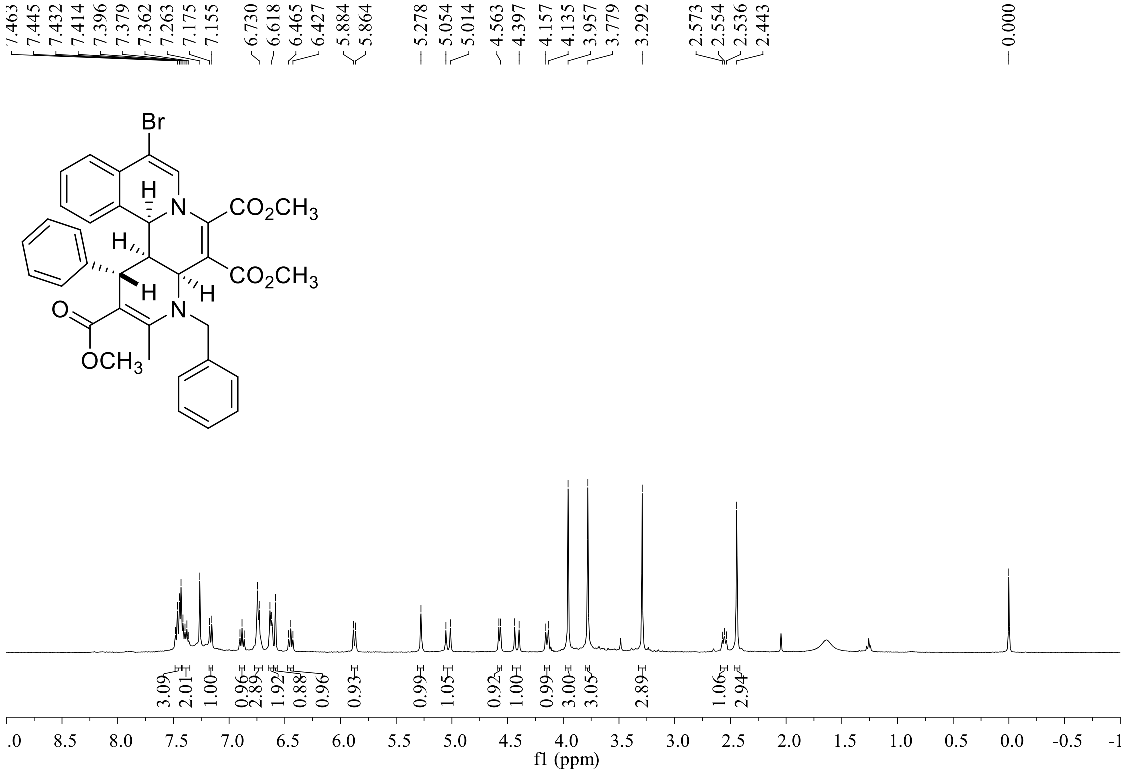

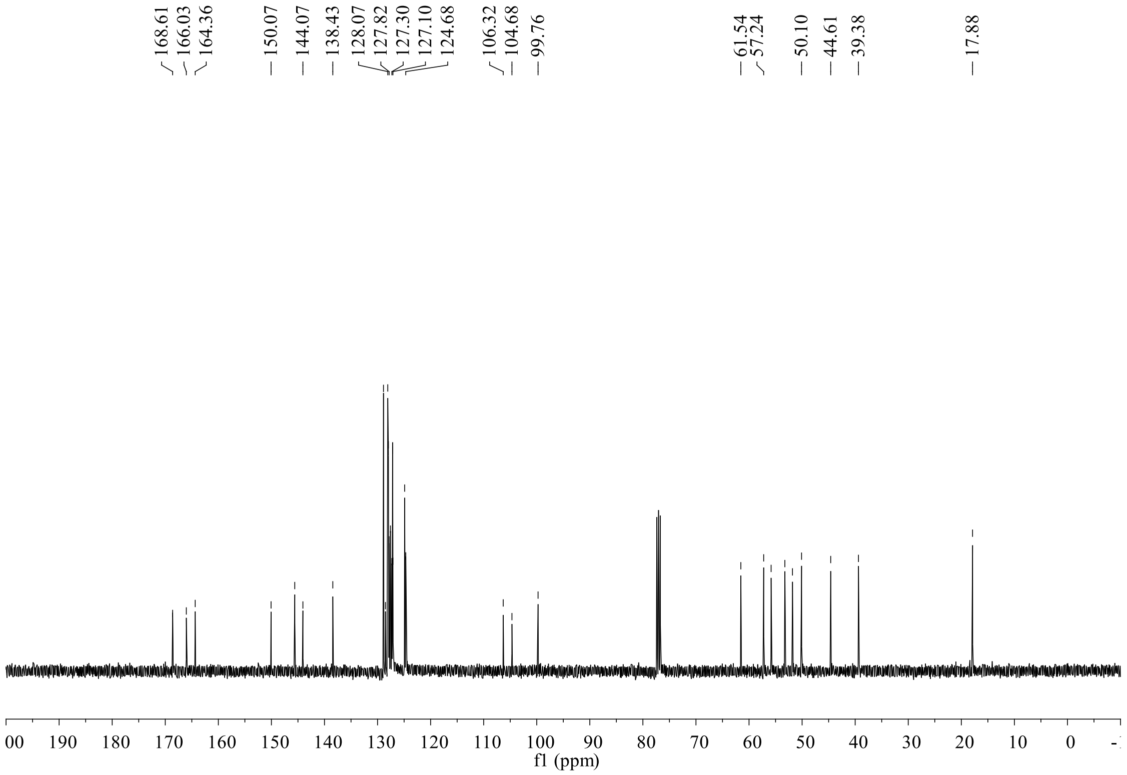
168.61
166.03
164.36
150.07
144.07
138.43
128.07
127.82
127.30
127.10
124.68
106.32
104.68
99.76
61.54
57.24
50.10
44.61
39.38
17.88
f1 (ppm)

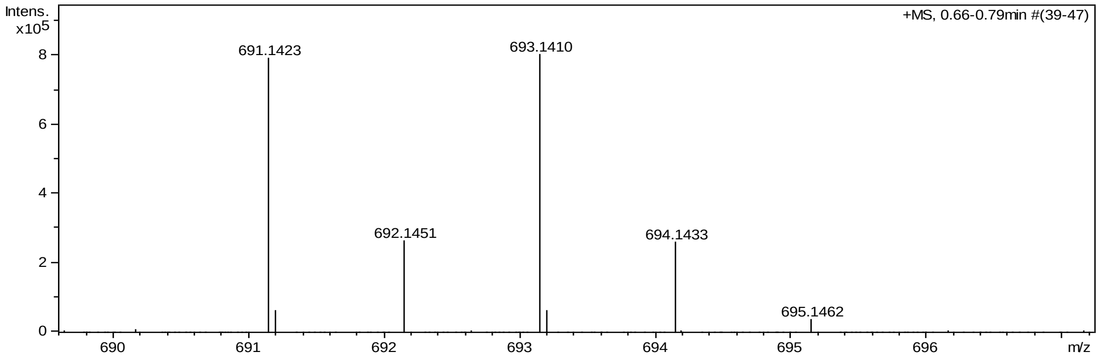
Intens.
x10^5
+MS, 0.66-0.79min #(39-47)
691.1423
693.1410
692.1451
694.1433
695.1462
m/z

Trimethyl 4-benzyl-10-bromo-3-methyl-1-phenyl-4,4a,13b,13c-tetrahydro-1*H*-isoquinolino[1,2-*f*][1,6]naphthyridine-2,5,6-tricarboxylate (4j): orange solid, 67%, m.p. 197-199 ºC; ^{1}H NMR (400 MHz, $CDCl_3$) δ 7.48-7.41 (m, 4H, ArH), 7.37 (t, *J* = 7.2 Hz, 1H, ArH), 6.99 (d, *J* = 7.6 Hz, 1H, ArH), 6.80-6.77 (m, 3H, ArH), 6.71-6.69 (m, 2H, ArH), 6.30 (d, *J* = 8.0 Hz, 1H, ArH), 6.20 (t, *J* = 8.0 Hz, 1H, ArH), 5.82-5.79 (m, 2H, CH), 5.23 (s, 1H, CH), 5.04 (d, *J* = 16.0 Hz, 1H, CH_2), 4.57 (d, *J* = 5.6 Hz, 1H, CH), 4.42 (d, *J* = 16.0 Hz, 1H, CH_2), 4.15 (d, *J* = 9.2 Hz, 1H, CH), 3.94 (s, 3H, OCH_3), 3.78 (s, 3H, OCH_3), 3.28 (s, 3H, OCH_3), 2.48 (s, 1H, CH), 2.45 (s, 3H, CH_3) ppm. ^{13}C NMR (100 MHz, $CDCl_3$) δ 168.6, 166.1, 164.6, 149.9, 145.6, 144.8, 138.5, 131.3, 129.7, 129.1, 128.9, 128.1, 128.0, 127.9, 127.8, 127.1, 126.8, 125.0, 124.2, 120.0, 106.2, 104.3, 102.5, 61.2, 57.4, 55.9, 53.1, 51.8, 50.1, 44.7, 39.4, 17.9 ppm. IR (KBr) υ: 3742, 3065, 2952, 2823, 1923, 1767, 1618, 1516, 1322, 1207, 1107, 1023, 957, 834, 723 cm^{-1}; MS (*m*/*z*): HRMS (ESI) Calcd. for $C_{36}H_{33}BrNaN_2O_6$ ($[M+Na]^+$): 691.1420, found: 691.1415.

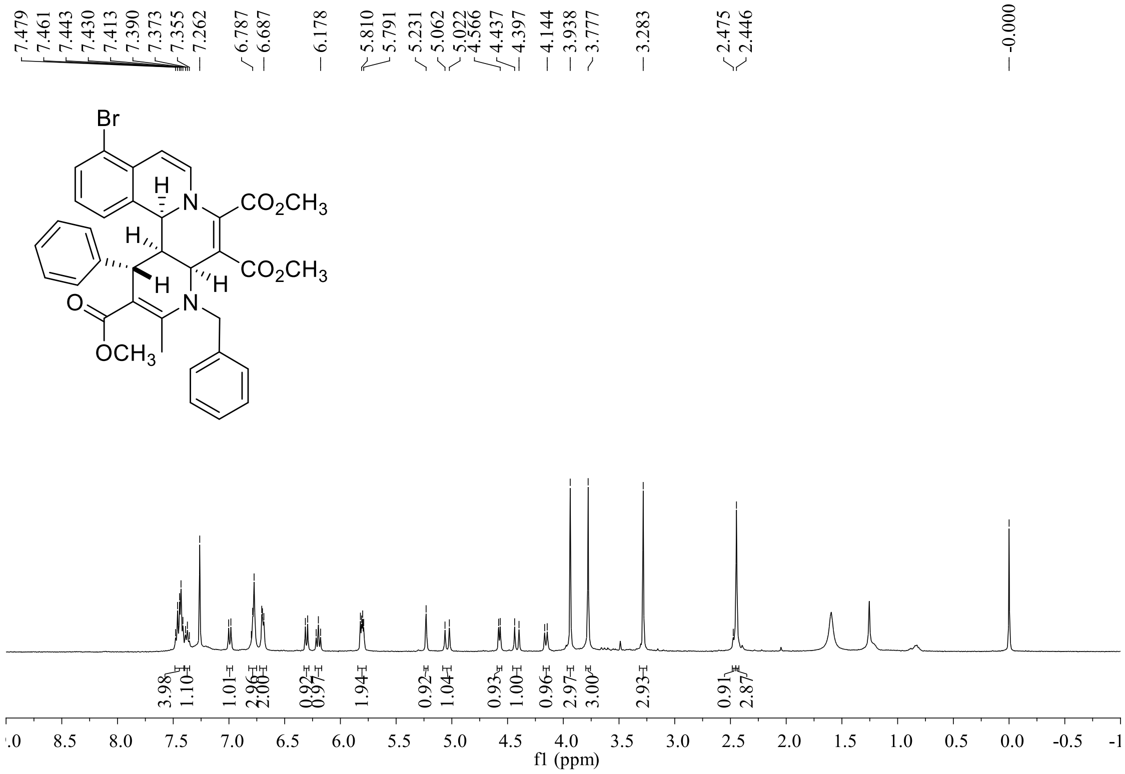

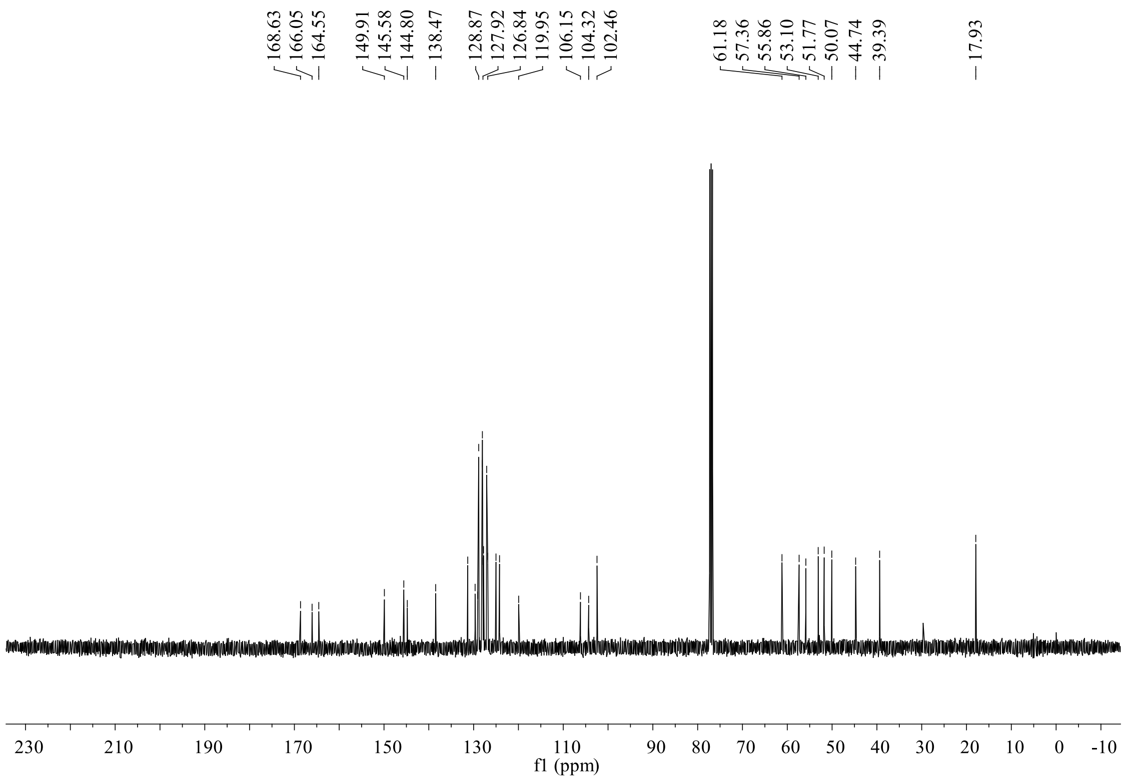
168.63
166.05
164.55
149.91
145.58
144.80
138.47
128.87
127.92
126.84
119.95
106.15
104.32
102.46
61.18
57.36
55.86
53.10
51.77
50.07
44.74
39.39
17.93
230 210 190 170 150 130 110 90 80 70 60 50 40 30 20 10 0 -10
f1 (ppm)

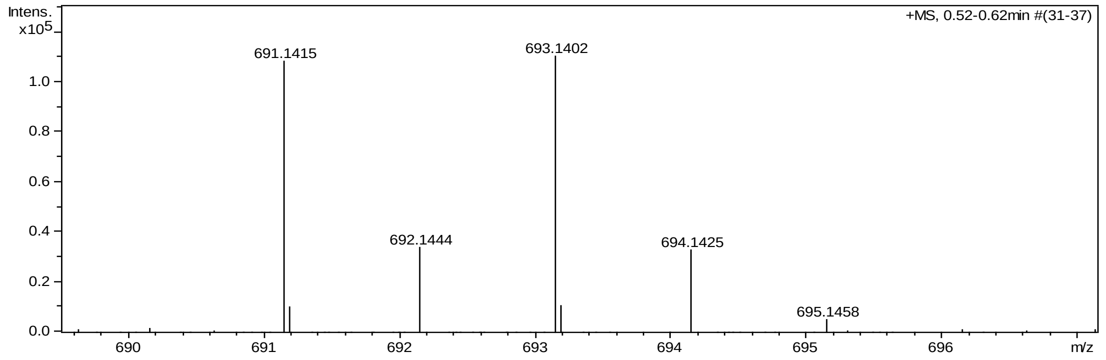
Intens.
x10^5
+MS, 0.52-0.62min #(31-37)
691.1415
693.1402
692.1444
694.1425
695.1458
1.0
0.8
0.6
0.4
0.2
0.0
690 691 692 693 694 695 696
m/z

Trimethyl 4-benzyl-11-bromo-3-methyl-1-phenyl-4,4a,13b,13c-tetrahydro-1*H*-isoquinolino[1,2-*f*][1,6]naphthyridine-2,5,6-tricarboxylate (4k): orange solid, 65%, m.p. 214-216 ºC; ^{1}H NMR (400 MHz, $CDCl_3$) δ 7.48-7.36 (m, 5H, ArH), 6.86-6.83 (m, 2H, ArH), 6.77 (d, *J* = 7.6 Hz, 2H, ArH), 6.64 (d, *J* = 7.6 Hz, 2H, ArH), 6.44 (d, *J* = 8.0 Hz, 1H, ArH), 6.24 (d, *J* = 8.0 Hz, 1H, ArH), 5.68 (d, *J* = 8.4 Hz, 1H, CH), 5.33 (d, *J* = 8.0 Hz, 1H, CH), 5.18 (s, 1H, CH), 5.04 (d, *J* = 16.0 Hz, 1H, CH_2), 4.57 (d, *J* = 5.6 Hz, 1H, CH), 4.42 (d, *J* = 16.0 Hz, 1H, CH_2), 4.12 (d, *J* = 9.2 Hz, 1H, CH), 3.93 (s, 3H, OCH_3), 3.78 (s, 3H, OCH_3), 3.29 (s, 3H, OCH_3), 2.49 (s, 1H, CH), 2.45 (s, 3H, CH_3) ppm. ^{13}C NMR (100 MHz, $CDCl_3$) δ 168.6, 166.1, 164.7, 150.1, 145.8, 145.1, 138.5, 131.5, 128.9, 128.7, 128.1, 128.0, 127.8, 127.4, 127.2, 127.0, 126.7, 126.4, 124.9, 121.1, 106.1, 103.7, 103.1, 61.0, 57.2, 55.9, 53.1, 51.8, 50.1, 44.5, 39.5, 17.9 ppm. IR (KBr) υ: 3744, 3547, 3389, 3015, 2940, 2847, 2357, 1968, 1688, 1581, 1438, 1366, 1241, 1099, 968, 877, 812 cm^{-1}; MS (*m*/*z*): HRMS (ESI) Calcd. for $C_{36}H_{33}BrNaN_2O_6$ ($[M+Na]^+$): 691.1420, found: 691.1410.

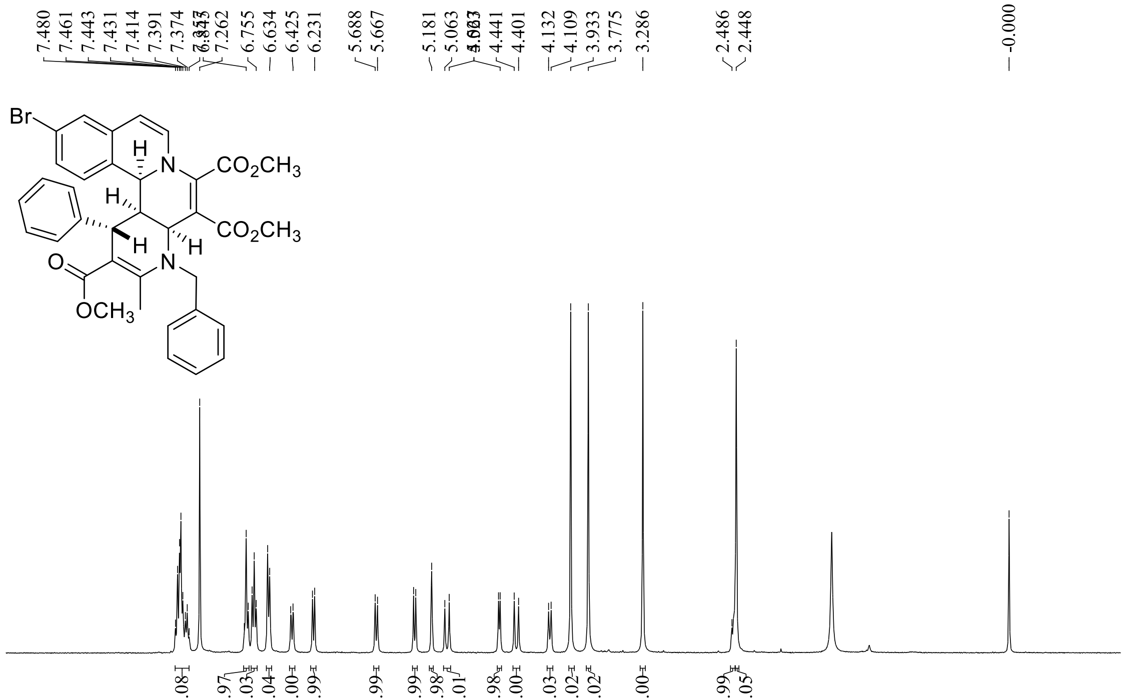

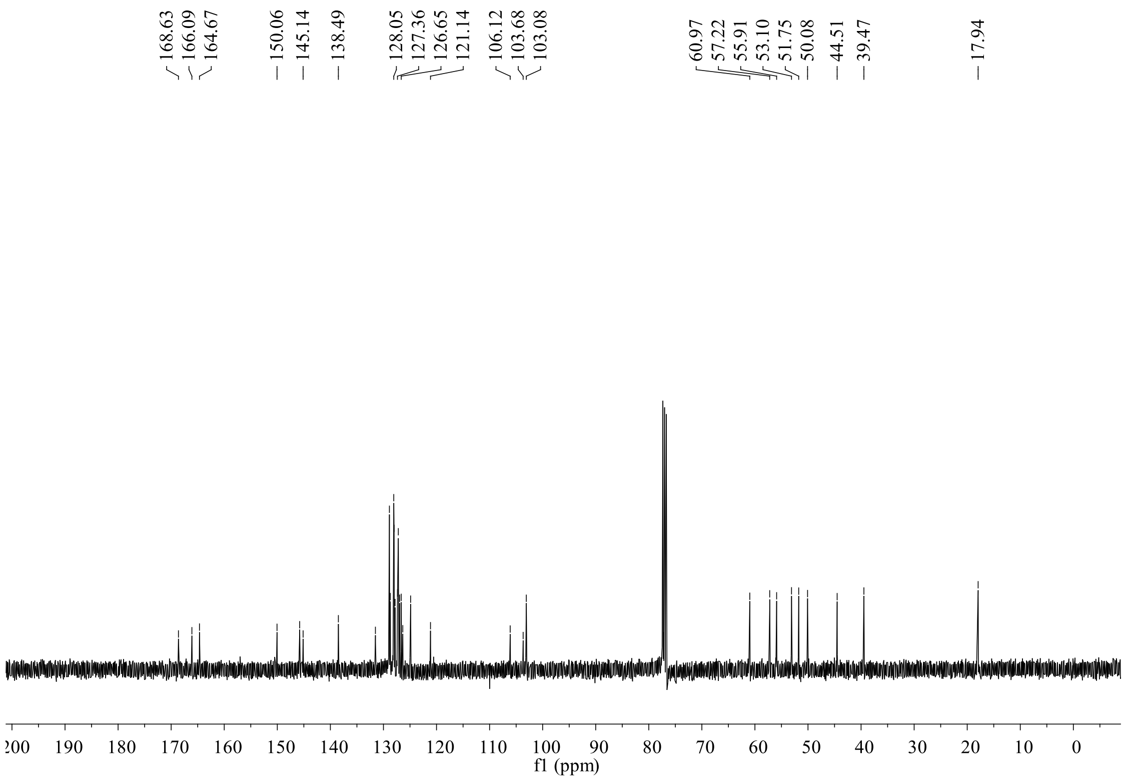
168.63
166.09
164.67
150.06
145.14
138.49
128.05
127.36
126.65
121.14
106.12
103.68
103.08
60.97
57.22
55.91
53.10
51.75
50.08
44.51
39.47
17.94
200 190 180 170 160 150 140 130 120 110 100 90 80 70 60 50 40 30 20 10 0
f1 (ppm)

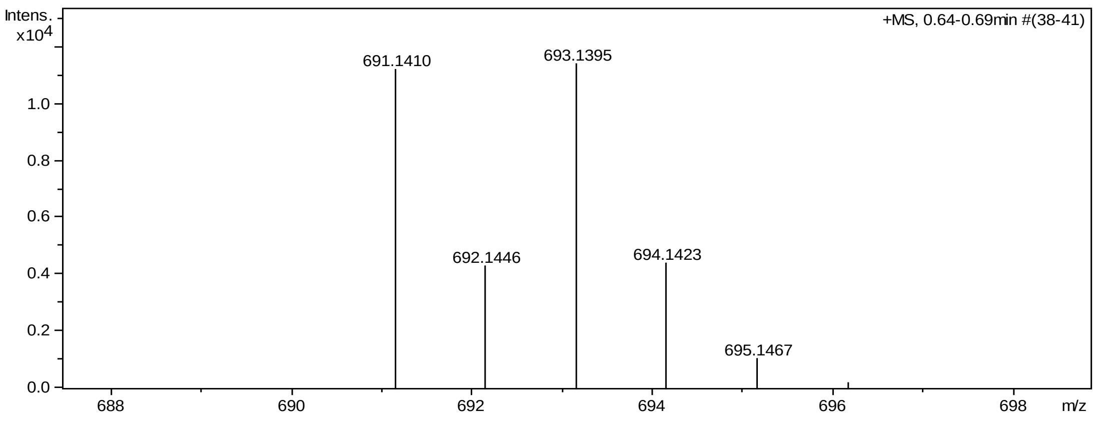
Intens.
x10^4
+MS, 0.64-0.69min #(38-41)
691.1410
693.1395
692.1446
694.1423
695.1467
1.0
0.8
0.6
0.4
0.2
0.0
688
690
692
694
696
698
m/z

2-Ethyl 5,6-dimethyl 4-benzyl-9-bromo-3-methyl-1-phenyl-4,4a,13b,13c-tetrahydro-1*H*-isoquinolino[1,2-*f*][1,6]naphthyridine-2,5,6-tricarboxylate (4l): orange solid, 74%, m.p. 185-187 ºC; ^{1}H NMR (400 MHz, $CDCl_3$) δ 7.46-7.37 (m, 5H, ArH), 7.19 (d, *J* = 7.2 Hz, 1H, ArH), 6.90 (t, *J* = 7.2 Hz, 1H, ArH), 6.74-6.72 (m, 3H, ArH), 6.64-6.60 (m, 3H, ArH), 6.46 (t, *J* = 7.6 Hz, 1H, CH), 5.89 (d, *J* = 7.2 Hz, 1H, CH), 5.27 (s, 1H, CH), 5.03 (d, *J* = 15.2 Hz, 1H, CH_2), 4.56 (d, *J* = 5.2 Hz, 1H, CH), 4.41 (d, *J* = 15.6 Hz, 1H, CH_2), 4.17 (d, *J* = 8.8 Hz, 1H, CH), 3.96 (s, 3H, OCH_3), 3.78 (s, 5H, OCH_3, OCH_2), 2.54 (t, *J* = 7.6 Hz, 1H, CH), 2.45 (s, 3H, CH_3), 0.85 (t, *J* = 7.2 Hz, 3H, CH_3) ppm. ^{13}C NMR (100 MHz, $CDCl_3$) δ 168.1, 166.1, 164.4, 150.1, 145.6, 144.0, 138.5, 128.9, 128.6, 128.1, 128.1, 127.8, 127.7, 127.6, 127.3, 127.1, 124.9, 124.7, 106.4, 105.0, 99.7, 61.6, 58.8, 57.3, 55.8, 53.3, 51.8, 44.8, 39.3, 17.9, 13.9 ppm. IR (KBr) υ: 3762, 3056, 2929, 2843, 1897, 1717, 1628, 1496, 1372, 1127, 1021, 927, 845, 729 cm^{-1}; MS (*m/z*): HRMS (ESI) Calcd. for $C_{37}H_{35}BrNaN_2O_6$ ($[M+Na]^+$): 705.1576, found: 705.1564.

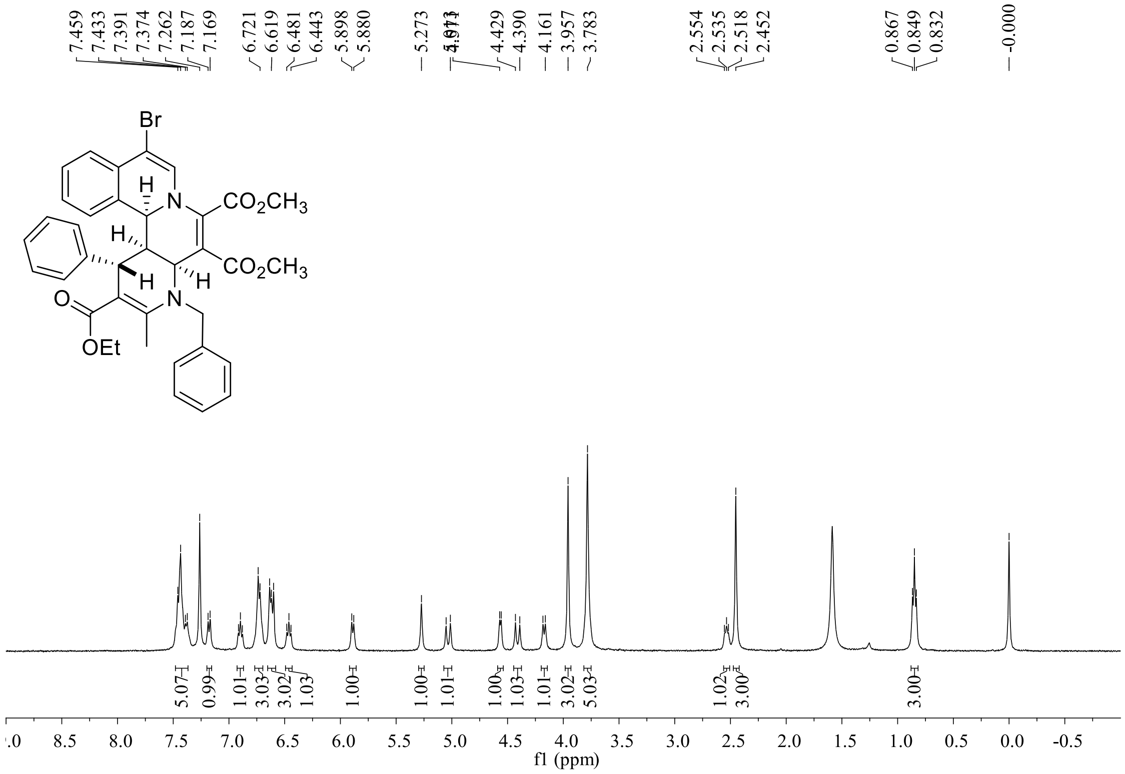

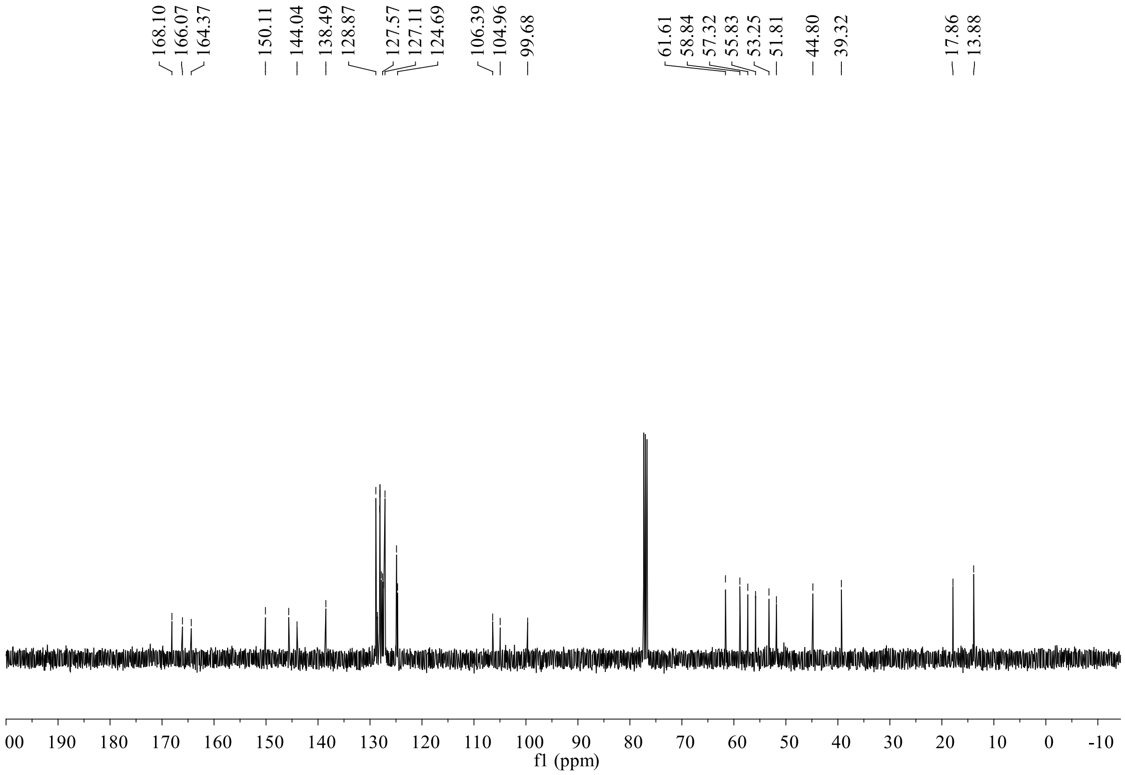
168.10
166.07
164.37
150.11
144.04
138.49
128.87
127.57
127.11
124.69
106.39
104.96
99.68
61.61
58.84
57.32
55.83
53.25
51.81
44.80
39.32
17.86
13.88
00 190 180 170 160 150 140 130 120 110 100 90 80 70 60 50 40 30 20 10 0 -10
f1 (ppm)

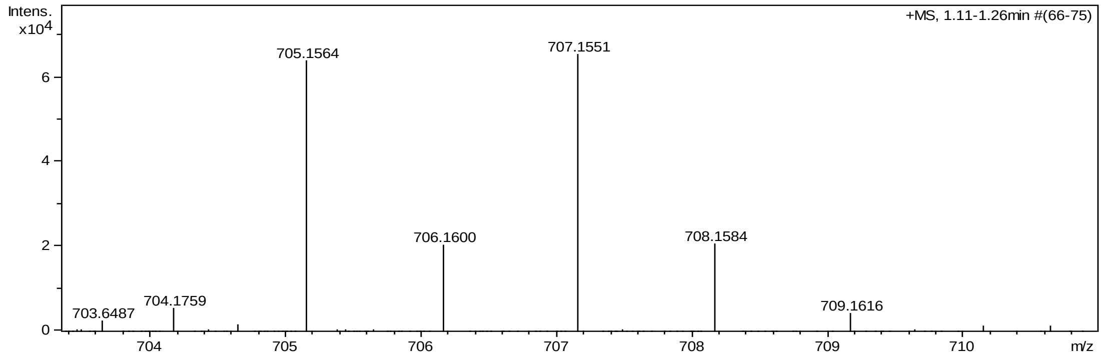
Intens.
x10^4
+MS, 1.11-1.26min #(66-75)
705.1564
707.1551
706.1600
708.1584
703.6487
704.1759
709.1616
6
4
2
0
704
705
706
707
708
709
710
m/z

Trimethyl 4-butyl-3-methyl-1-phenyl-4,4a,13b,13c-tetrahydro-1*H*-isoquinolino[1,2-*f*][1,6]naphthyridine-2,5,6-tricarboxylate (4m): orange solid, 77%, m.p. 205-207 ºC; ^{1}H NMR (400 MHz, $CDCl_3$) δ 6.88 (t, *J* = 7.6 Hz, 1H, ArH), 6.82-6.79 (m, 4H, ArH), 6.74-6.72 (m, 2H, ArH), 6.49 (t, *J* = 7.6 Hz, 1H, ArH), 6.28 (d, *J* = 8.0 Hz, 1H, ArH), 6.24 (d, *J* = 8.0 Hz, 1H, CH), 5.46 (d, *J* = 8.0 Hz, 1H, CH), 5.41 (s, 1H, CH), 4.55 (d, *J* = 6.0 Hz, 1H, CH), 4.22 (d, *J* = 8.0 Hz, 1H, CH), 3.92 (s, 3H, OCH_3), 3.89-3.81 (m, 1H, CH_2), 3.70 (s, 3H, OCH_3), 3.31 (s, 3H, OCH_3), 3.29-3.22 (m, 1H, CH_2), 3.00 (t, *J* = 6.8 Hz, 1H, CH), 2.35 (s, 3H, CH_3), 1.69-1.62 (m, 2H, CH_2), 1.50-1.41 (m, 2H, CH_2), 1.04 (t, *J* = 7.2 Hz, 3H, CH_3) ppm. ^{13}C NMR (100 MHz, $CDCl_3$) δ 168.8, 166.2, 165.0, 151.3, 147.1, 145.8, 129.6, 127.8, 127.6, 127.4, 127.2, 126.3, 125.4, 124.7, 124.7, 105.4, 104.5, 103.0, 61.5, 58.4, 53.0, 52.6, 51.5, 49.9, 47.4, 39.6, 32.5, 20.4, 17.0, 14.0 ppm. IR (KBr) υ: 3742, 3073, 2962, 2823, 1987, 1727, 1618, 1536, 1322, 1097, 1031, 957, 856, 731 cm^{-1}; MS (*m*/*z*): HRMS (ESI) Calcd. for $C_{33}H_{36}NaN_2O_6$ ($[M+Na]^+$): 579.2471, found: 579.2465.

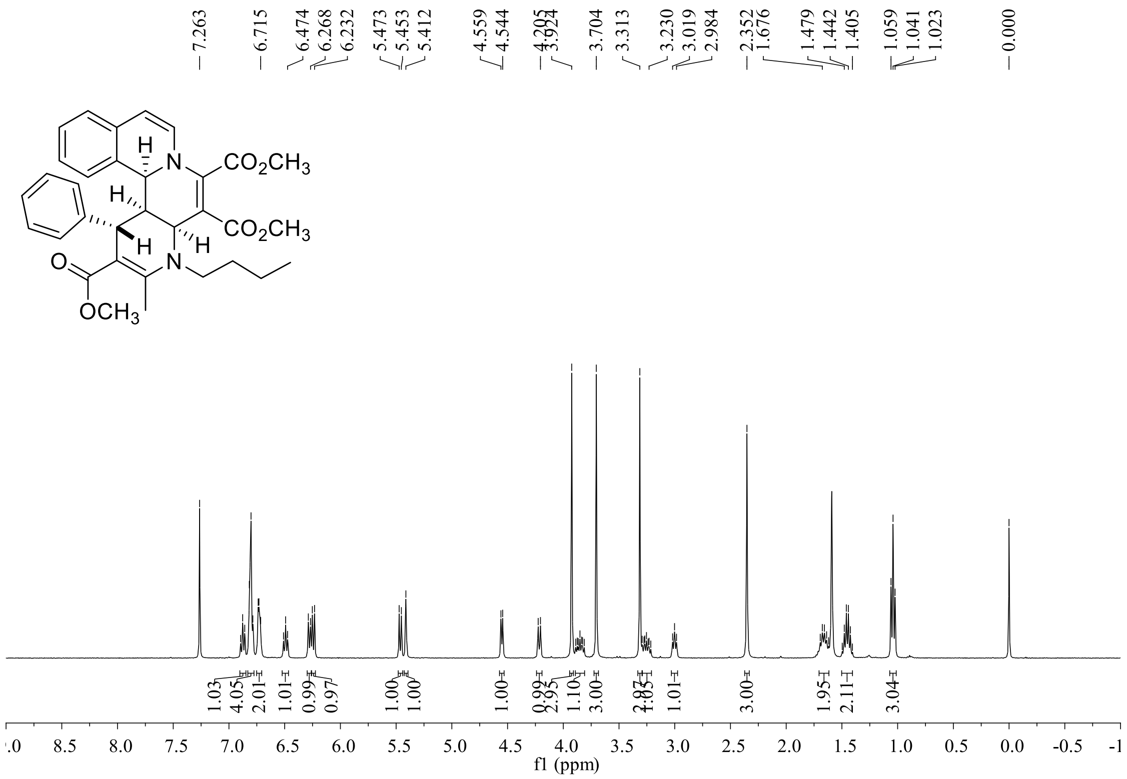

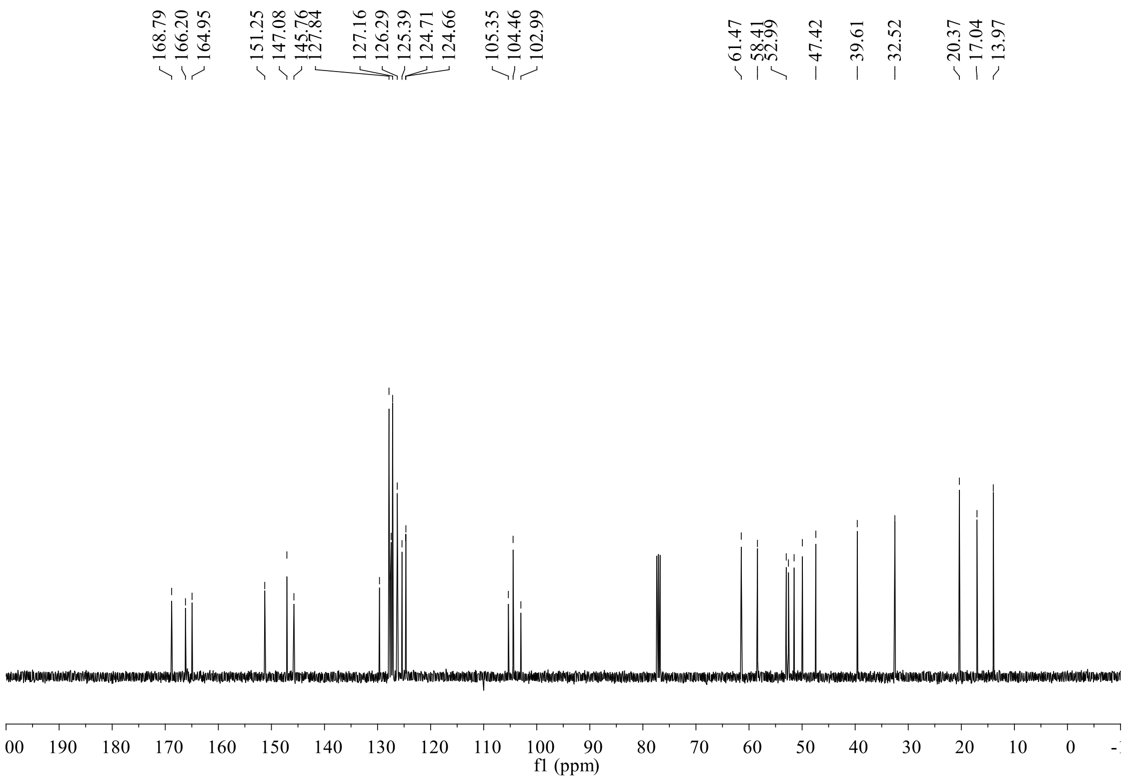
168.79
166.20
164.95
151.25
147.08
145.76
127.84
127.16
126.29
125.39
124.71
124.66
105.35
104.46
102.99
61.47
58.41
52.99
47.42
39.61
32.52
20.37
17.04
13.97
f1 (ppm)

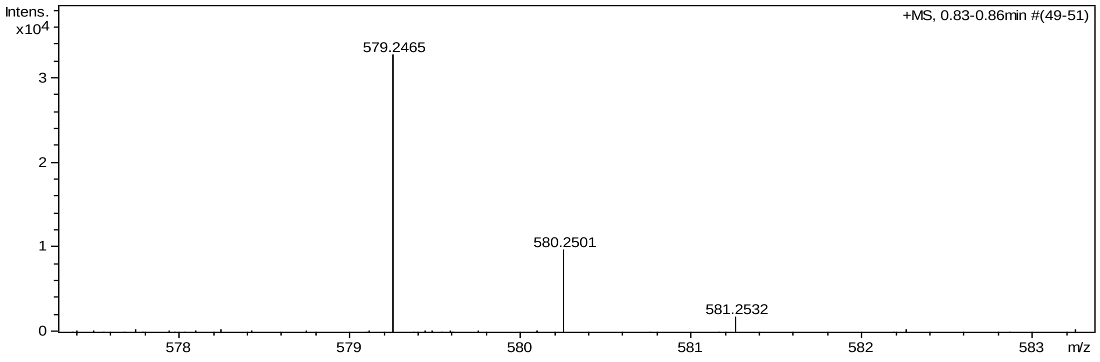
Intens.
x10^4
+MS, 0.83-0.86min #(49-51)
579.2465
580.2501
581.2532
m/z

Trimethyl 10-bromo-4-butyl-3-methyl-1-phenyl-4,4a,13b,13c-tetrahydro-1*H*-isoquinolino[1,2-*f*][1,6]naphthyridine-2,5,6-tricarboxylate (4n): orange solid, 78%, m.p. 193-195 ºC; ^{1}H NMR (400 MHz, CDCl$_3$) δ 7.06 (d, *J* = 8.0 Hz, 1H, ArH), 6.87-6.77 (m, 5H, ArH), 6.33-6.27 (m, 2H, ArH), 6.20 (d, *J* = 7.6 Hz, 1H, CH), 5.83 (d, *J* = 8.0 Hz, 1H, CH), 5.38 (s, 1H, CH), 4.53 (d, *J* = 6.0 Hz, 1H, CH), 4.21 (d, *J* = 8.4 Hz, 1H, CH), 3.92 (s, 3H, OCH$_3$), 3.87-3.79 (m, 1H, CH$_2$), 3.70 (s, 3H, OCH$_3$), 3.30 (s, 3H, OCH$_3$), 3.27-3.19 (m, 1H, CH$_2$), 2.91 (t, *J* = 7.2 Hz, 1H, CH), 2.34 (s, 3H, CH$_3$), 1.69-1.61 (m, 2H, CH$_2$), 1.49-1.40 (m, 2H, CH$_2$), 1.03 (t, *J* = 7.2 Hz, 3H, CH$_3$) ppm. ^{13}C NMR (100 MHz, CDCl$_3$) δ 168.7, 166.0, 164.6, 151.0, 146.6, 144.8, 131.5, 129.7, 129.4, 128.2, 128.0, 127.3, 126.8, 124.9, 124.5, 120.0, 110.0, 105.1, 104.6, 102.3, 61.3, 58.4, 53.1, 52.6, 51.6, 50.0, 47.4, 39.6, 32.4, 20.3, 17.1, 14.0 ppm. IR (KBr) υ: 3745, 3039, 2937, 2838, 1946, 1765, 1682, 1527, 1319, 1192, 1102, 1005, 947, 856, 752 cm^{-1}; MS (*m/z*): HRMS (ESI) Calcd. for $C_{33}H_{35}BrNaN_2O_6$ ([M+Na]$^+$): 657.1576, found: 657.1570.

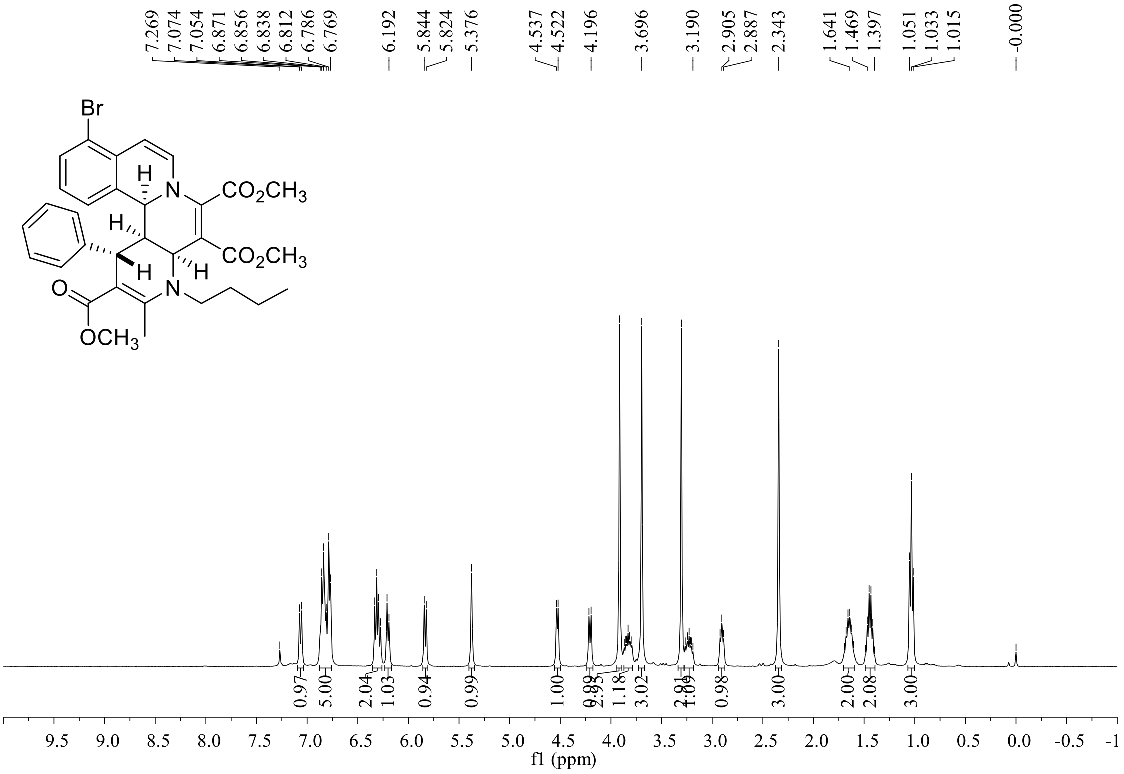

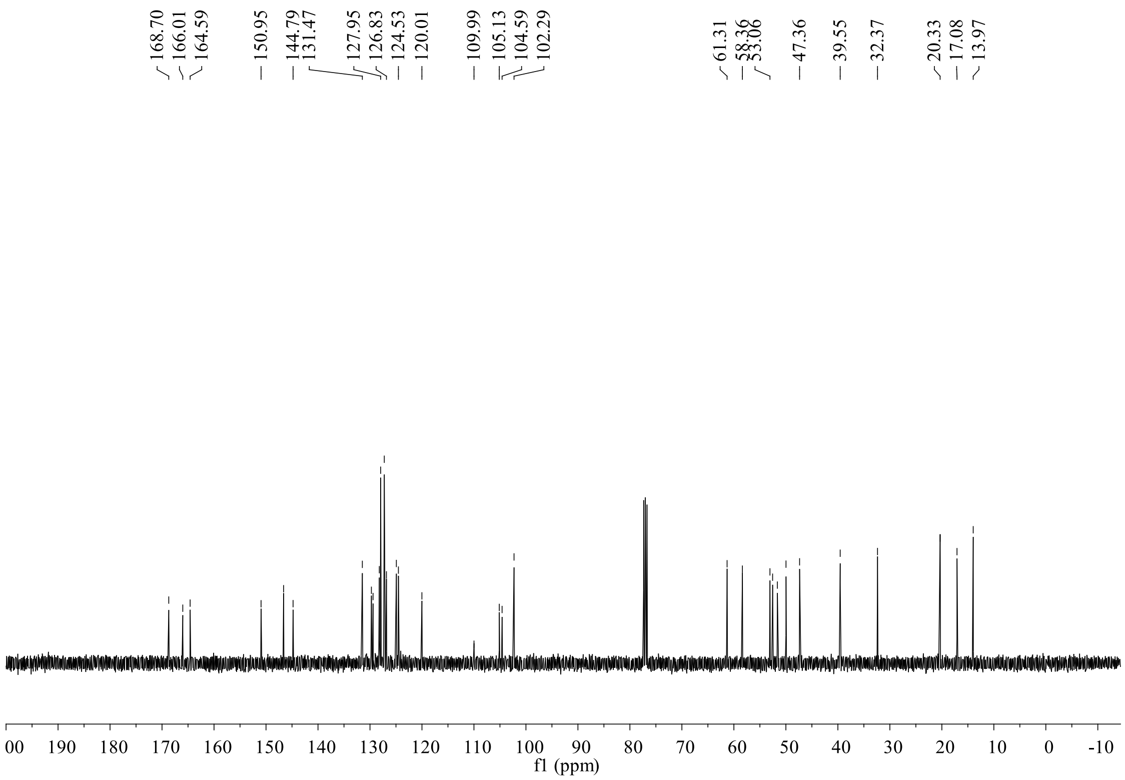
168.70
166.01
164.59
150.95
144.79
131.47
127.95
126.83
124.53
120.01
109.99
105.13
104.59
102.29
61.31
58.36
53.06
47.36
39.55
32.37
20.33
17.08
13.97
00 190 180 170 160 150 140 130 120 110 100 90 80 70 60 50 40 30 20 10 0 -10
f1 (ppm)

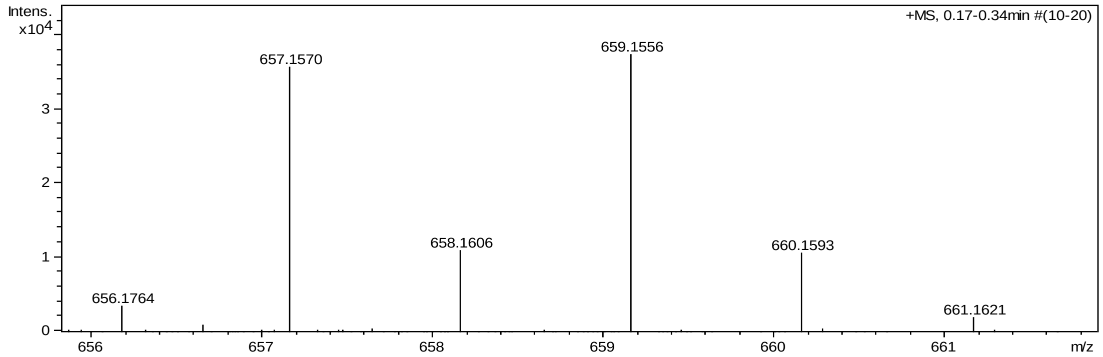
Intens.
x10^4
+MS, 0.17-0.34min #(10-20)
657.1570
659.1556
658.1606
660.1593
656.1764
661.1621
656
657
658
659
660
661
m/z

Dimethyl 2-acetyl-4-benzyl-3-methyl-1-phenyl-4,4a,13b,13c-tetrahydro-1*H*-isoquinolino[1,2-*f*][1,6]naphthyridine-5,6-dicarboxylate (4o): orange solid, 65%, m.p. 171-173 °C; ^{1}H NMR (400 MHz, $CDCl_3$) δ 7.42-7.34 (m, 5H, ArH), 6.82-6.76 (m, 4H, ArH), 6.60 (d, *J* = 7.2 Hz, 2H, ArH), 6.51 (t, *J* = 7.6 Hz, 1H, ArH), 6.25 (d, *J* = 7.6 Hz, 1H, ArH), 6.03 (d, *J* = 7.6 Hz, 1H, CH), 5.48 (d, *J* = 7.6 Hz, 1H, CH), 5.27 (s, 1H, CH), 5.03 (d, *J* = 15.6 Hz, 1H, CH_2), 4.58 (d, *J* = 6.0 Hz, 1H, CH), 4.48 (d, *J* = 15.6 Hz, 1H, CH_2), 4.22 (d, *J* = 7.6 Hz, 1H, CH), 3.94 (s, 3H, OCH_3), 3.77 (s, 3H, OCH_3), 2.69 (t, *J* = 6.8 Hz, 1H, CH), 2.40 (s, 3H, CH_3), 1.93 (s, 3H, CH_3) ppm. ^{13}C NMR (100 MHz, $CDCl_3$) δ 198.1, 166.0, 164.8, 150.4, 146.1, 144.9, 138.4, 129.5, 128.8, 128.2, 128.1, 127.8, 127.6, 126.6, 126.0, 125.3, 124.9, 116.2, 104.8, 103.0, 61.4, 57.6, 56.0, 53.1, 51.7, 47.4, 40.4, 30.3, 18.6 ppm. IR (KBr) υ: 3621, 3043, 2896, 2831, 2332, 1934, 1723, 1623, 1524, 1245, 1123, 943, 841, 721 cm^{-1}; MS (*m*/*z*): HRMS (ESI) Calcd. for $C_{36}H_{35}N_2O_5$ ($[M+H]^+$): 575.2546, found: 575.2549.

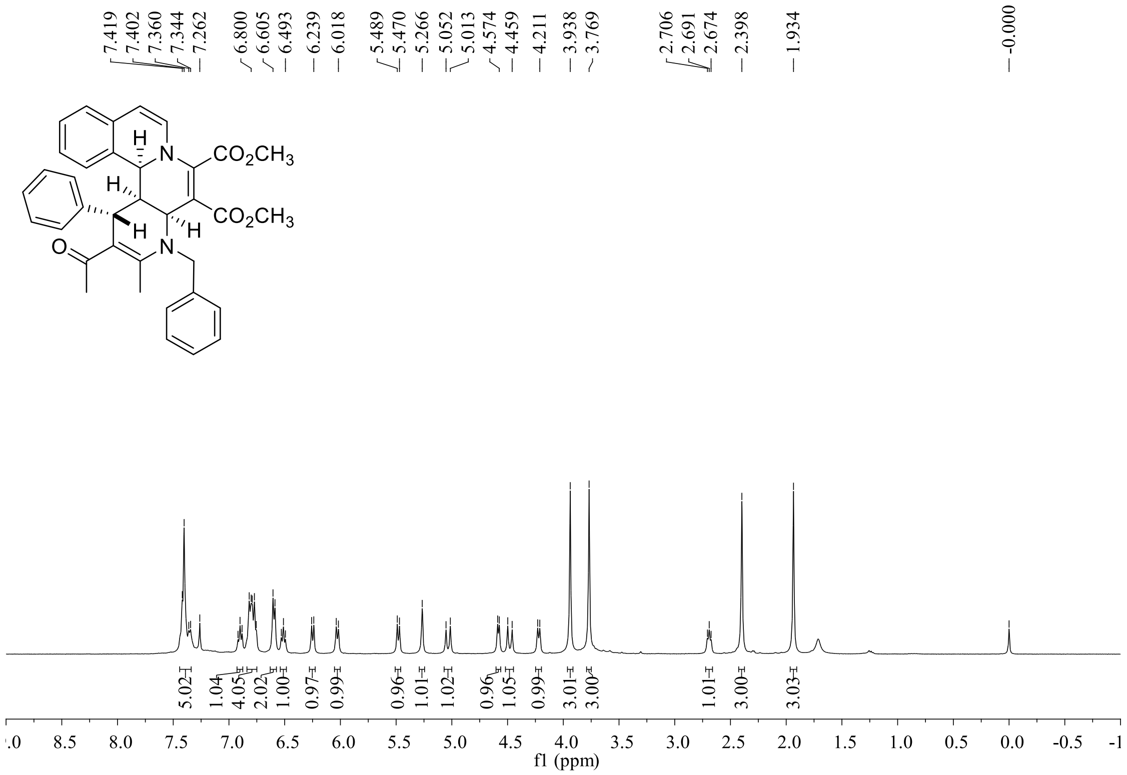

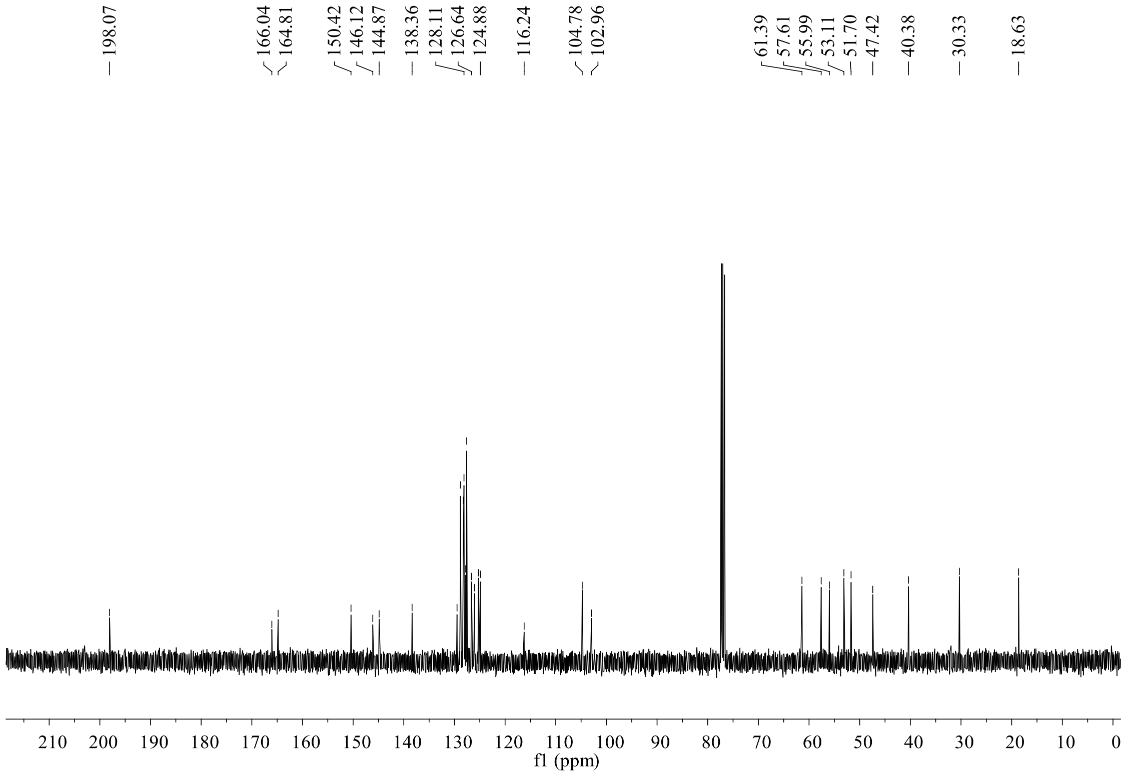
198.07
166.04
164.81
150.42
146.12
144.87
138.36
128.11
126.64
124.88
116.24
104.78
102.96
61.39
57.61
55.99
53.11
51.70
47.42
40.38
30.33
18.63
210 200 190 180 170 160 150 140 130 120 110 100 90 80 70 60 50 40 30 20 10 0
f1 (ppm)

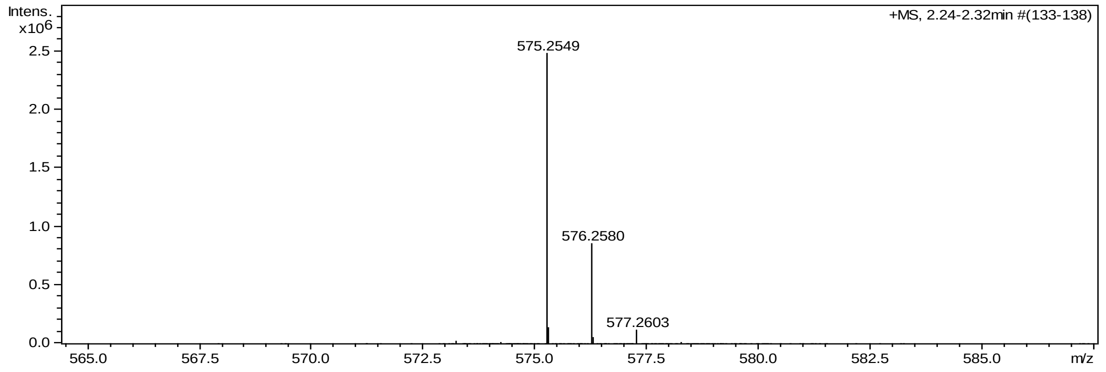
Intens.
x10^6
2.5
2.0
1.5
1.0
0.5
0.0
+MS, 2.24-2.32min #(133-138)
575.2549
576.2580
577.2603
565.0 567.5 570.0 572.5 575.0 577.5 580.0 582.5 585.0 m/z

Trimethyl 2-benzyl-3-methyl-5-phenyl-2-azabicyclo[4.2.0]octa-3,7-diene-4,7,8-tricarboxylate (5a): yellow solid, 89%, m.p. 118-120 °C; ^{1}H NMR (400 MHz, $CDCl_3$) δ 7.26-7.25 (m, 7H, ArH), 7.22-7.20 (m, 1H, ArH), 7.12-7.10 (m, 2H, ArH), 4.83 (d, J = 15.6 Hz, 1H, CH_2), 4.50 (d, J = 1.2 Hz, 1H, CH), 4.47 (d, J = 15.6 Hz, 1H, CH_2), 4.29 (d, J = 5.2 Hz, 1H, CH), 3.84 (s, 6H, OCH_3), 3.81-3.80 (m, 1H, CH), 3.62 (s, 3H, OCH_3), 2.46 (s, 3H, CH_3) ppm. ^{13}C NMR (100 MHz, $CDCl_3$) δ 169.7, 161.5, 161.3, 155.7, 145.4, 143.7, 137.5, 135.8, 128.6, 128.2, 127.9, 127.7, 127.5, 126.1, 102.3, 57.1, 53.8, 52.8, 52.2, 52.1, 50.9, 38.6, 17.3 ppm. IR (KBr) υ: 3736, 3019, 2943, 2835, 2357, 1966, 1730, 1659, 1546, 1229, 1105, 956, 847, 772 cm^{-1}; MS (m/z): HRMS (ESI) Calcd. for $C_{27}H_{27}NaNO_6$ ($[M+Na]^+$): 484.1736, found: 484.1739.

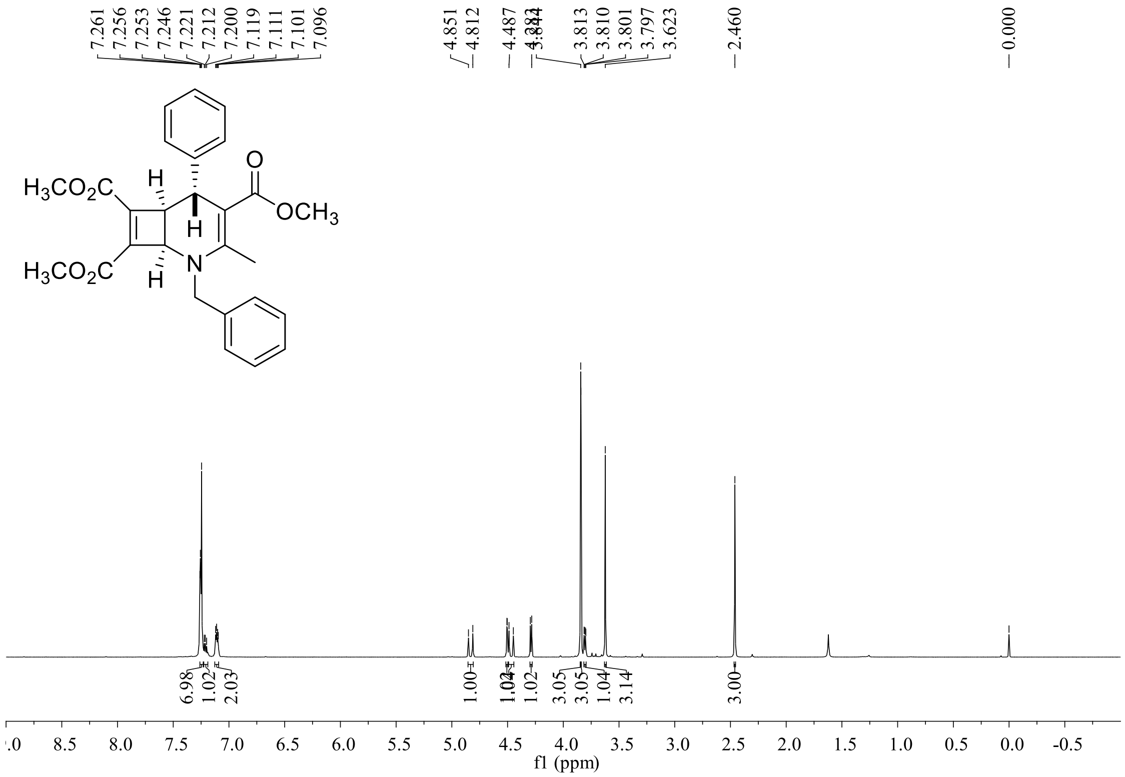

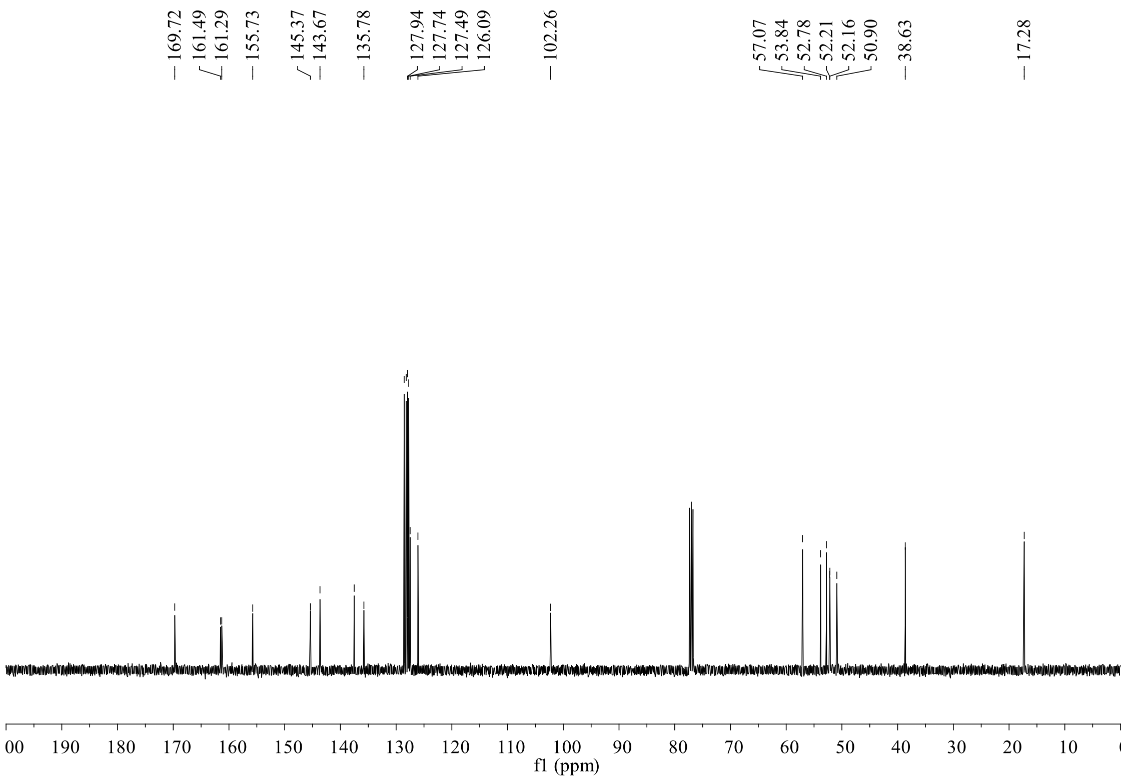

169.72
161.49
161.29
155.73
145.37
143.67
135.78
127.94
127.74
127.49
126.09
102.26
57.07
53.84
52.78
52.21
52.16
50.90
38.63
17.28
190
180
170
160
150
140
130
120
110
100
90
80
70
60
50
40
30
20
10
f1 (ppm)

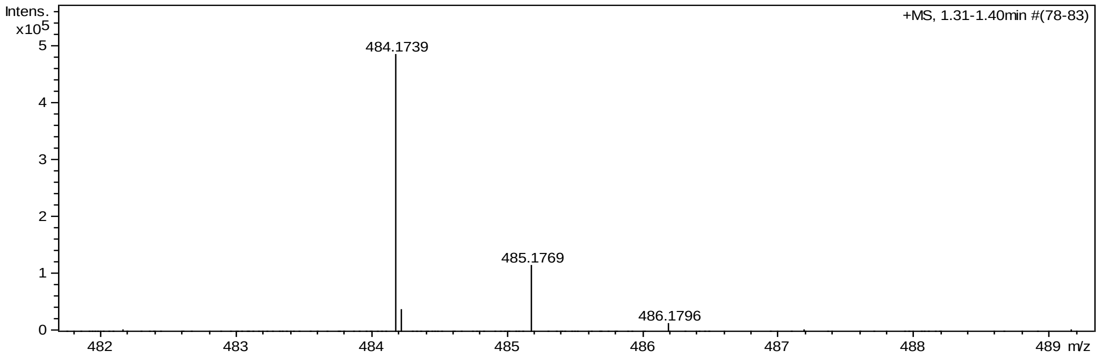

Intens.
x10^5
+MS, 1.31-1.40min #(78-83)
484.1739
485.1769
486.1796
482
483
484
485
486
487
488
489 m/z

7,8-Diethyl 4-methyl 2-benzyl-3-methyl-5-phenyl-2-azabicyclo[4.2.0]octa-3,7-diene-4,7,8-tricarboxylate (5b): yellow oily, 70%; ^{1}H NMR (400 MHz, $CDCl_3$) δ 7.25-7.21 (m, 8H, ArH), 7.11-7.10 (m, 2H, ArH), 4.84 (d, *J* = 15.6 Hz, 1H, CH_2), 4.53 (s, 1H, CH), 4.48 (d, *J* = 15.6 Hz, 1H, CH_2), 4.35-4.23 (m, 1H, CH), 4.31-4.24 (m, 4H, OCH_2), 3.79 (s, 1H, CH), 3.62 (s, 3H, OCH_3), 2.47 (s, 3H, CH_3), 1.39-1.33 (m, 6H, CH_3) ppm. ^{13}C NMR (100 MHz, $CDCl_3$) δ 169.8, 161.1, 161.0, 155.9, 145.6, 143.8, 137.6, 135.7, 128.5, 128.2, 127.9, 127.8, 127.5, 126.1, 102.1, 61.3, 61.2, 57.1, 53.9, 52.6, 50.9, 38.6, 17.2, 14.2, 14.1 ppm. IR (KBr) υ: 3745, 3034, 2945, 2815, 2347, 1967, 1732, 1643, 1528, 1426, 1321, 1230, 1108, 857, 772, 718 cm^{-1}; MS (*m/z*): HRMS (ESI) Calcd. for $C_{29}H_{31}NaNO_6$ ($[M+Na]^+$): 512.2049, found: 512.2050.

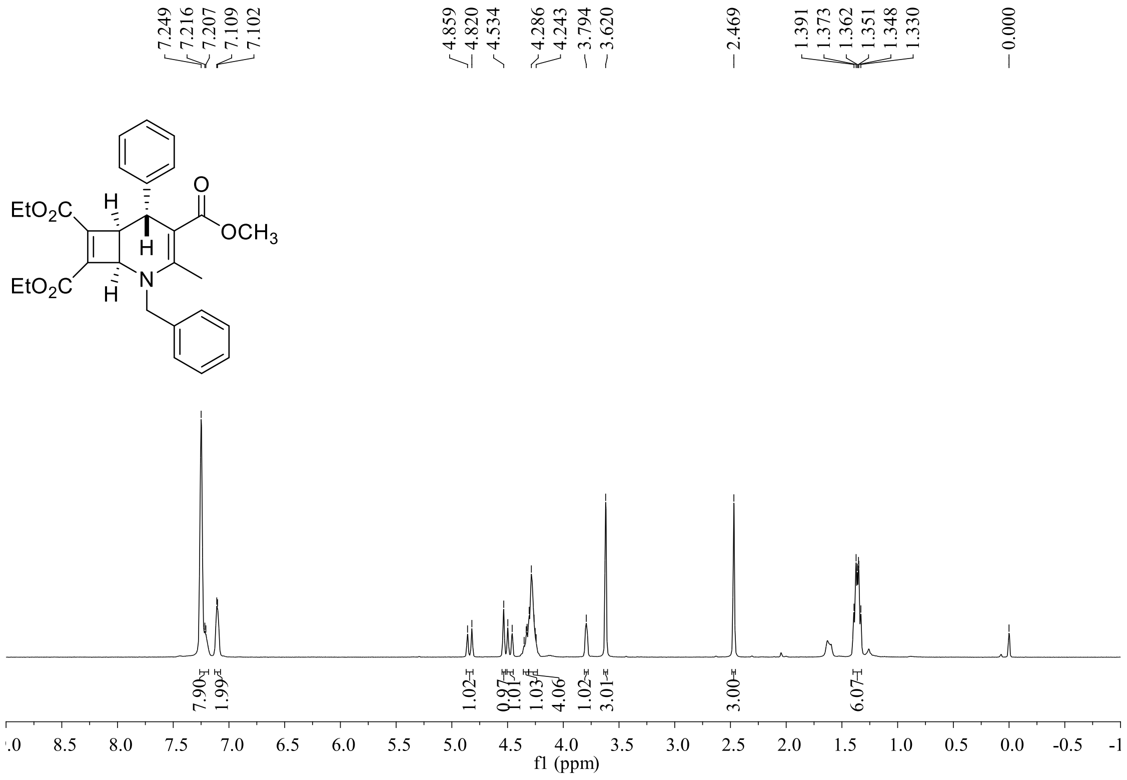

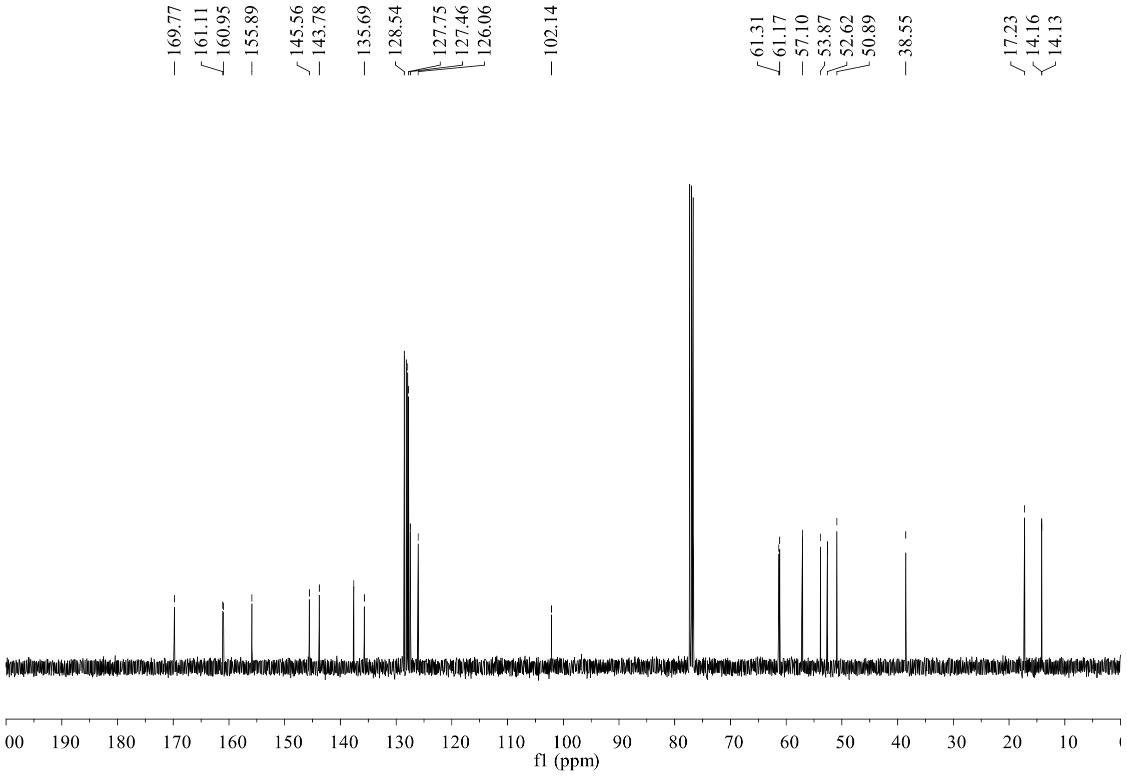
169.77
161.11
160.95
155.89
145.56
143.78
135.69
128.54
127.75
127.46
126.06
102.14
61.31
61.17
57.10
53.87
52.62
50.89
38.55
17.23
14.16
14.13
f1 (ppm)

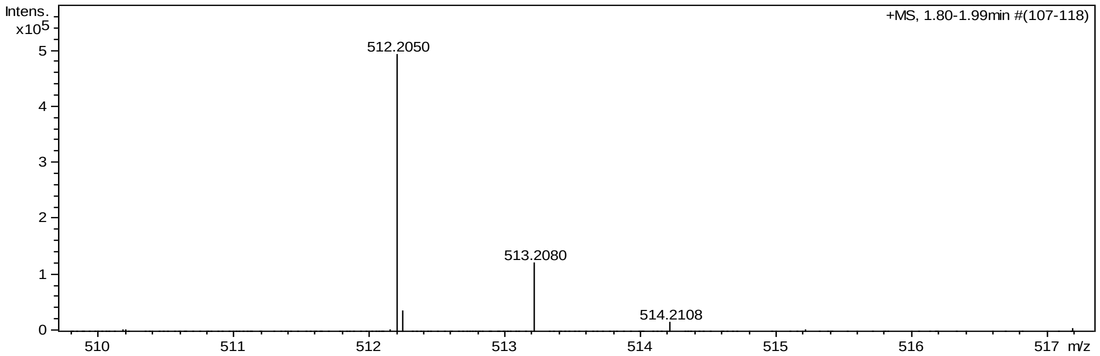
Intens.
x10^5
+MS, 1.80-1.99min #(107-118)
512.2050
513.2080
514.2108
m/z

Trimethyl 2-benzyl-5-(2-methoxyphenyl)-3-methyl-2-azabicyclo[4.2.0]octa-3,7-diene-4,7,8-tricarboxylate (5c): yellow solid, 80%, m.p. 148-150 °C; ^{1}H NMR (400 MHz, $CDCl_3$) δ 7.26-7.25 (m, 1H, ArH), 7.25-7.23 (m, 2H, ArH), 7.23-7.21 (m, 1H, ArH), 7.21-7.16 (m, 2H, ArH), 7.06 (d, *J* = 7.6 Hz, 1H, ArH), 6.89-6.83 (m, 2H, ArH), 4.83 (d, *J* = 15.6 Hz, 1H, CH_2), 4.74 (s, 1H, CH), 4.49 (d, *J* = 16.0 Hz, 1H, CH_2), 4.14 (d, *J* = 5.2 Hz, 1H, CH), 3.87 (s, 6H, OCH_3), 3.82 (s, 3H, OCH_3), 3.75 (d, *J* = 4.8 Hz, 1H, CH), 3.54 (s, 3H, OCH_3), 2.52 (s, 3H, CH_3) ppm. ^{13}C NMR (100 MHz, $CDCl_3$) δ 169.9, 161.6, 161.6, 157.5, 156.1, 146.7, 138.0, 135.7, 130.1, 128.5, 127.8, 127.7, 127.5, 127.4, 119.9, 110.5, 100.9, 57.5, 55.4, 54.0, 52.1, 52.1, 51.3, 50.9, 34.2, 17.2 ppm. IR (KBr) υ: 3746, 3028, 2947, 2834, 1737, 1686, 1569, 1434, 1312, 1233, 1127, 973, 754, 701 cm^{-1}; MS (*m*/*z*): HRMS (ESI) Calcd. for $C_{28}H_{30}NO_7$ ($[M+H]^+$): 492.2017, found: 492.2029.

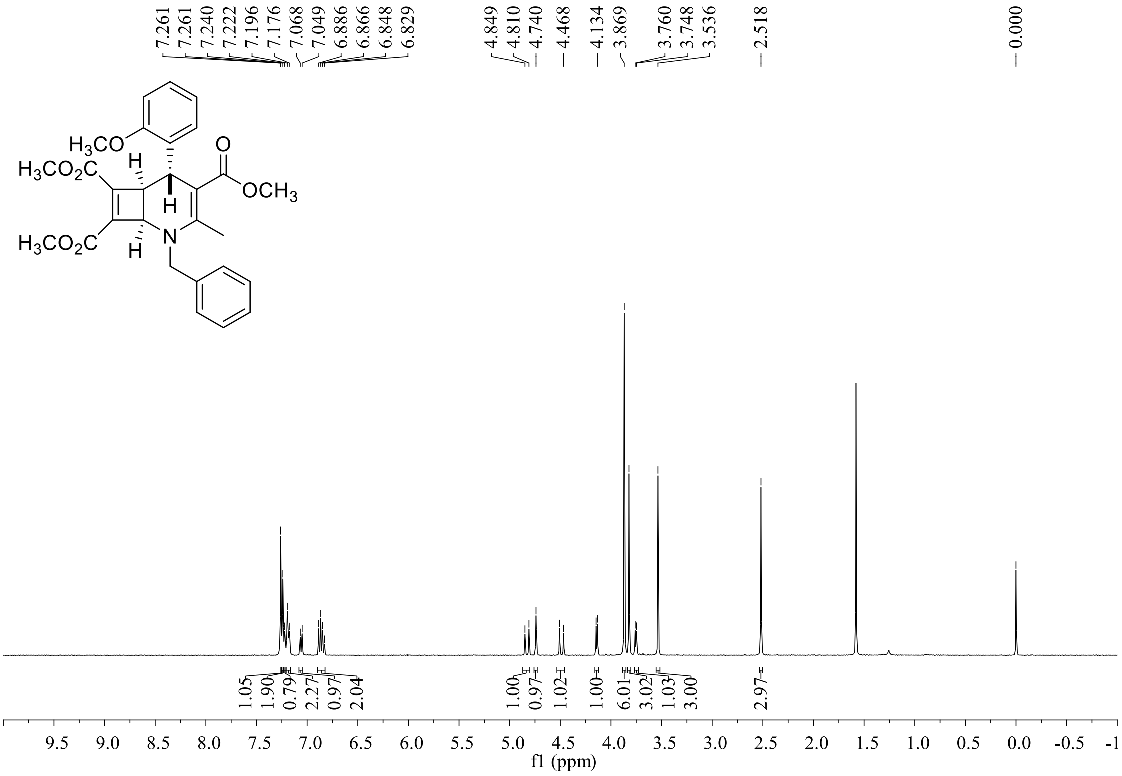

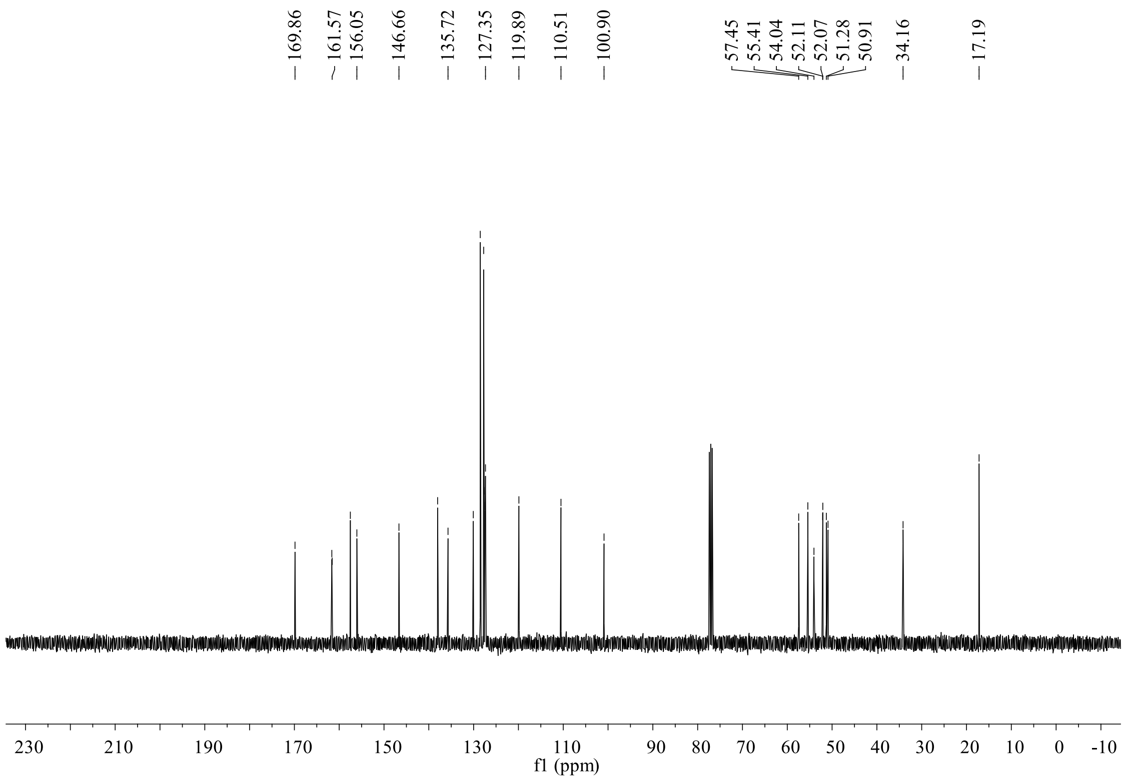
169.86
161.57
156.05
146.66
135.72
127.35
119.89
110.51
100.90
57.45
55.41
54.04
52.11
52.07
51.28
50.91
34.16
17.19
230 210 190 170 150 130 110 90 80 70 60 50 40 30 20 10 0 -10
f1 (ppm)

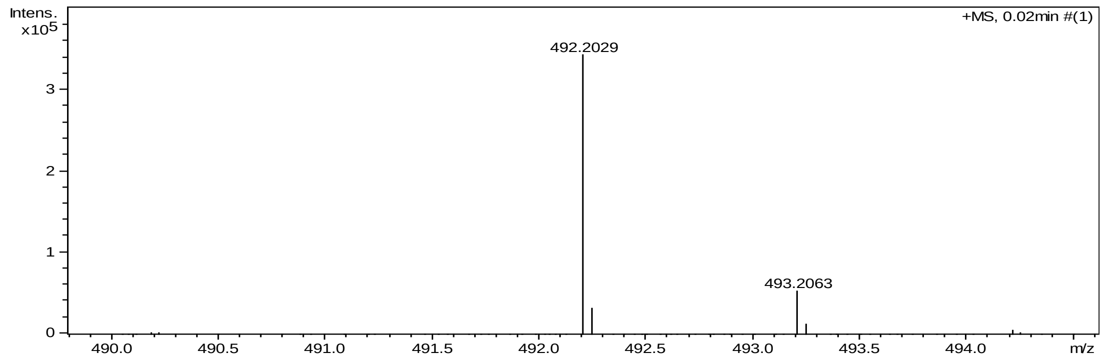
Intens.
x10^5
+MS, 0.02min #(1)
492.2029
493.2063
3
2
1
0
490.0 490.5 491.0 491.5 492.0 492.5 493.0 493.5 494.0 m/z

7,8-Diethyl 4-methyl 2-benzyl-5-(2-methoxyphenyl)-3-methyl-2-azabicyclo[4.2.0]octa-3,7-diene-4,7,8-tricarboxylate (5d): yellow oily, 65%; ^{1}H NMR (400 MHz, $CDCl_3$) δ 7.25-7.20 (m, 4H, ArH), 7.20-7.17 (m, 2H, ArH), 7.07 (dd, J_1 = 7.6 Hz, J_2 = 1,2 Hz, 1H, ArH), 6.90-6.82 (m, 2H, ArH), 4.84 (d, J = 15.6 Hz, 1H, CH_2), 4.79 (s, 1H, CH), 4.50 (d, J = 16.0 Hz, 1H, CH_2), 4.35-4.29 (m, 2H, CH_2), 4.29-4.23 (m, 2H, CH_2), 4.14 (d, J = 5.2 Hz, 1H, CH), 3.86 (s, 3H, OCH_3), 3.75 (dd, J_1 = 5.2 Hz, J_2 = 1,6 Hz, 1H, CH), 3.53 (s, 3H, OCH_3), 2.53 (s, 3H, CH_3), 1.37 (t, J = 7.2 Hz, 3H, CH_3), 1.36 (t, J = 7.2 Hz, 3H, CH_3) ppm. ^{13}C NMR (100 MHz, $CDCl_3$) δ 169.9, 161.3, 161.2, 157.5, 156.3, 146.9, 138.1, 135.6, 130.1, 128.5, 127.8, 127.8, 127.4, 127.3, 119.9, 110.5, 100.9, 61.2, 61.0, 57.5, 55.4, 54.1, 51.1, 50.9, 34.1, 17.2, 14.2, 14.2 ppm. IR (KBr) υ: 3726, 3433, 2949, 1719, 1685, 1561, 1435, 1368, 1234, 1125, 1100, 1054, 971, 755 cm^{-1}; MS (m/z): HRMS (ESI) Calcd. for $C_{30}H_{34}NO_7$ ($[M+H]^+$): 520.2330, found: 520.2335.

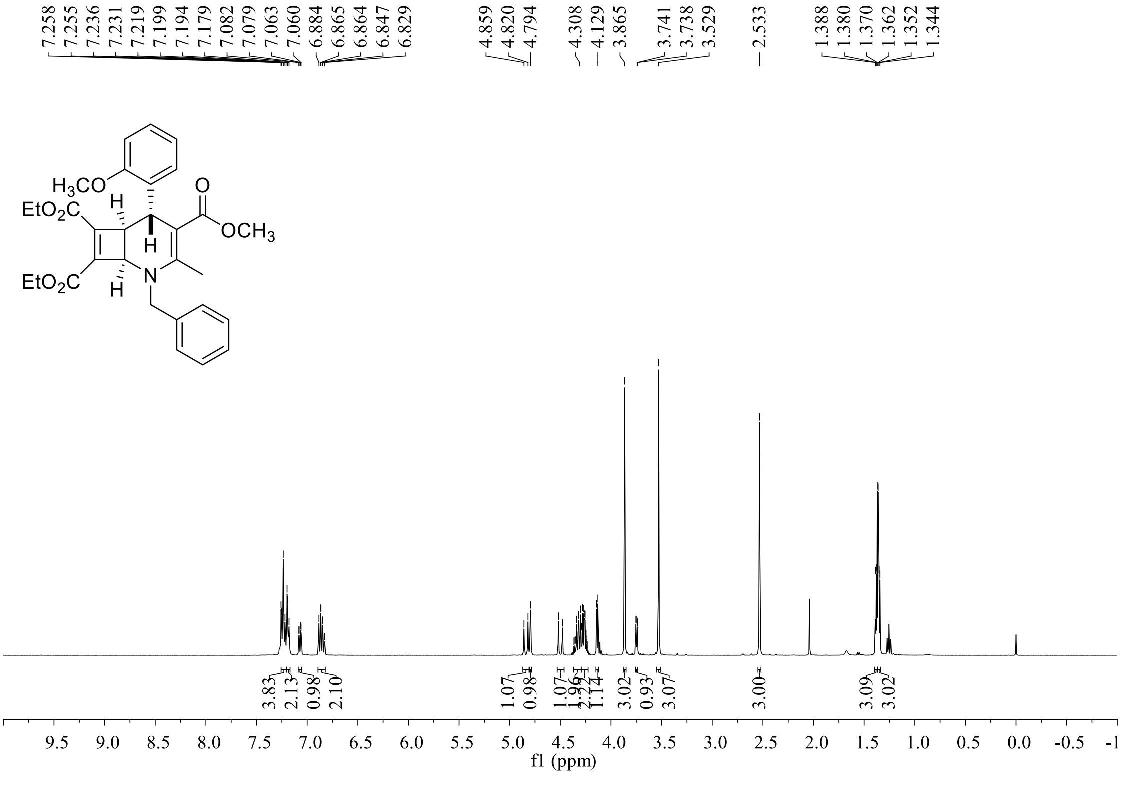

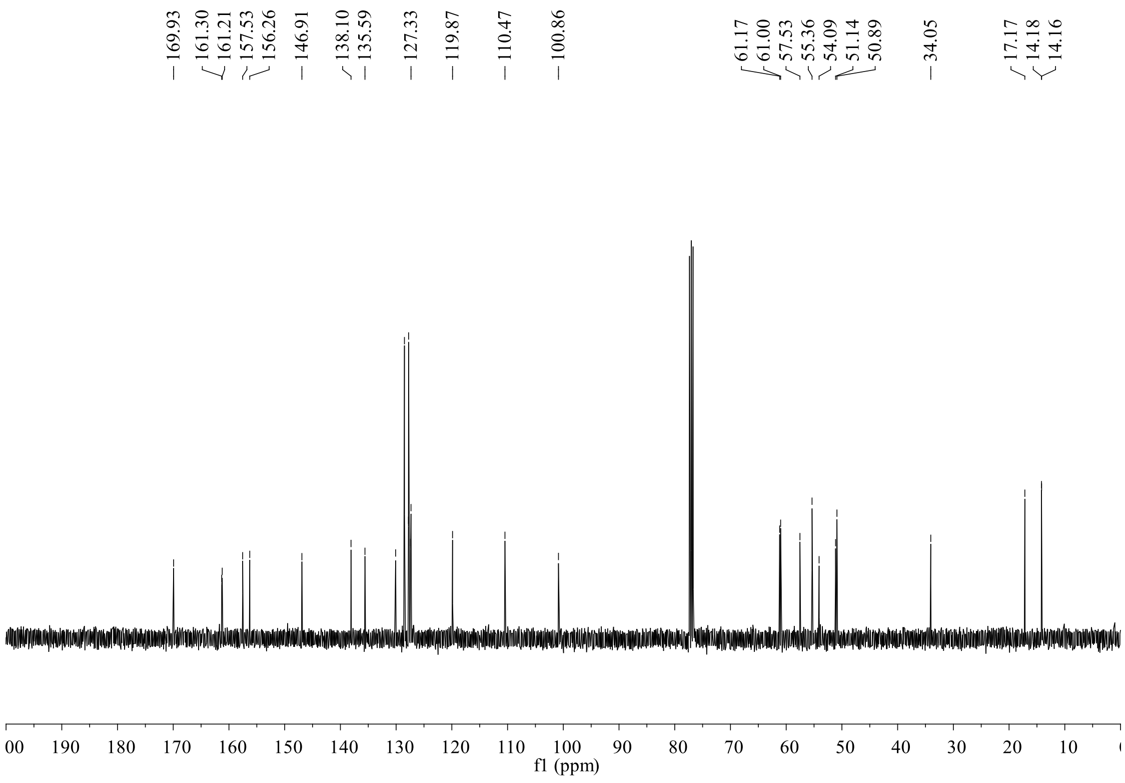
169.93
161.30
161.21
157.53
156.26
146.91
138.10
135.59
127.33
119.87
110.47
100.86
61.17
61.00
57.53
55.36
54.09
51.14
50.89
34.05
17.17
14.18
14.16
f1 (ppm)

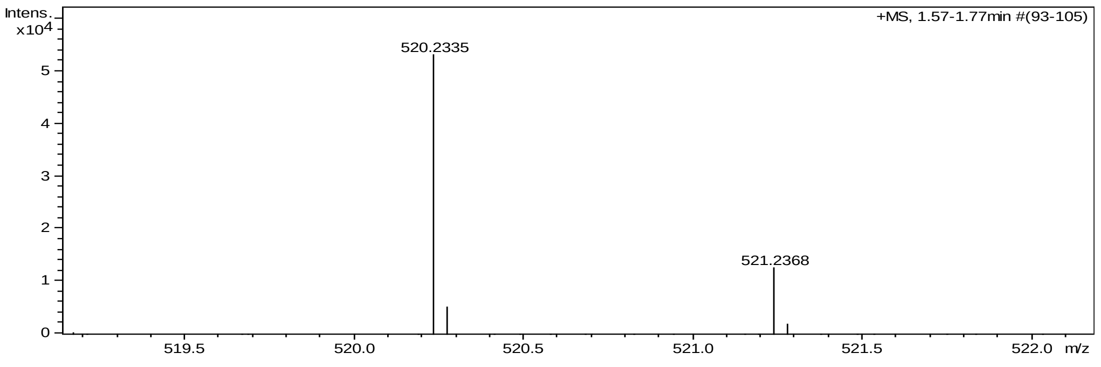
Intens.
x10^4
+MS, 1.57-1.77min #(93-105)
520.2335
521.2368
m/z

7,8-Diethyl 4-methyl 2-benzyl-3-methyl-5-(4-nitrophenyl)-2-azabicyclo[4.2.0]octa-3,7-diene-4,7,8-tricarboxylate (5e): yellow solid, 36%, m.p. 161-163 ºC; ^{1}H NMR (400 MHz, $CDCl_3$) δ 8.07-8.05 (m, 2H, ArH), 7.37-7.34 (m, 2H, ArH), 7.33-7.29 (m, 3H, ArH), 7.13-7.10 (m, 2H, ArH) 4.86 (d, J = 15.2 Hz, 1H, CH_2), 4.59 (s, 1H, CH), 4.48 (d, J = 15.2 Hz, 1H, CH_2), 4.37-4.34 (m, 1H, CH), 4.34-4.31 (m, 2H, CH_2), 4.31-4.26 (m, 2H, CH_2), 3.71 (d, J = 4.4 Hz, 1H, CH), 3.63 (s, 3H, OCH_3), 2.51 (s, 3H, CH_3), 1.40-1.37 (m, 3H, CH_3), 1.37-1.33 (m, 3H, CH_3) ppm. ^{13}C NMR (100 MHz, $CDCl_3$) δ 169.3, 160.9, 160.7, 156.2, 152.0, 146.4, 144.5, 137.2, 136.1, 128.7, 128.5, 128.1, 127.9, 123.4, 101.1, 61.5, 61.4, 56.6, 53.8, 51.8, 51.0, 38.9, 17.3, 14.1, 14.1 ppm. IR (KBr) υ: 3746, 2983, 2945, 1729, 1651, 1557, 1434, 1347, 1251, 1129, 1088, 841, 732, 709 cm^{-1}; MS (m/z): HRMS (ESI) Calcd. for $C_{29}H_{31}N_2O_8$ ($[M+H]^+$): 535.2075, found: 535.2076.

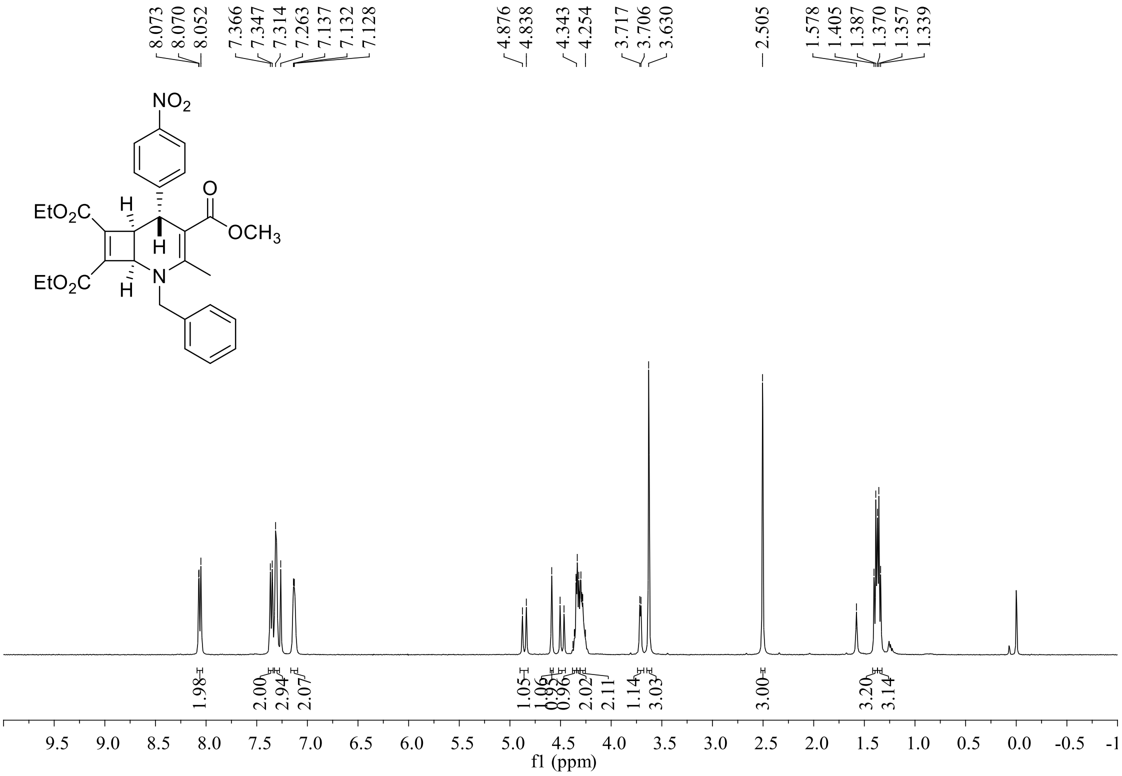

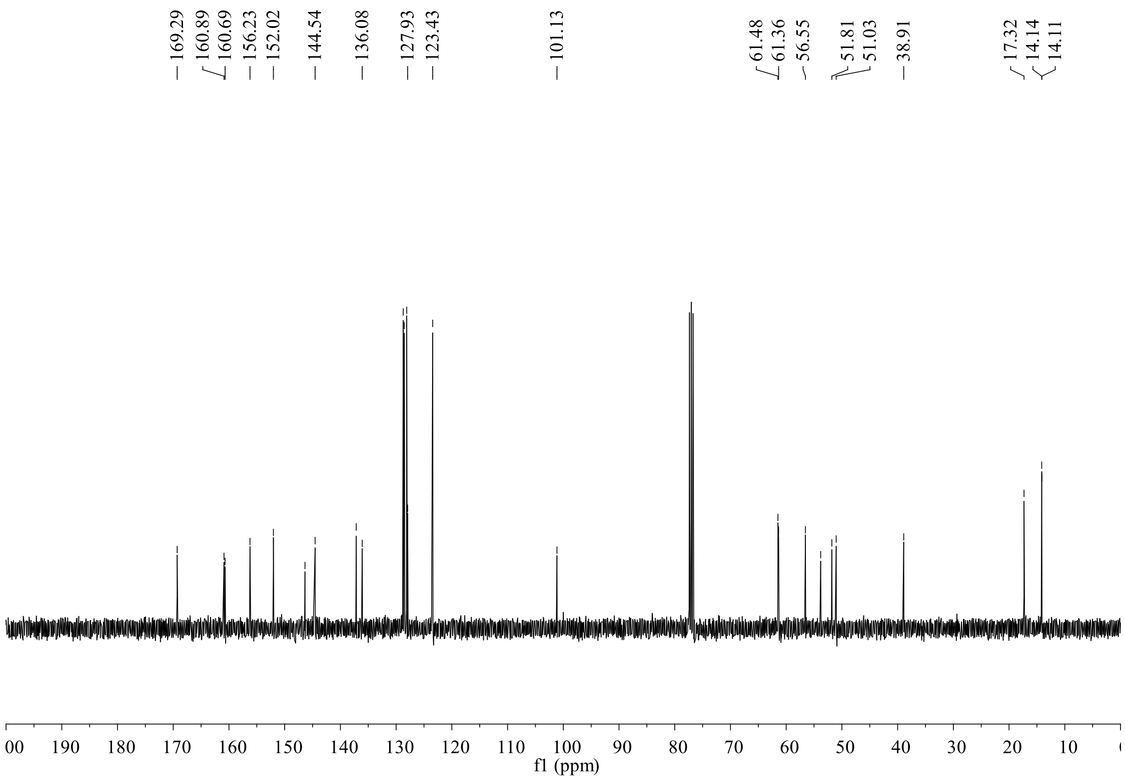
169.29
160.89
160.69
156.23
152.02
144.54
136.08
127.93
123.43
101.13
61.48
61.36
56.55
51.81
51.03
38.91
17.32
14.14
14.11
00 190 180 170 160 150 140 130 120 110 100 90 80 70 60 50 40 30 20 10
f1 (ppm)

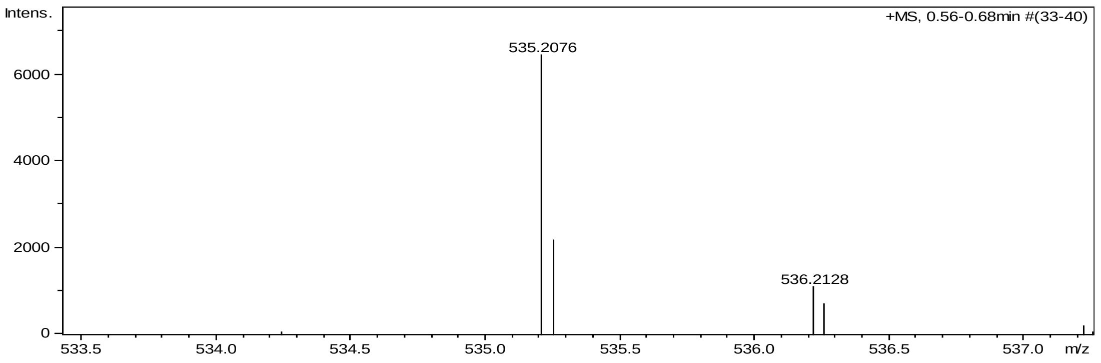
Intens.
+MS, 0.56-0.68min #(33-40)
535.2076
536.2128
6000
4000
2000
0
533.5 534.0 534.5 535.0 535.5 536.0 536.5 537.0 m/z

Trimethyl 2-(4-methoxybenzyl)-3-methyl-5-phenyl-2-azabicyclo[4.2.0]octa-3,7-diene-4,7,8-tricarboxylate (5f): yellow oily, 40%; ^{1}H NMR (400 MHz, $CDCl_3$) δ 7.23-7.18 (m, 5H, ArH), 7.03 (d, J = 8.4 Hz 2H, ArH), 6.80-6.77 (m, 2H, ArH), 4.75 (d, J = 15.2 Hz, 1H, CH_2), 4.48 (d, J = 1.2 Hz, 1H, CH), 4.38 (d, J = 15.2 Hz, 1H, CH_2), 4.27 (d, J = 5.2 Hz, 1H, CH), 3.83 (s, 6H, OCH_3), 3.79 (s, 3H, OCH_3), 3.77 (dd, J_1 = 4.8 Hz, J_2 = 1,2 Hz, 1H, CH), 3.62 (s, 3H, OCH_3), 2.46 (s, 3H, CH_3) ppm. ^{13}C NMR (100 MHz, $CDCl_3$) δ 169.8, 161.5, 161.3, 159.0, 155.8, 145.3, 143.8, 135.8, 129.4, 129.3, 128.2, 127.7, 126.1, 113.9, 102.2, 56.5, 55.3, 53.1, 52.9, 52.2, 52.2, 50.9, 38.6, 17.3 ppm. IR (KBr) υ: 2951, 2838, 2361, 1718, 1683, 1560, 1513, 1434, 1250, 1124, 1091, 972, 816, 701 cm^{-1}; MS (m/z): HRMS (ESI) Calcd. for $C_{28}H_{29}NaNO_7$ ($[M+Na]^+$): 514.1836, found: 514.1833.

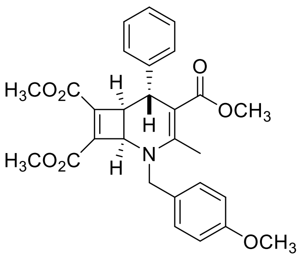

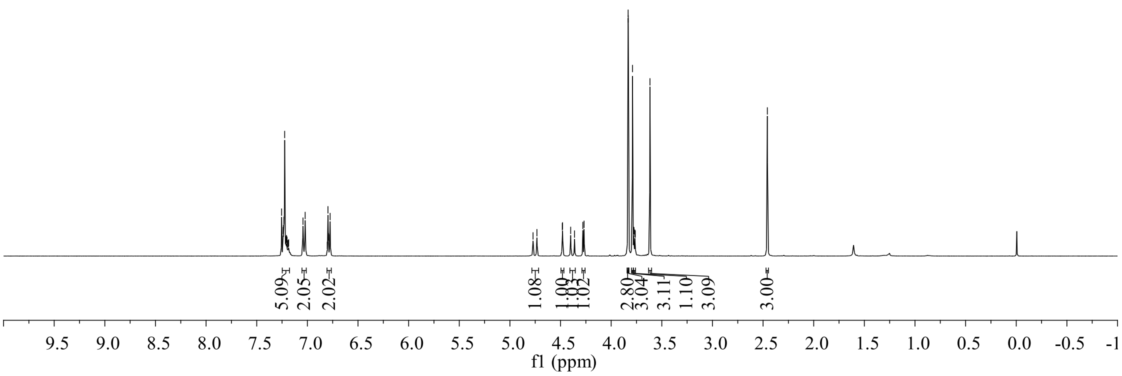

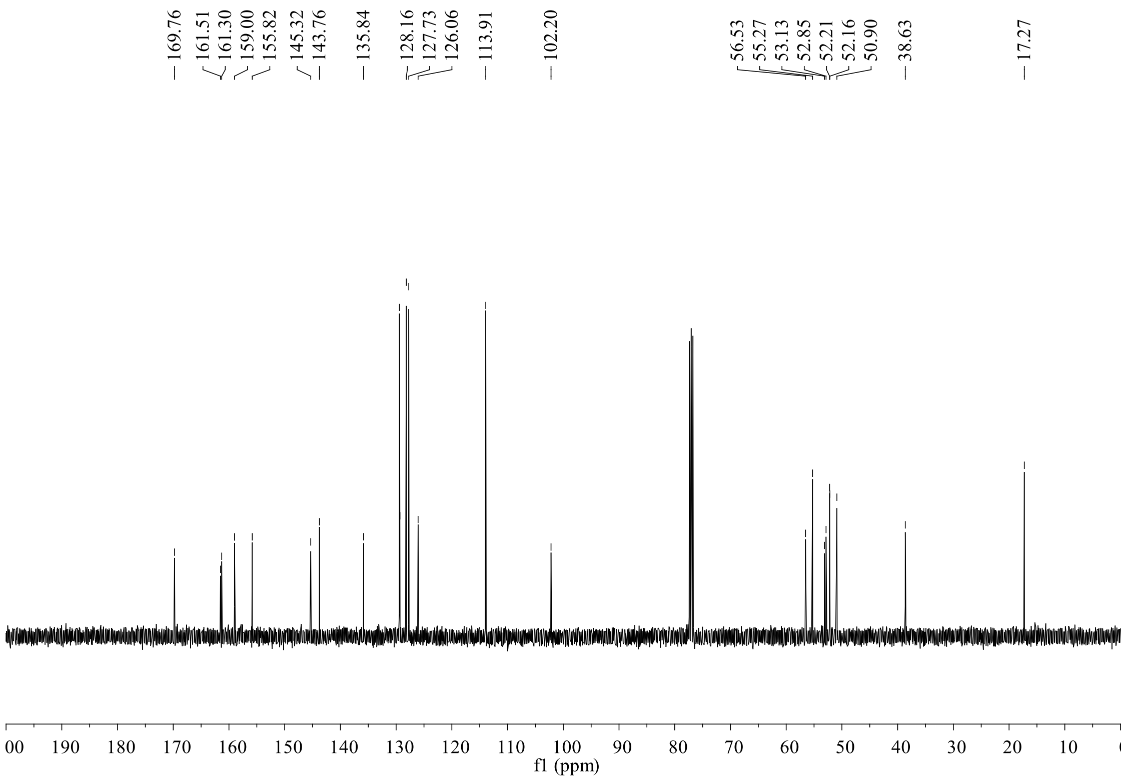
169.76
161.51
161.30
159.00
155.82
145.32
143.76
135.84
128.16
127.73
126.06
113.91
102.20
56.53
55.27
53.13
52.85
52.21
52.16
50.90
38.63
17.27
00 190 180 170 160 150 140 130 120 110 100 90 80 70 60 50 40 30 20 10
f1 (ppm)

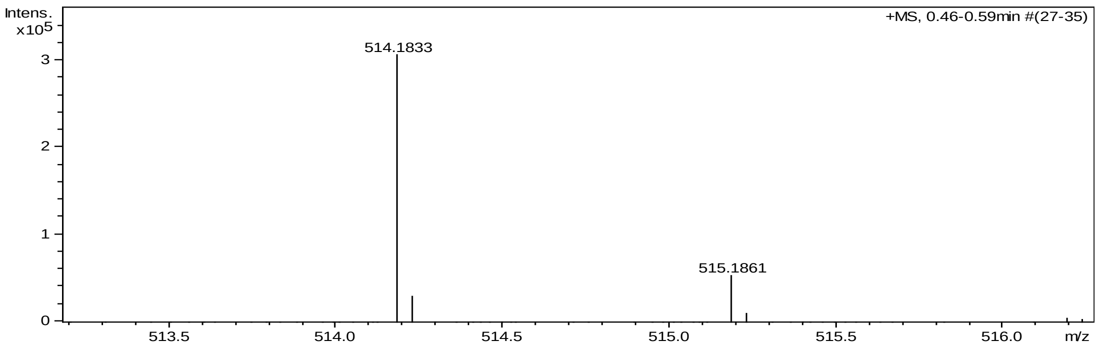
Intens.
x10^5
+MS, 0.46-0.59min #(27-35)
514.1833
515.1861
513.5 514.0 514.5 515.0 515.5 516.0 m/z

Trimethyl 2-(4-methoxybenzyl)-5-(2-methoxyphenyl)-3-methyl-2-azabicyclo[4.2.0]octa-3,7-diene-4,7,8-tricarboxylate (5g): yellow oily, 57%; ^{1}H NMR (400 MHz, $CDCl_3$) δ 7.23-7.18 (m, 1H, ArH), 7.10 (d, *J* = 8.8 Hz 2H, ArH), 7.06-7.02 (m, 1H, ArH), 6.88-6.82 (m, 2H, ArH), 6.78 (d, *J* = 8.4 Hz, 2H, ArH), 4.76 (d, *J* = 17.6 Hz, 2H, CH_2,CH), 4.41 (d, *J* = 15.6 Hz, 1H, CH_2), 4.13 (d, *J* = 4.8 Hz, 1H, CH), 3.86 (s, 6H, OCH_3), 3.82 (s, 3H, OCH_3), 3.77 (s, 3H, OCH_3), 3.73 (dd, J_1 = 4.8 Hz, J_2 = 1,2 Hz, 1H, CH), 3.53 (s, 3H, OCH_3), 2.52 (s, 3H, CH_3) ppm. ^{13}C NMR (100 MHz, $CDCl_3$) δ 169.9, 161.7, 161.6, 158.9, 157.5, 156.2, 146.6, 135.8, 130.0, 129.8, 129.1, 127.6, 127.4, 119.9, 113.9, 110.5, 100.8, 57.0, 55.4, 55.3, 53.4, 52.1, 52.1, 51.4, 50.9, 34.1, 17.2 ppm. IR (KBr) υ: 3728, 3626, 2951, 2837, 1735, 1685, 1561, 1436, 1237, 1182, 1126, 1099, 815, 755 cm^{-1}; MS (*m*/*z*): HRMS (ESI) Calcd. for $C_{29}H_{32}NO_8$ ($[M+H]^+$): 522.2122, found: 522.2123.

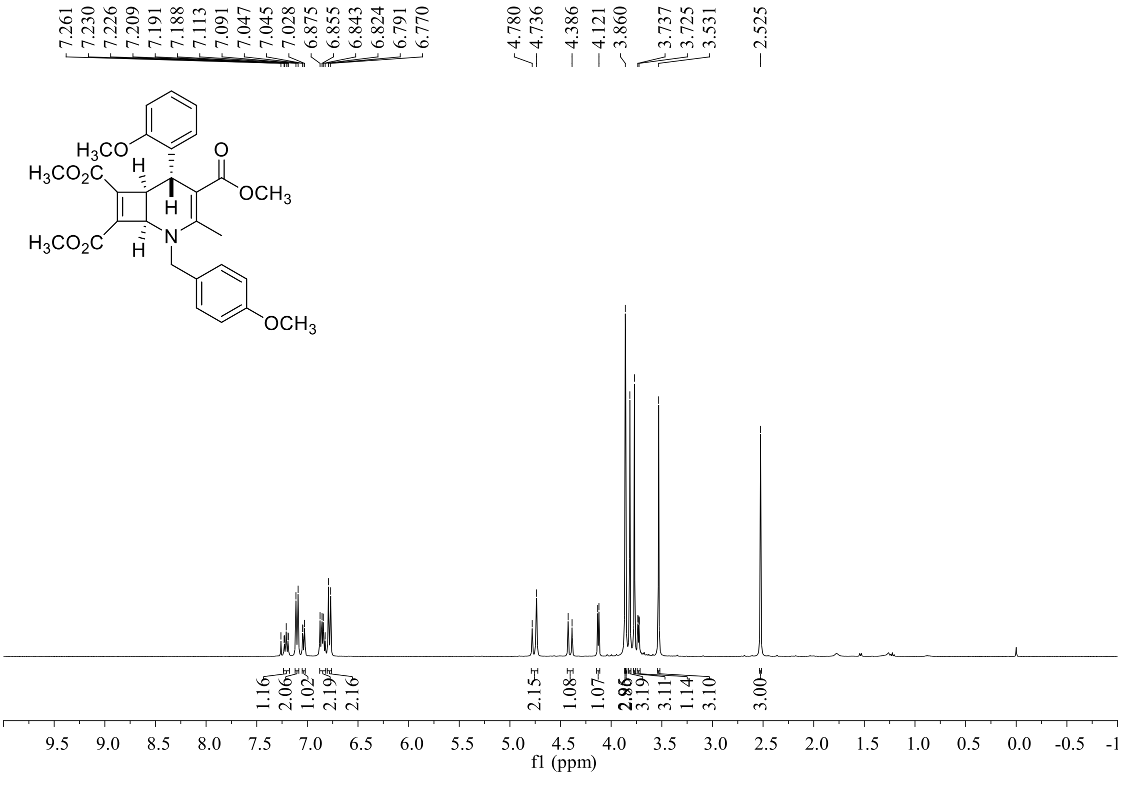

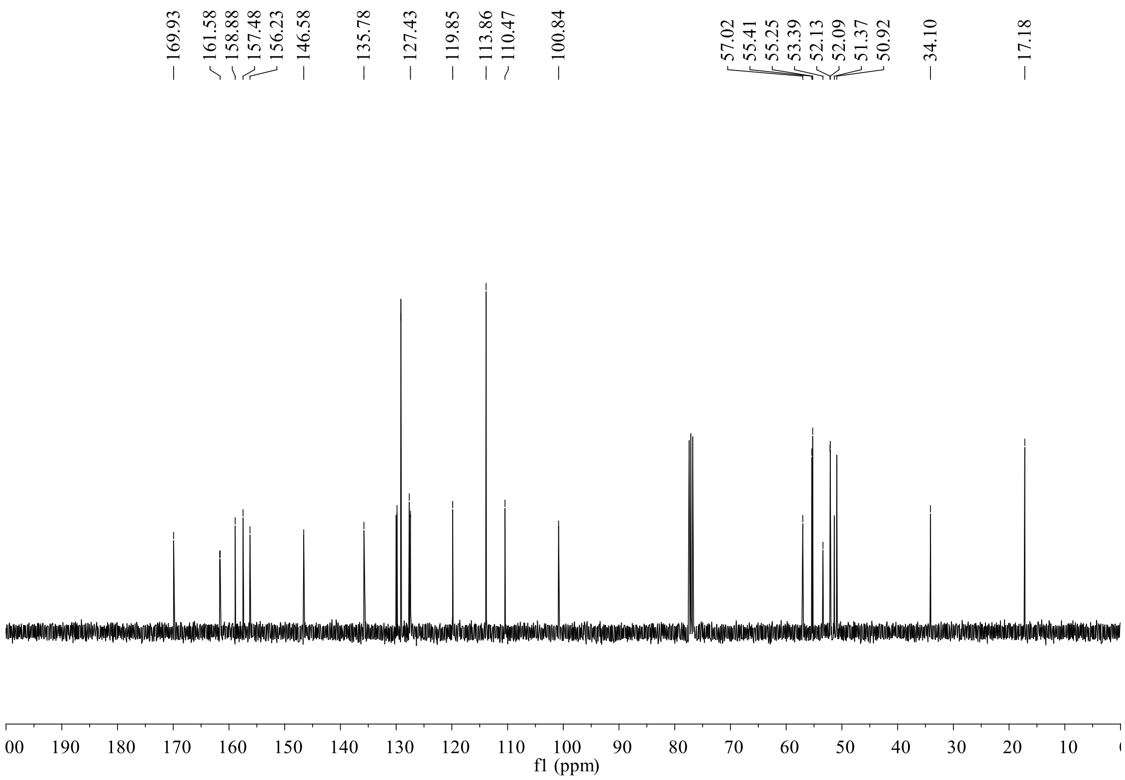
169.93
161.58
158.88
157.48
156.23
146.58
135.78
127.43
119.85
113.86
110.47
100.84
57.02
55.41
55.25
53.39
52.13
52.09
51.37
50.92
34.10
17.18
f1 (ppm)

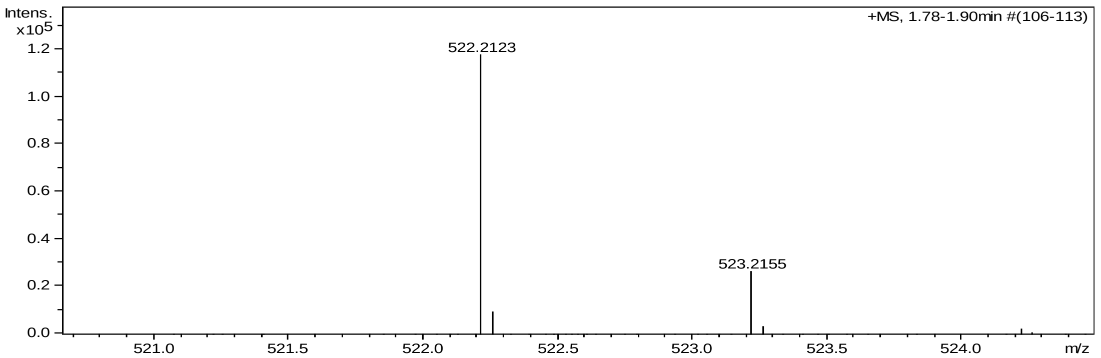
Intens.
x10^5
+MS, 1.78-1.90min #(106-113)
522.2123
523.2155
m/z

7,8-Diethyl 4-methyl 2-(4-methoxybenzyl)-5-(2-methoxyphenyl)-3-methyl-2-azabicyclo[4.2.0]octa-3,7-diene-4,7,8-tricarboxylate (5h): yellow solid, 62%, m.p. 147-149 ºC; ^{1}H NMR (400 MHz, $CDCl_3$) δ 7.23-7.18 (m, 1H, ArH), 7.10 (d, *J* = 8.4 Hz 2H, ArH), 7.05-7.03 (m, 1H, ArH), 6.87-6.82 (m, 2H, ArH), 6.78 (d, *J* = 8.8 Hz, 2H, ArH), 4.77 (d, *J* = 14.4 Hz, 2H, CH, CH_2), 4.41 (d, *J* = 15.6 Hz, 1H, CH_2), 4.36-4.29 (m 2H, CH_2), 4.29-4.22 (m, 2H, CH_2), 4.12 (d, *J* = 4.8 Hz, 1H, CH), 3.86 (s, 3H, OCH_3), 3.78 (s, 3H, OCH_3), 3.73-3.70 (m, 1H, CH), 3.53 (s, 3H, OCH_3), 2.54 (s, 3H, CH_3) 1.37 (t, *J* = 7.2 Hz, 3H, CH_3), 1.36 (t, *J* = 7.2 Hz, 3H, CH_3) ppm. ^{13}C NMR (100 MHz, $CDCl_3$) δ 169.9, 161.3, 161.2, 158.9, 157.5, 156.3, 146.8, 135.7, 130.1, 129.9, 129.2, 127.7, 127.4, 119.8, 113.9, 110.5, 100.9, 61.1, 61.0, 57.1, 55.4, 55.3, 53.4, 51.3, 50.9, 34.0, 17.1, 14.2, 14.2 ppm. IR (KBr) υ: 3726, 3696, 2981, 2841, 1733, 1678, 1568, 1308, 1234, 1099, 1029, 954, 837, 756 cm^{-1}; MS (*m*/*z*): HRMS (ESI) Calcd. for $C_{31}H_{35}NaNO_8$ ($[M+Na]^+$): 572.2255, found: 572.2246.

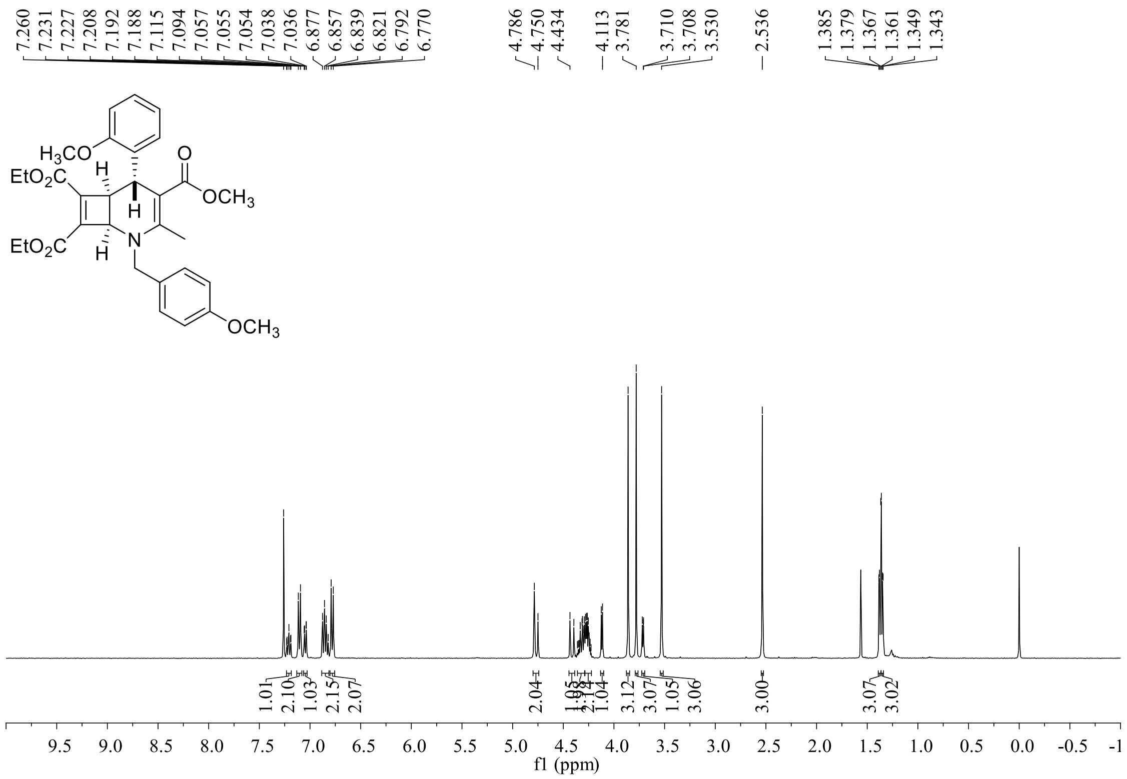

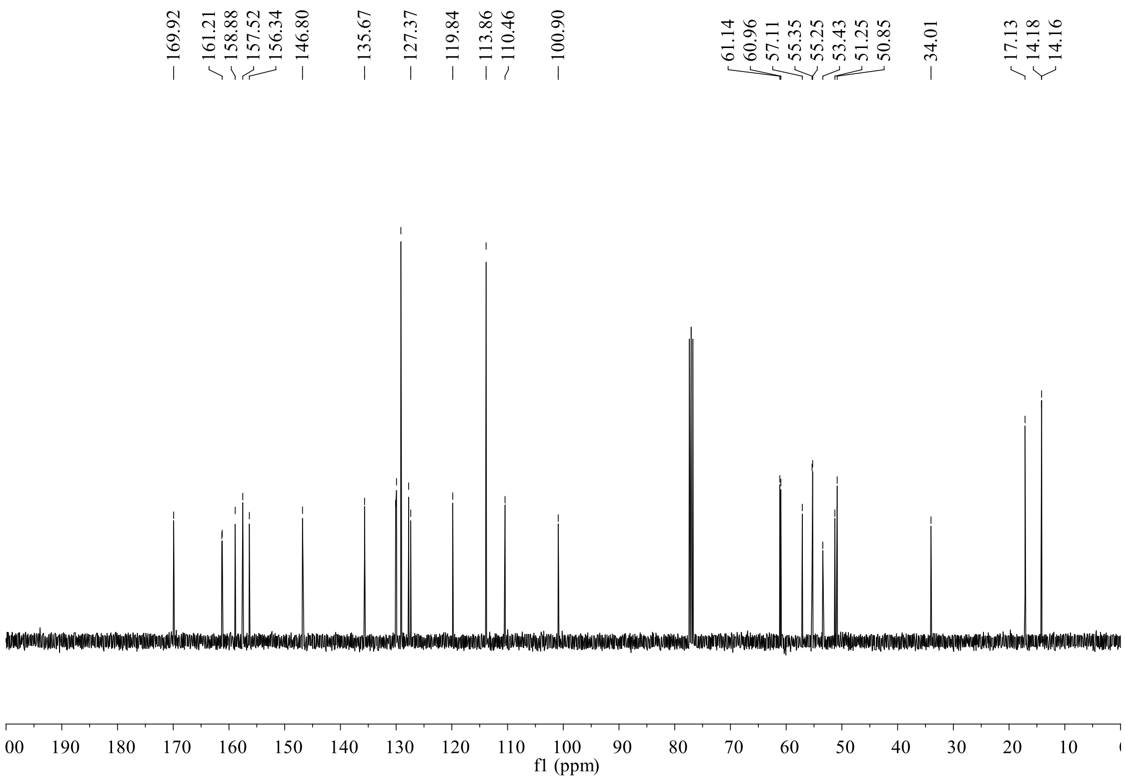
169.92
161.21
158.88
157.52
156.34
146.80
135.67
127.37
119.84
113.86
110.46
100.90
61.14
60.96
57.11
55.35
55.25
53.43
51.25
50.85
34.01
17.13
14.18
14.16
f1 (ppm)

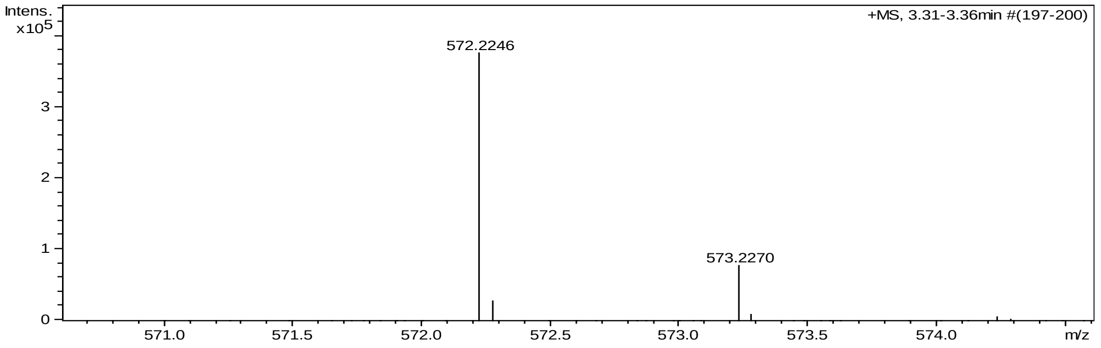
Intens.
x10^5
+MS, 3.31-3.36min #(197-200)
572.2246
573.2270
m/z

7,8-Diethyl 4-methyl 2-(4-methoxybenzyl)-3-methyl-5-(4-nitrophenyl)-2-azabicyclo[4.2.0]octa-3,7-diene-4,7,8-tricarboxylate (5i): yellow solid, 41%, m.p. 142-144 °C; ^{1}H NMR (400 MHz, $CDCl_3$) δ 8.06 (d, J = 8.8 Hz, 2H, ArH), 7.34 (d, J = 8.8 Hz 2H, ArH), 7.06 (d, J = 8.8 Hz, 2H, ArH), 6.84 (d, J = 8.8 Hz 2H, ArH), 4.79 (d, J = 14.8 Hz, 1H, CH_2), 4.57 (s, 1H, CH), 4.41 (d, J = 15.2 Hz, 1H, CH_2), 4.36-4.33 (m 1H, CH), 4.33-4.31 (m, 2H, CH_2), 4.31-4.24 (m, 2H, CH_2), 3.82 (s, 3H, OCH_3), 3.69 (dd, J_1 = 5.2 Hz, J_2 = 1,6 Hz, 1H, CH), 3.63 (s, 3H, OCH_3), 2.51 (s, 3H, CH_3), 1.37 (t, J = 7.2 Hz, 3H, CH_3), 1.36 (t, J = 7.2 Hz, 3H, CH_3) ppm. ^{13}C NMR (100 MHz, $CDCl_3$) δ 169.3, 160.9, 160.7, 159.3, 156.3, 152.1, 146.3, 144.5, 136.1, 129.6, 128.9, 128.5, 123.4, 114.1, 101.1, 61.5, 61.3, 55.9, 55.3, 53.1, 51.8, 51.0, 38.9, 17.3, 14.1, 14.1 ppm. IR (KBr) υ: 3727, 3629, 2984, 2838, 1729, 1652, 1514, 1346, 1241, 1128, 1084, 968, 823, 766 cm^{-1}; MS (m/z): HRMS (ESI) Calcd. for $C_{30}H_{32}NaN_2O_9$ ($[M+Na]^+$): 587.2000, found: 587.1991.

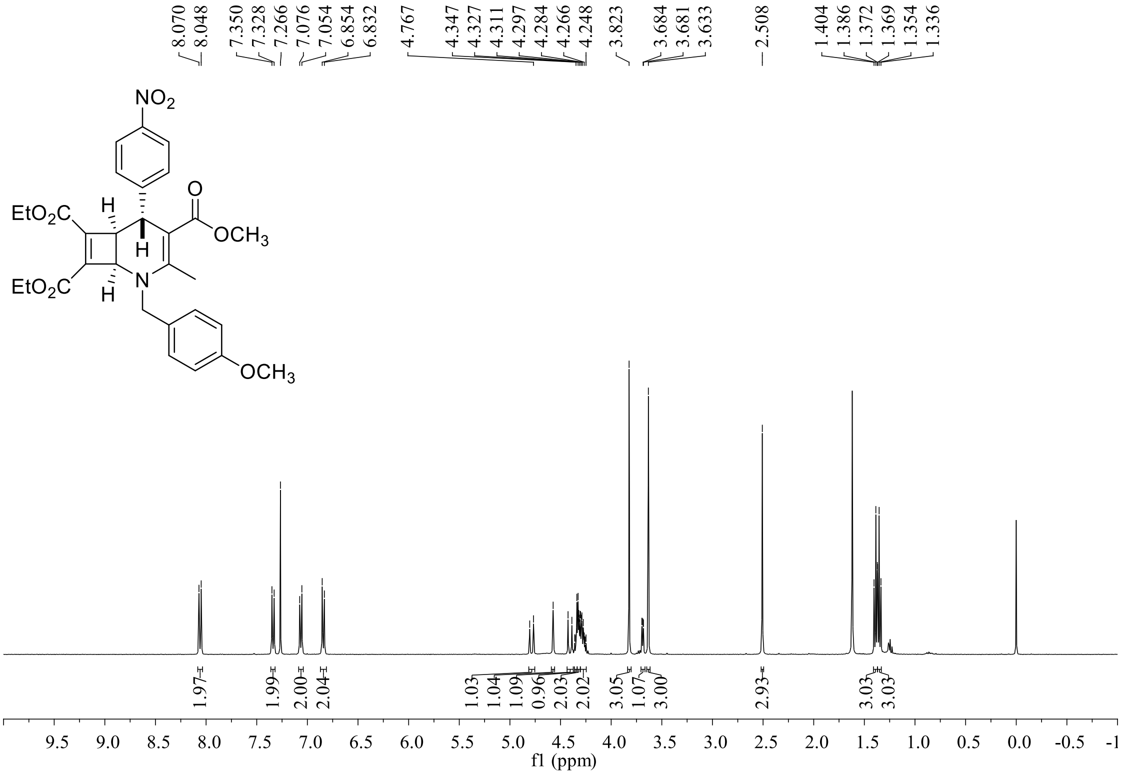

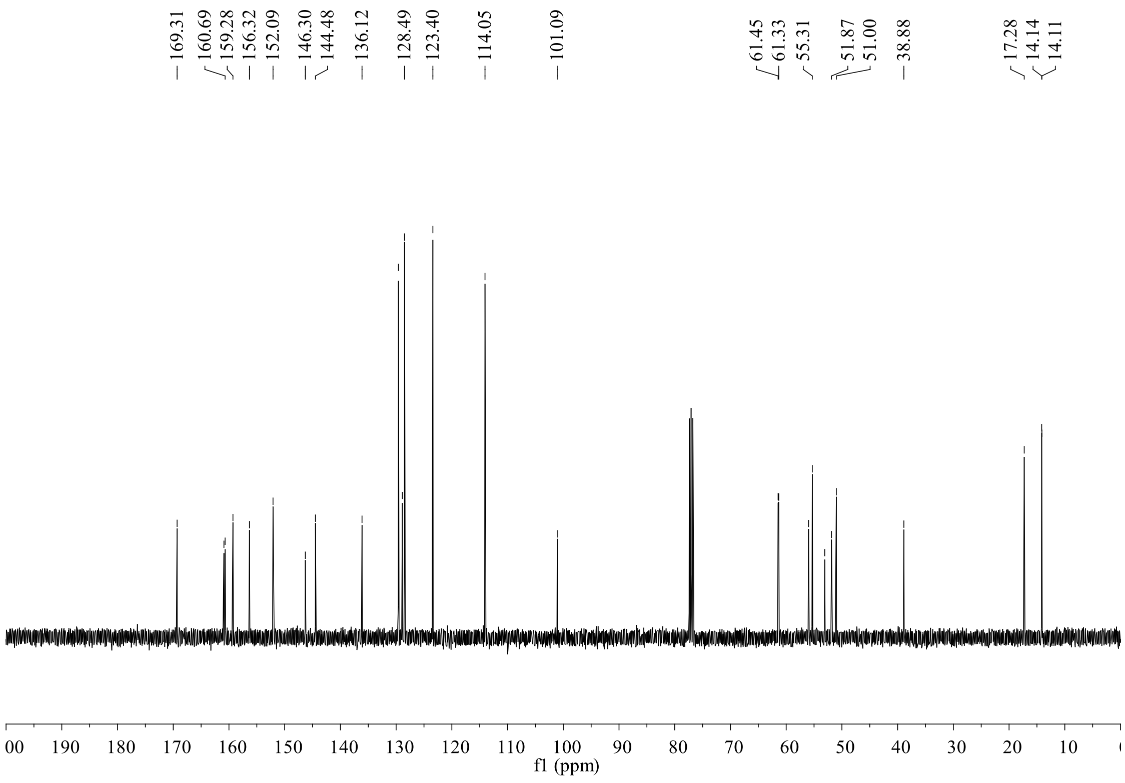

169.31
160.69
159.28
156.32
152.09
146.30
144.48
136.12
128.49
123.40
114.05
101.09
61.45
61.33
55.31
51.87
51.00
38.88
17.28
14.14
14.11
f1 (ppm)

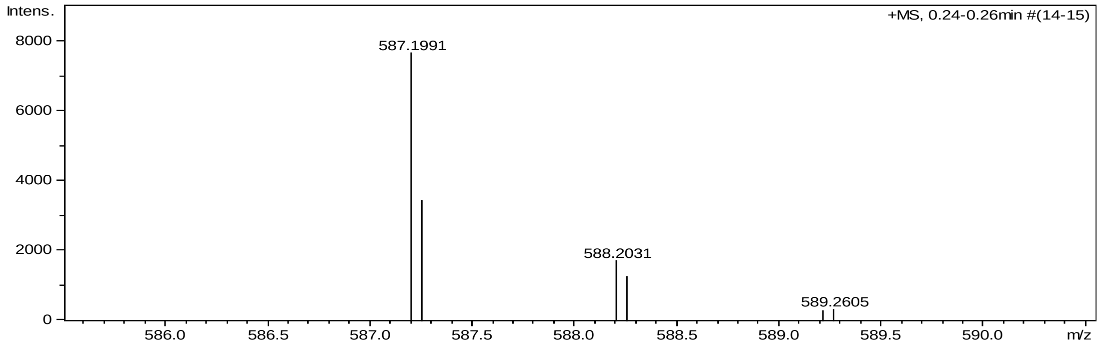

Intens.
+MS, 0.24-0.26min #(14-15)
587.1991
588.2031
589.2605
m/z

Trimethyl 2-(4-methoxybenzyl)-3-methyl-5-(4-nitrophenyl)-2-azabicyclo[4.2.0]octa-3,7-diene-4,7,8-tricarboxylate (5j): yellow solid, 64%, m.p. 158-160 °C; ^{1}H NMR (400 MHz, $CDCl_3$) δ 8.06 (d, *J* = 8.8 Hz, 2H, ArH), 7.34 (d, *J* = 8.4 Hz 2H, ArH), 7.07 (d, *J* = 8.4 Hz, 2H, ArH), 6.85 (d, *J* = 8.4 Hz 2H, ArH), 4.78 (d, *J* = 15.2 Hz, 1H, CH_2), 4.55 (s, 1H, CH), 4.40 (d, *J* = 14.8 Hz, 1H, CH_2), 4.34 (d, *J* = 4.8 Hz, 1H, CH), 3.86 (s, 3H, OCH_3), 3.85 (s, 3H, OCH_3), 3.82 (s, 3H, OCH_3), 3.71 (d, *J* = 4.4 Hz, 1H, CH), 3.64 (s, 3H, OCH_3), 2.50 (s, 3H, CH_3) ppm. ^{13}C NMR (100 MHz, $CDCl_3$) δ 169.3, 161.3, 161.0, 159.3, 156.2, 152.0, 146.3, 144.4, 136.2, 129.6, 128.8, 128.5, 123.4, 114.1, 101.1, 55.9, 55.3, 53.1, 52.4, 52.3, 52.0, 51.1, 38.9, 17.4 ppm. IR (KBr) υ: 3854, 3650, 2948, 2836, 1706, 1678, 1521, 1348, 1238, 1184, 1089, 969, 850, 740 cm^{-1}; MS (*m*/*z*): HRMS (ESI) Calcd. for $C_{28}H_{28}NaN_2O_9$([M+Na]$^+$): 559.1687, found: 559.1685.

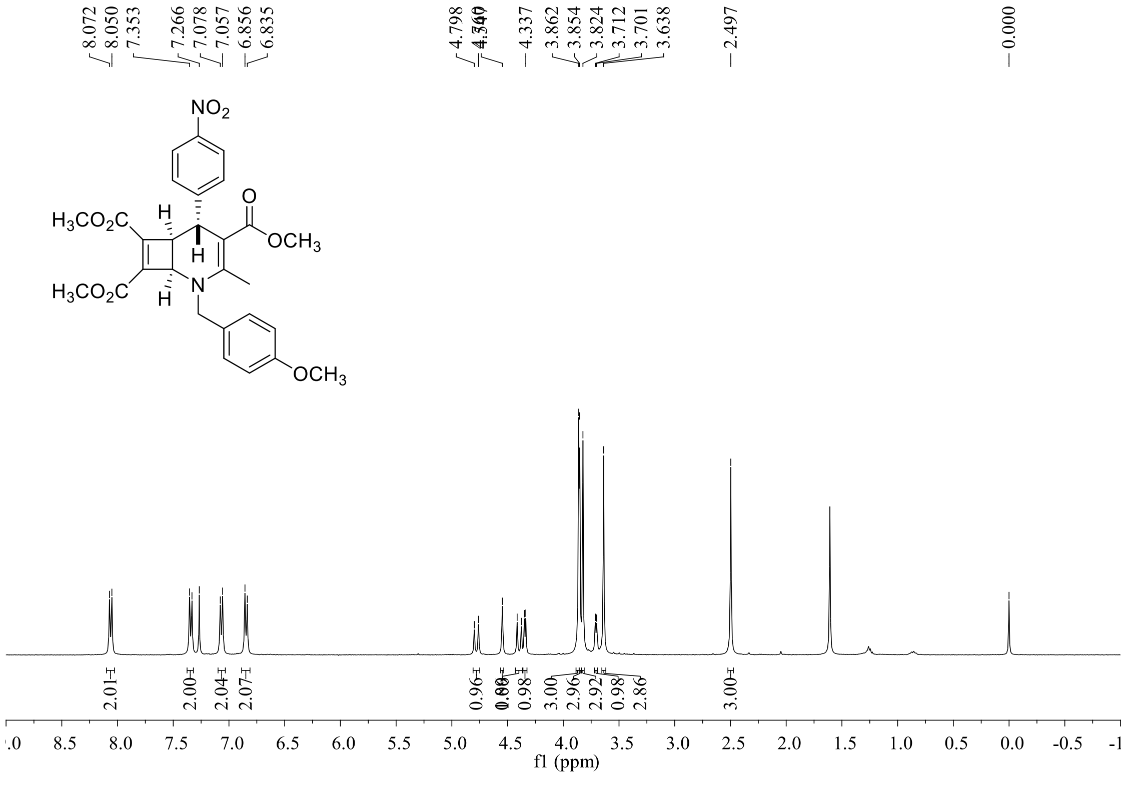

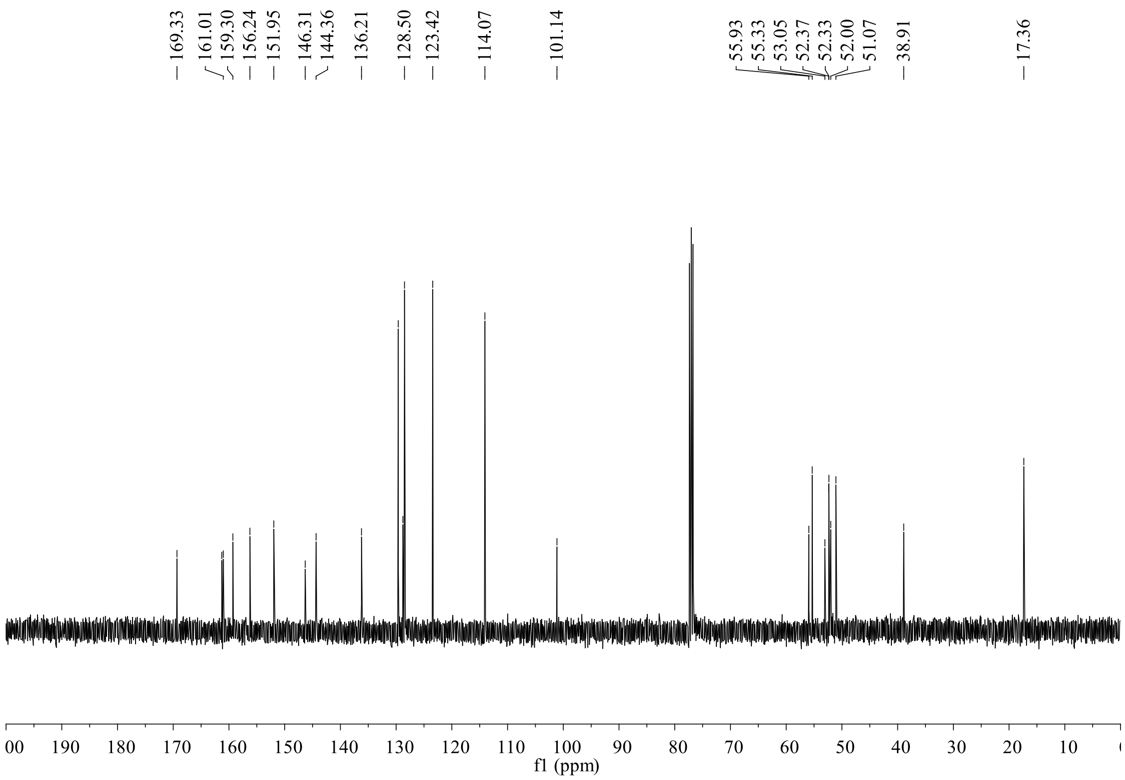
169.33
161.01
159.30
156.24
151.95
146.31
144.36
136.21
128.50
123.42
114.07
101.14
55.93
55.33
53.05
52.37
52.33
52.00
51.07
38.91
17.36
f1 (ppm)

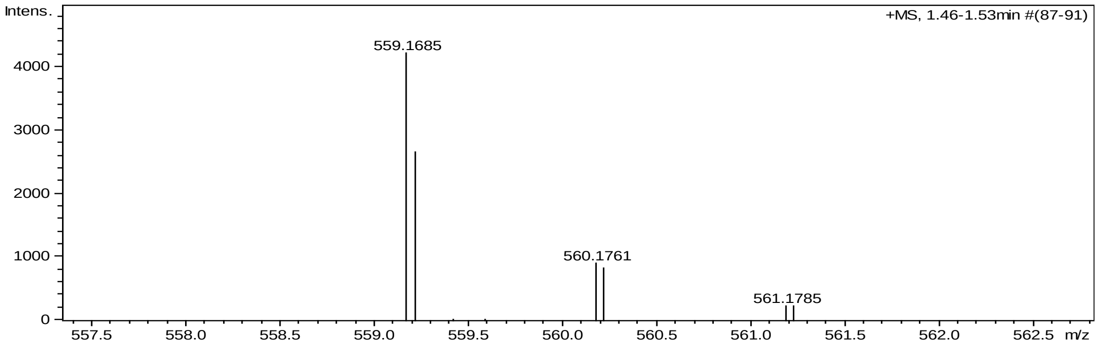
Intens.
+MS, 1.46-1.53min #(87-91)
559.1685
560.1761
561.1785
m/z

Trimethyl 2-(4-methoxyphenyl)-3-methyl-5-phenyl-2-azabicyclo[4.2.0]octa-3,7-diene-4,7,8-tricarboxylate (5k): yellow solid, 35%, m.p. 182-184 ºC; ^{1}H NMR (400 MHz, $CDCl_3$) δ 7.34-7.32 (m, 2H, ArH), 7.25-7.16 (m, 3H, ArH), 7.06 (d, J = 8.4 Hz, 2H, ArH), 6.83 (d, J = 9.2 Hz, 2H, ArH), 4.77 (d, J = 4.4 Hz, 1H, CH), 4.60 (s 1H, CH), 4.10 (dd, J_1 = 4.8 Hz, J_2 = 1.2 Hz, 1H, CH), 3.85 (s, 6H, OCH_3), 3.79 (s, 3H, OCH_3), 3.71 (s, 3H, OCH_3), 2.12 (s, 3H, CH_3) pm. ^{13}C NMR (100 MHz, $CDCl_3$) δ 169.1, 161.5, 161.3, 156.8, 153.5, 144.7, 144.6, 138.2, 137.0, 128.4, 127.4, 126.2, 125.8, 114.2, 109.6, 59.3, 56.6, 55.5, 52.2, 52.2, 51.1, 39.1, 20.4 ppm. IR (KBr) υ: 3726, 3601, 2951, 1719, 1695, 1576, 1510, 1437, 1380, 1228, 1092, 962, 833, 788 cm^{-1}; MS (m/z): HRMS (ESI) Calcd. for $C_{27}H_{27}NaNO_7$ ($[M+Na]^+$): 566.1680, found: 500.1676.

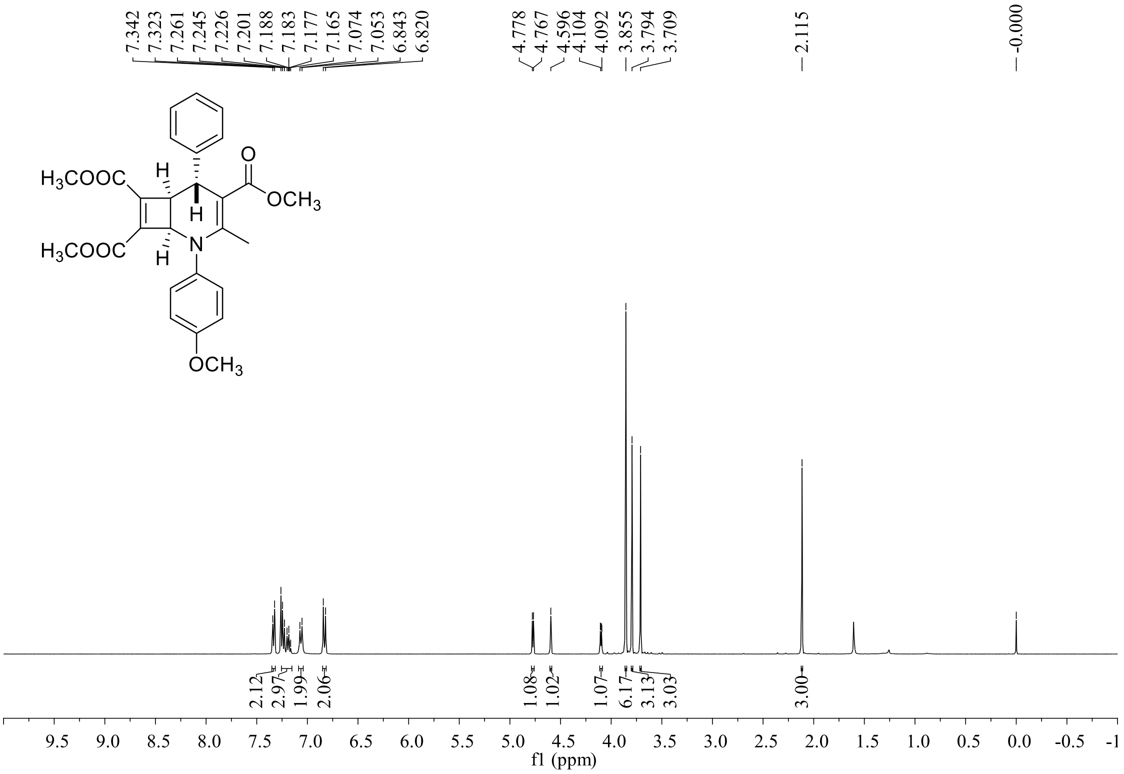

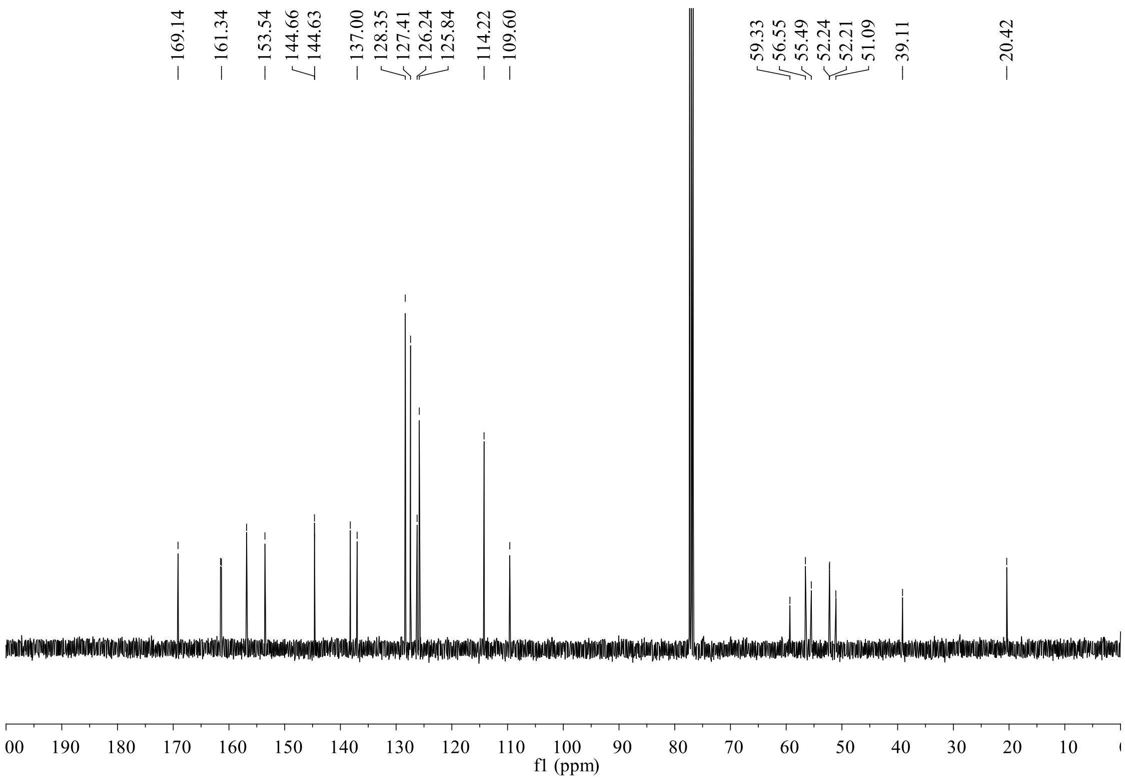
169.14
161.34
153.54
144.66
144.63
137.00
128.35
127.41
126.24
125.84
114.22
109.60
59.33
56.55
55.49
52.24
52.21
51.09
39.11
20.42
f1 (ppm)

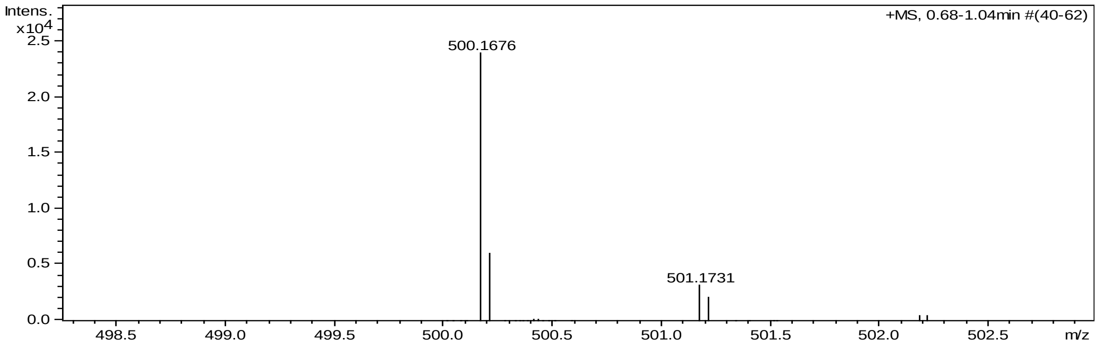
Intens.
x10^4
+MS, 0.68-1.04min #(40-62)
500.1676
501.1731
m/z

7,8-Diethyl 4-methyl 3-methyl-5-phenyl-2-(*p*-tolyl)-2-azabicyclo[4.2.0]octa-3,7-diene-4,7,8-tricarboxylate (5l): yellow oily, 31%; ^{1}H NMR (400 MHz, $CDCl_3$) δ 7.33-7.30 (m, 2H, ArH), 7.25-7.20 (m, 2H, ArH), 7.19-7.15 (m, 1H, ArH), 7.09 (d, *J* = 8.0 Hz, 2H, ArH), 7.01 (d, *J* = 8.0 Hz, 2H, ArH), 4.83 (d, *J* = 4.8 Hz, 1H, CH), 4.62 (s, 1H, CH), 4.36-4.30 (m, 2H,CH_2), 4.30-4.24 (m, 2H,CH_2), 4.07 (dd, J_1 = 4.8 Hz, J_2 = 1.6 Hz, 1H, CH), 3.71 (s, 3H, OCH_3), 2.32 (s, 3H, CH_3), 2.15 (s, 3H, CH_3), 1.36 (t, *J* = 7.2 Hz, 3H, CH_3), 1.33 (d, *J* = 7.2 Hz, 3H, CH_3) ppm. ^{13}C NMR (100 MHz, $CDCl_3$) δ 169.0, 161.0, 161.0, 153.2, 144.8, 144.6, 142.6, 137.2, 133.9, 129.5, 128.4, 127.4, 126.2, 124.0, 110.9, 61.3, 61.2, 59.2, 56.4, 51.1, 39.2, 20.8, 20.6, 14.2, 14.1 ppm. IR (KBr) υ: 3726, 3696, 2982, 1701, 1685, 1577, 1512, 1438, 1380, 1226, 1092, 820, 777, 716 cm^{-1}; MS (*m*/*z*): HRMS (ESI) Calcd. for $C_{29}H_{31}NaNO_6$ ([M+Na]$^{+}$): 512.2044, found: 512.2035.

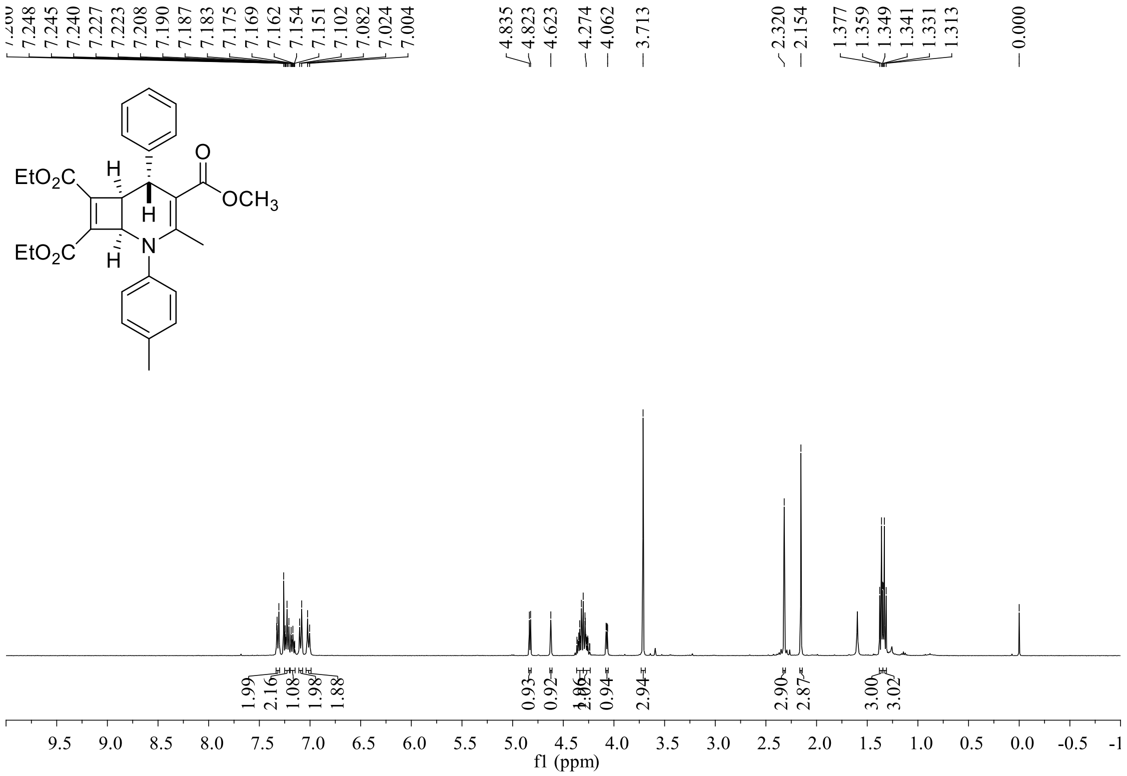

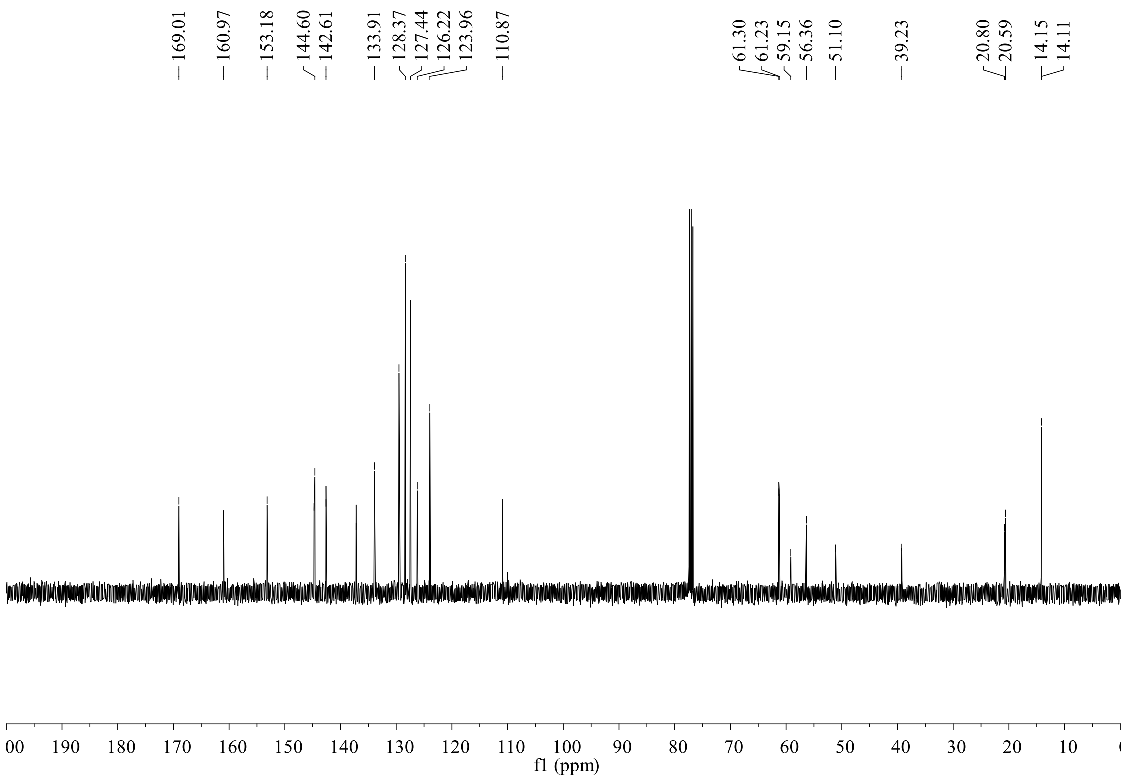
169.01
160.97
153.18
144.60
142.61
133.91
128.37
127.44
126.22
123.96
110.87
61.30
61.23
59.15
56.36
51.10
39.23
20.80
20.59
14.15
14.11
f1 (ppm)

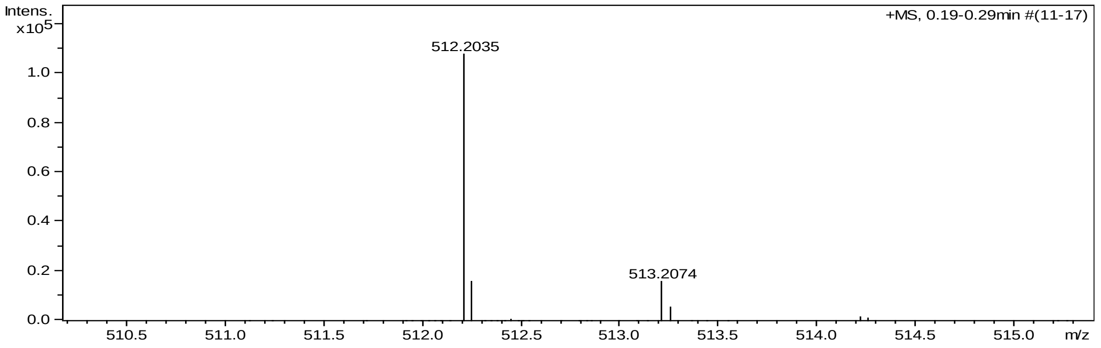
Intens.
x10^5
+MS, 0.19-0.29min #(11-17)
512.2035
513.2074
m/z

Trimethyl 2-(4-bromophenyl)-3-methyl-5-phenyl-2-azabicyclo[4.2.0]octa-3,7-diene-4,7,8-tricarboxylate (5m): yellow oily, 33%; ^{1}H NMR (400 MHz, $CDCl_3$) δ 7.39 (d, J = 8.8 Hz, 2H, ArH), 7.28-7.27 (m, 1H, ArH), 7.25-7.22 (m, 2H, ArH), 7.22-7.13 (m, 2H, ArH), 6.97 (d, J = 8.8 Hz, 2H, ArH), 4.84 (d, J = 4.8 Hz, 1H, CH), 4.61 (s, 1H, CH), 4.11 (dd, J_1 = 4.4 Hz, J_2 = 1.2 Hz, 1H, CH), 3.86 (s, 3H, OCH_3), 3.85 (s, 3H, OCH_3), 3.73 (s, 3H, OCH_3), 2.13 (s, 3H, CH_3) ppm. ^{13}C NMR (100 MHz, $CDCl_3$) δ 168.7, 161.2, 161.2, 151.7, 145.1, 144.1, 144.0, 136.7, 132.0, 128.5, 127.3, 126.4, 125.1, 117.0, 113.1, 58.8, 56.2, 52.3, 52.3, 51.3, 39.1, 20.6 ppm. IR (KBr) υ: 3726, 3696, 2950, 1701, 1654, 1577, 1491, 1437, 1380, 1228, 1092, 1008, 827, 702 cm^{-1}; MS (m/z): HRMS (ESI) Calcd. for $C_{26}H_{24}BrNaNO_6$ ($[M+Na]^+$): 548.0679, found: 548.0680.

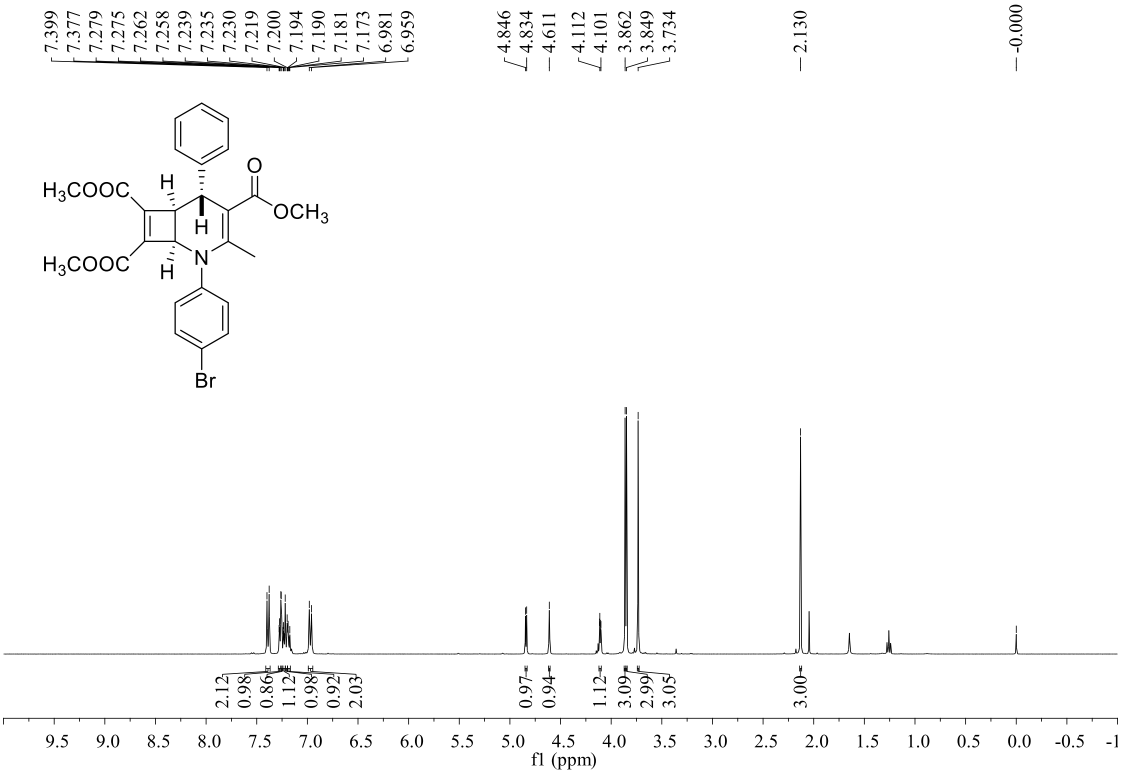

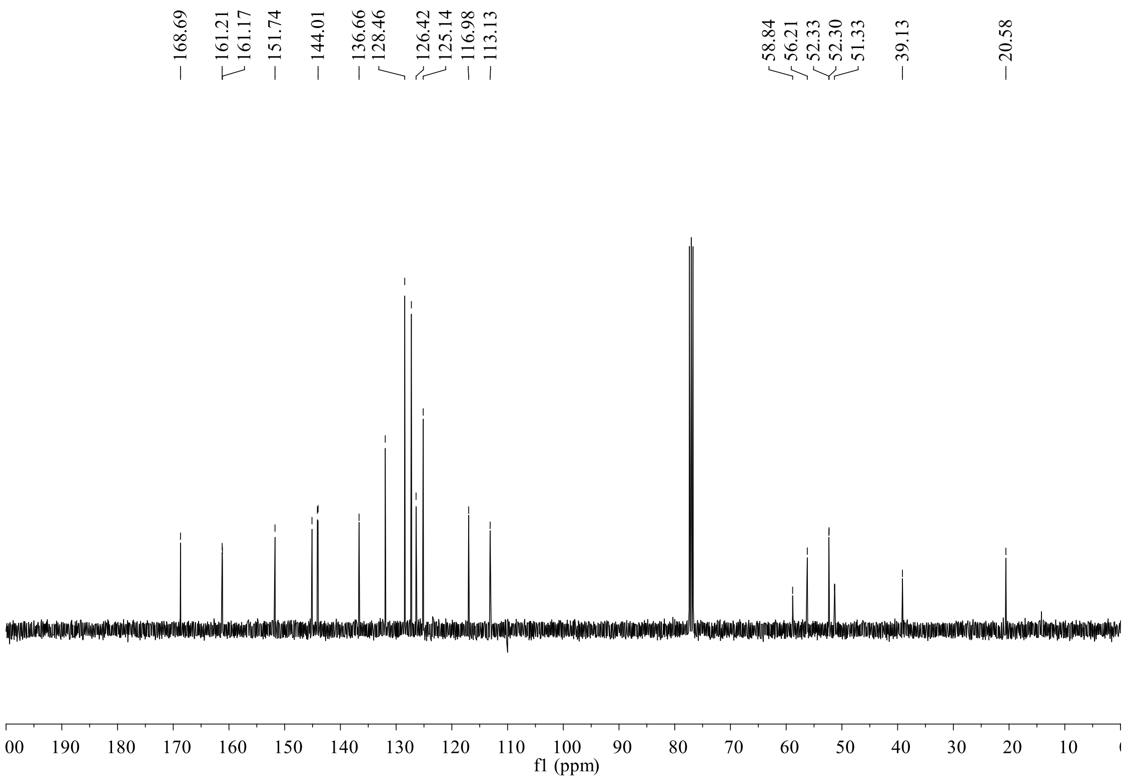
168.69
161.21
161.17
151.74
144.01
136.66
128.46
126.42
125.14
116.98
113.13
58.84
56.21
52.33
52.30
51.33
39.13
20.58
f1 (ppm)

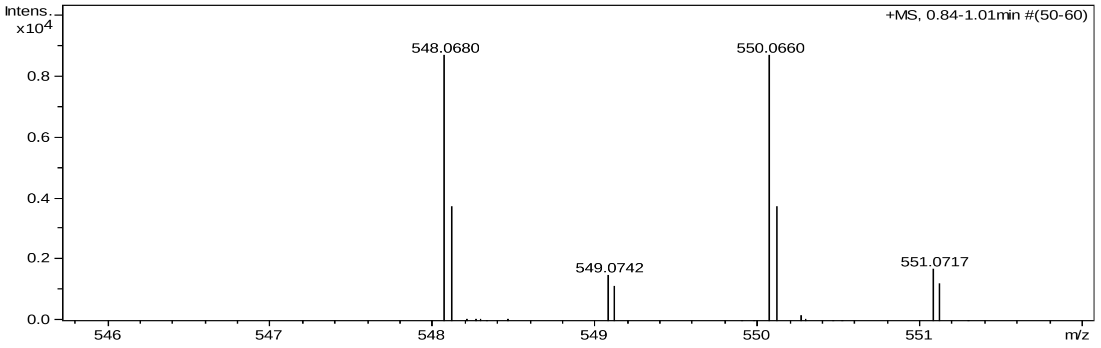
Intens.
x10^4
+MS, 0.84-1.01min #(50-60)
548.0680
550.0660
549.0742
551.0717
m/z

Trimethyl 2-(3-chlorophenyl)-3-methyl-5-phenyl-2-azabicyclo[4.2.0]octa-3,7-diene-4,7,8-tricarboxylate (5n): yellow oily, 55%; ^{1}H NMR (400 MHz, $CDCl_3$) δ 7.29-7.27 (m, 1H, ArH), 7.25-7.22 (m, 2H, ArH), 7.21-7.18 (m, 2H, ArH), 7.18-7.16 (m, 2H, ArH), 7.07-7.04 (m, 1H, ArH), 6.93-6.90 (m, 1H, ArH), 4.86 (d, J = 4.8 Hz, 1H, CH), 4.61 (s, 1H, CH), 4.10 (dd, J_1 = 4.8 Hz, J_2 = 1.2 Hz, 1H, CH), 3.86 (s, 6H, OCH_3), 3.73 (s, 3H, OCH_3), 2.15 (s, 3H, CH_3). ^{13}C NMR (100 MHz, $CDCl_3$) δ 168.7, 161.2, 161.1, 151.6, 146.3, 145.2, 143.9, 136.8, 134.6, 129. 8, 128.5, 127.3, 126.5, 123.9, 123.5, 121.7, 113.5, 58.8, 56.1, 52.3, 52.3, 51.3, 39.2, 20.6. IR (KBr) υ: 3726, 3601, 2951, 1709, 1651, 1586, 1434, 1378, 1227, 1160, 1091, 983, 866, 776 cm^{-1}; MS (m/z): HRMS (ESI) Calcd. for $C_{26}H_{24}ClNaNO_6$ ($[M+Na]^+$): 504.1184, found: 504.1188.

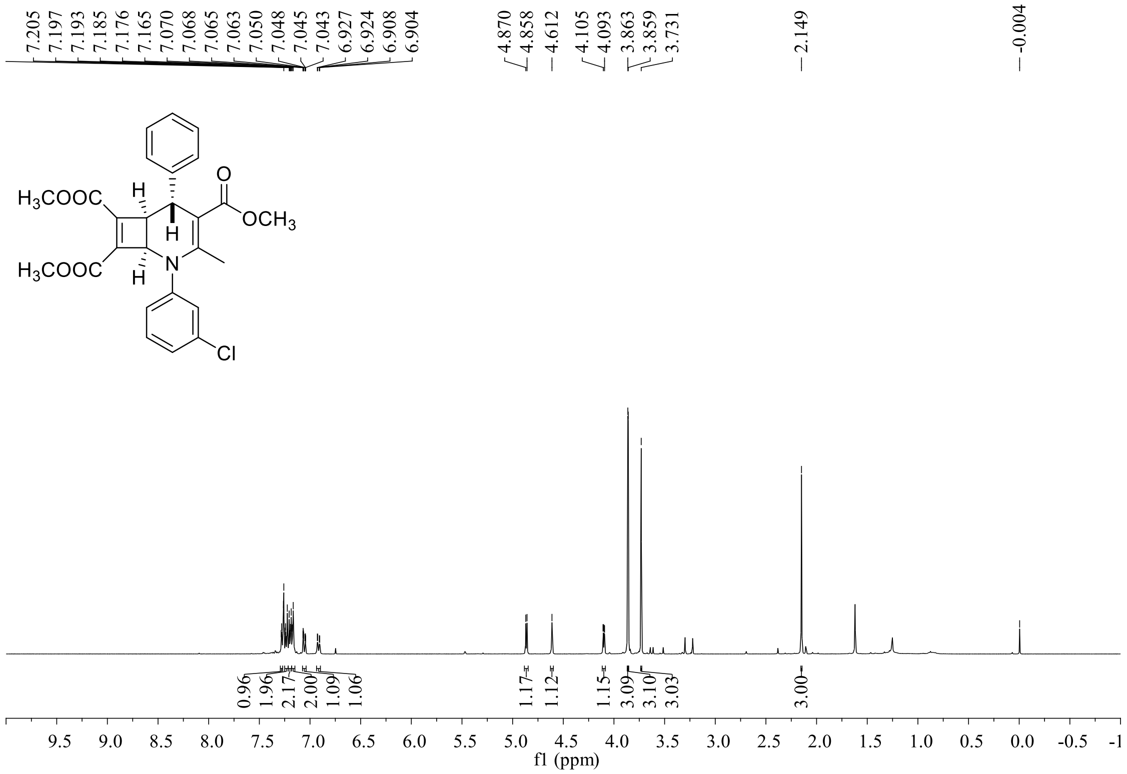

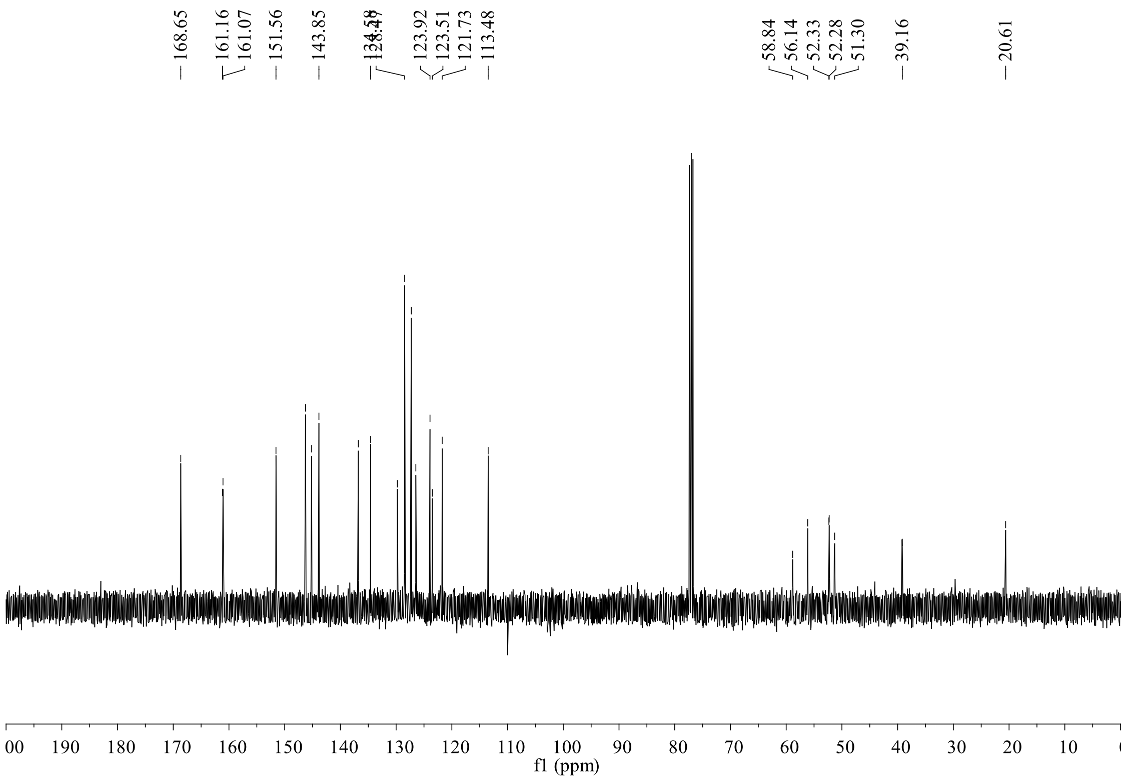
168.65
161.16
161.07
151.56
143.85
134.58
128.47
123.92
123.51
121.73
113.48
58.84
56.14
52.33
52.28
51.30
39.16
20.61
f1 (ppm)

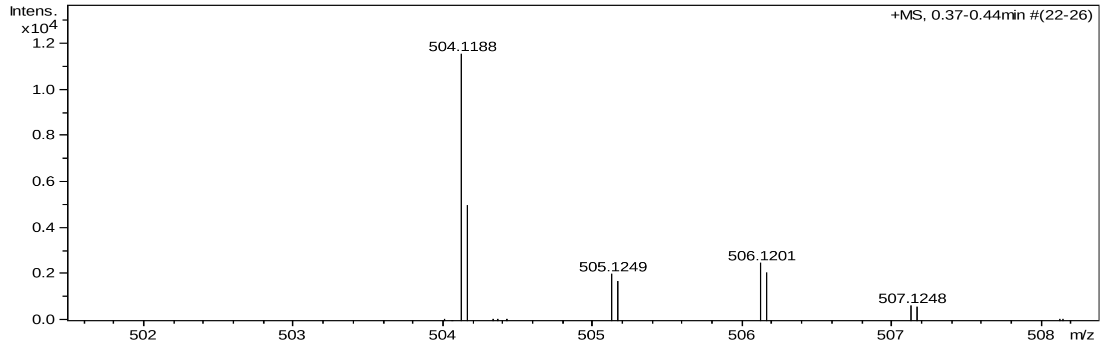
Intens.
x10^4
+MS, 0.37-0.44min #(22-26)
504.1188
505.1249
506.1201
507.1248
m/z

Trimethyl 5-(2-methoxyphenyl)-3-methyl-2-(*o*-tolyl)-2-azabicyclo[4.2.0]octa-3,7-diene-4,7,8-tricarboxylate (5o): yellow solid, 64%, m.p. 144-146 ºC; ^{1}H NMR (400 MHz, CDCl$_3$) δ 7.22-7.16 (m, 4H, ArH), 7.15-7.13 (m, 2H, ArH), 6.89 (d, *J* = 8.0 Hz, 1H, ArH), 6.82 (t, *J* = 7.2 Hz, 1H, ArH), 4.80 (s, 1H, CH), 4.47 (d, *J* = 4.8 Hz, 1H, CH), 3.98 (d, *J* = 3.6 Hz, 1H, CH), 3.87 (s, 3H, OCH$_3$), 3.86 (s, 3H, OCH$_3$), 3.71 (s, 3H, OCH$_3$), 3.61 (s, 3H, OCH$_3$), 2.07 (s, 3H, CH$_3$), 1.84 (s, 3H, CH$_3$) ppm. ^{13}C NMR (100 MHz, CDCl$_3$) δ 169.7, 162.2, 161.5, 157.5, 153.3, 144.1, 142.6, 138.3, 135.3, 131.3, 131.0, 128.0, 127.4, 127.2, 126.7, 126.3, 119.8, 110.6, 103.2, 59.3, 55.4, 52.1, 52.0, 51.8, 51.0, 34.3, 19.7, 17.8 ppm. IR (KBr) υ: 3726, 3623, 2950, 1745, 1674, 1552, 1490, 1292, 1233, 1100, 1033, 966, 843, 757 cm^{-1}; MS (*m*/*z*): HRMS (ESI) Calcd. for $C_{27}H_{27}NaN_2O_6$ ([M+Na]$^+$): 514.1836, found: 514.1831.

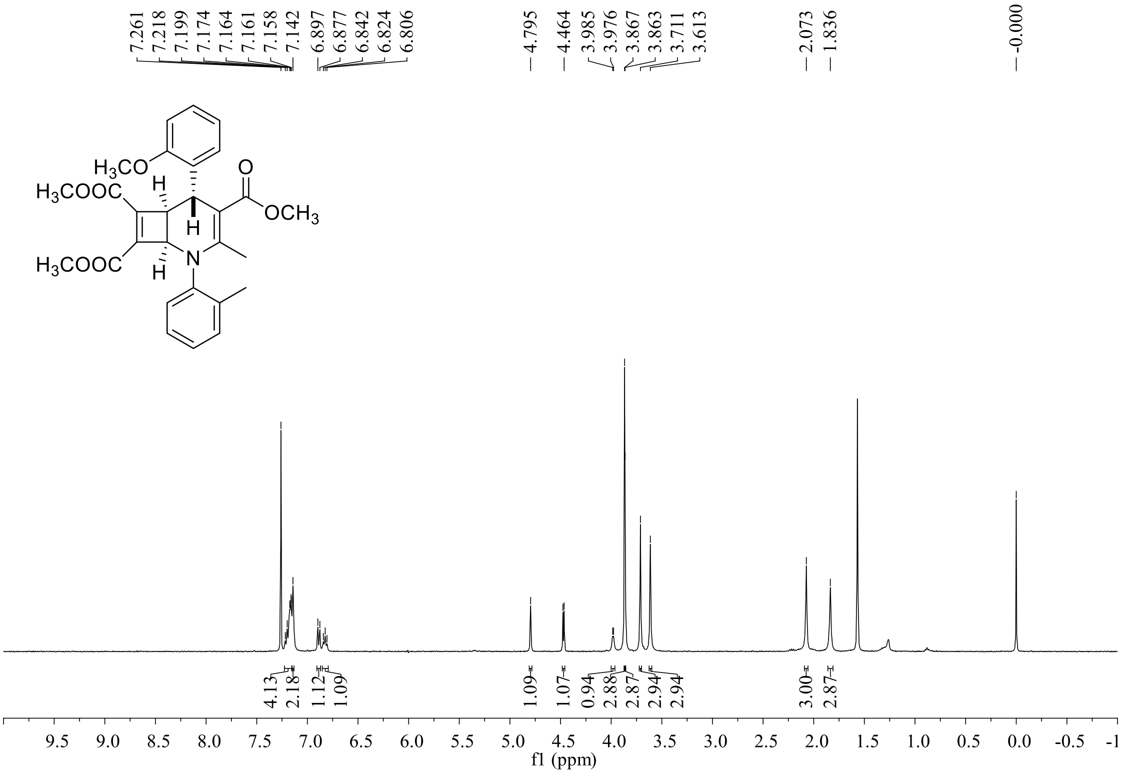

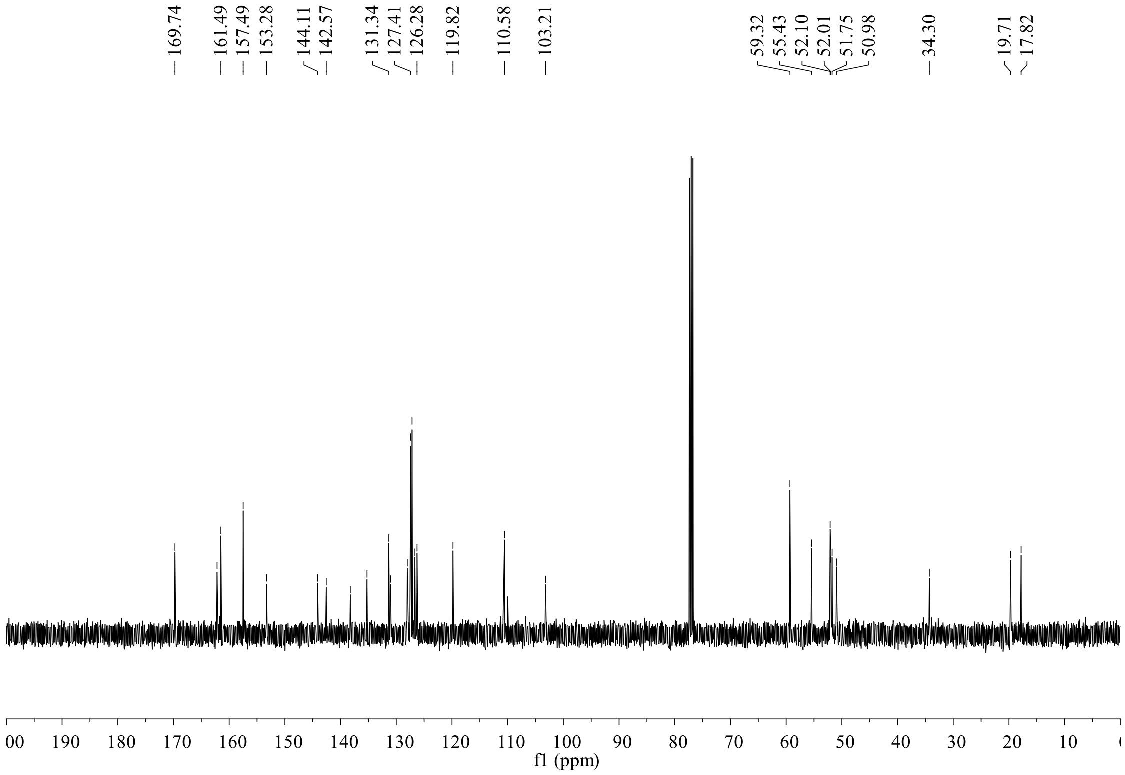
169.74
161.49
157.49
153.28
144.11
142.57
131.34
127.41
126.28
119.82
110.58
103.21
59.32
55.43
52.10
52.01
51.75
50.98
34.30
19.71
17.82
00 190 180 170 160 150 140 130 120 110 100 90 80 70 60 50 40 30 20 10
f1 (ppm)

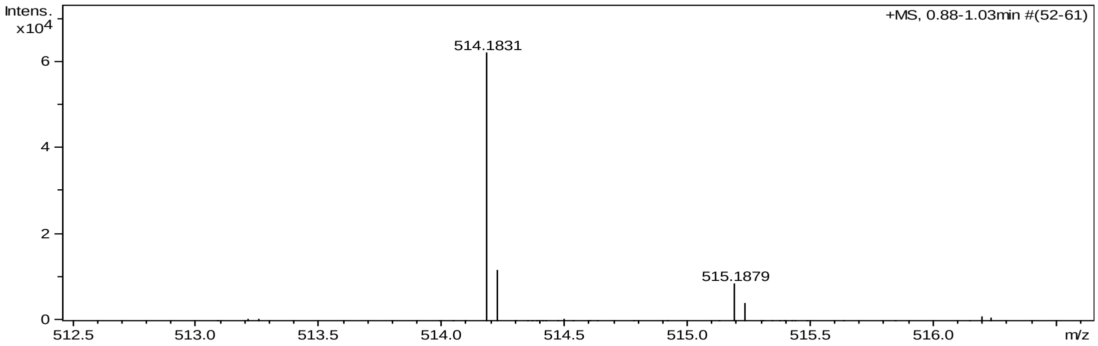
Intens.
x10^4
+MS, 0.88-1.03min #(52-61)
514.1831
515.1879
0
2
4
6
512.5 513.0 513.5 514.0 514.5 515.0 515.5 516.0
m/z

4,5-Diethyl 3-methyl 1-benzyl-2-methyl-3a-(4-nitrophenyl)-1,3a,4,6a-tetrahydrocyclo-penta[*b*]pyrrole-3,4,5-tricarboxylate (6e): yellow solid, 35%, m.p. 153-155 °C; ^{1}H NMR (400 MHz, $CDCl_3$) δ 7.98 (d, *J* = 8.8 Hz, ArH), 7.45-7.37 (m, 5H, ArH), 7.30-7.27 (m, 2H, ArH), 6.70 (s, 1H, CH_2), 5.07-5.05 (m, 1H, CH), 5.00-4.98 (m, 1H, CH), 4.76 (d, *J* = 15.2 Hz, 1H, CH_2), 4.37 (d, *J* = 15.6 Hz, 1H, CH_2), 4.29-4.21 (m, 1H, CH_2), 4.21-4.14 (m, 1H, CH_2), 3.91-3.82 (m, 1H, CH_2), 3.82-3.72 (m, 1H, CH_2), 3.69 (s, 3H, OCH_3), 2.34 (s, 3H, CH_3), 1.29 (t, *J* = 7.2 Hz, CH_3), 0.99 (t, *J* = 7.2 Hz, CH_3) ppm. ^{13}C NMR (100 MHz, $CDCl_3$) δ 172.2, 166.1, 163.5, 158.0, 149.9, 146.3, 137.8, 137.4, 135. 9, 129.2, 128.5, 128.0, 127.2, 123.0, 101.9, 75.5, 62.9, 61.1, 61.0, 58.3, 50.3, 49.2, 14.1, 13.8, 13.4 ppm. IR (KBr) υ: 3069, 2981, 1736, 1660, 1552, 1514, 1344, 1222, 1121, 1040, 914, 854, 769, 704 cm^{-1}; MS (*m*/*z*): HRMS (ESI) Calcd. for $C_{29}H_{30}NaN_2O_8$ ($[M+Na]^+$): 557.1894, found: 557.1891.

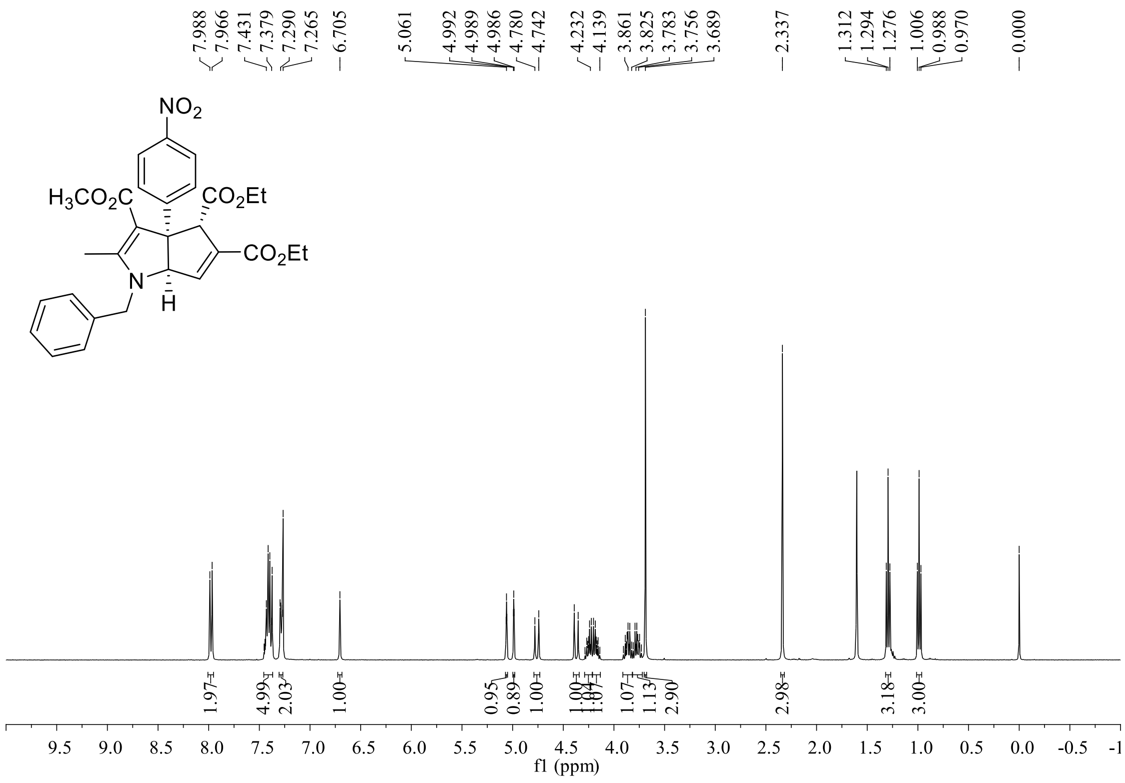

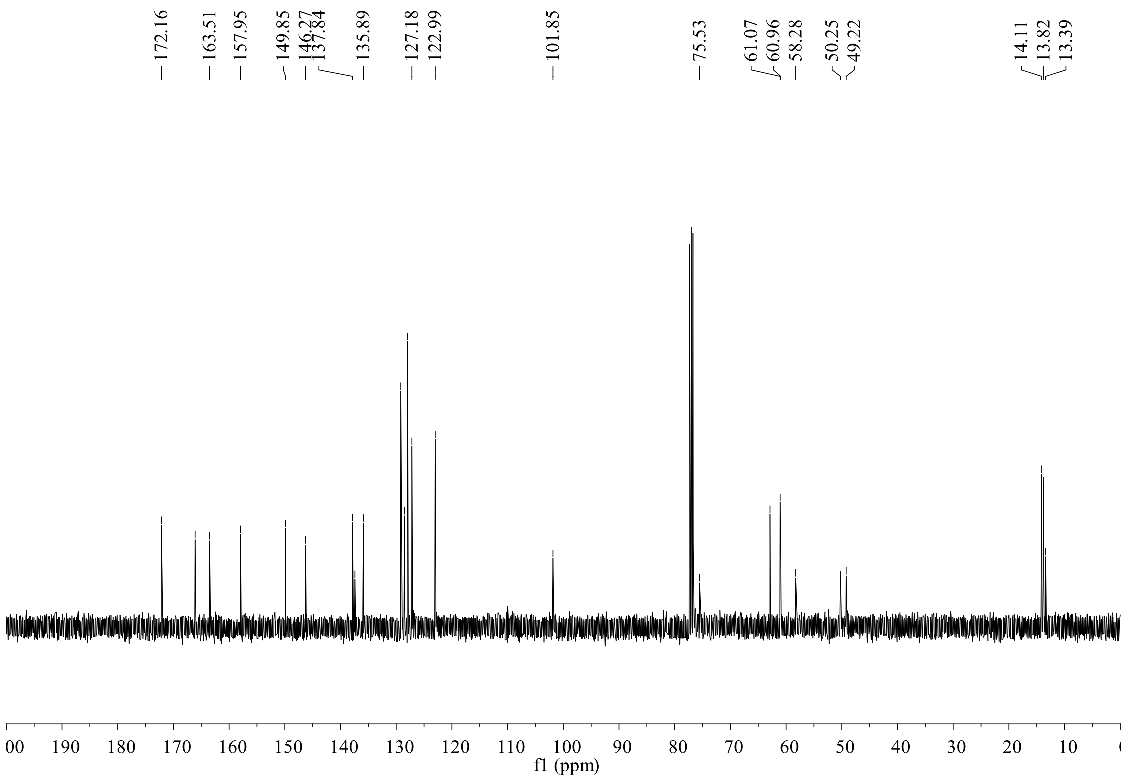
172.16
163.51
157.95
149.85
146.27
137.84
135.89
127.18
122.99
101.85
75.53
61.07
60.96
58.28
50.25
49.22
14.11
13.82
13.39
00 190 180 170 160 150 140 130 120 110 100 90 80 70 60 50 40 30 20 10
f1 (ppm)

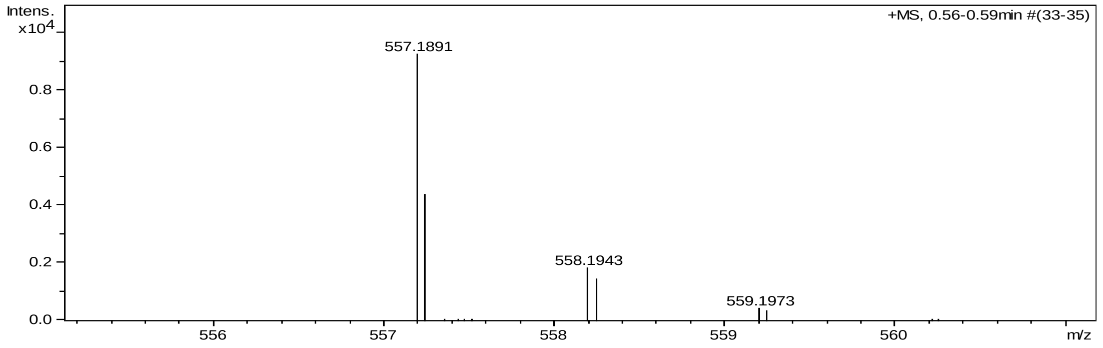
Intens.
x10^4
+MS, 0.56-0.59min #(33-35)
557.1891
558.1943
559.1973
0.8
0.6
0.4
0.2
0.0
556
557
558
559
560
m/z

Trimethyl 1-(4-methoxybenzyl)-2-methyl-3a-phenyl-1,3a,4,6a-tetrahydrocyclo-penta[*b*]pyrrole-3,4,5-tricarboxylate (6f): white solid, 23%, m.p. 146-148 ºC; ^{1}H NMR (400 MHz, $CDCl_3$) δ 7.25-7.21 (m, 2H, ArH), 7.18-7.10 (m, 5H, ArH), 6.88 (d, *J* = 8.4 Hz, 2H, ArH), 6.66 (s, 1H, CH), 5.01 (s, 1H, CH), 4.99 (s, 1H, CH), 4.61 (d, *J* = 15.2 Hz, 1H, CH_2), 4.32 (d, *J* = 15.6 Hz, 1H, CH_2), 3.82 (s, 3H, OCH_3), 3.74 (s, 3H, OCH_3), 3.70 (s, 3H, OCH_3), 3.29 (s, 3H, OCH_3), 2.30 (s, 3H, CH_3) ppm. ^{13}C NMR (100 MHz, $CDCl_3$) δ 172.9, 166.7, 164.3, 159.4, 157.8, 142.7, 138.6, 137.3, 129.0, 128.2, 127.8, 126.4, 126.3, 114.3, 102.7, 76.4, 63.1, 58.6, 55.4, 52.0, 51.7, 50.1, 48.7, 13.3 ppm. IR (KBr) υ: 3726, 3696, 2952, 1716, 1658, 1552, 1513, 1436, 1332, 1237, 1035, 959, 811, 770 cm^{-1}; MS (*m*/*z*): HRMS (ESI) Calcd. for $C_{28}H_{29}NaNO_7$ ($[M+Na]^+$): 514.1836, found: 514.1839.

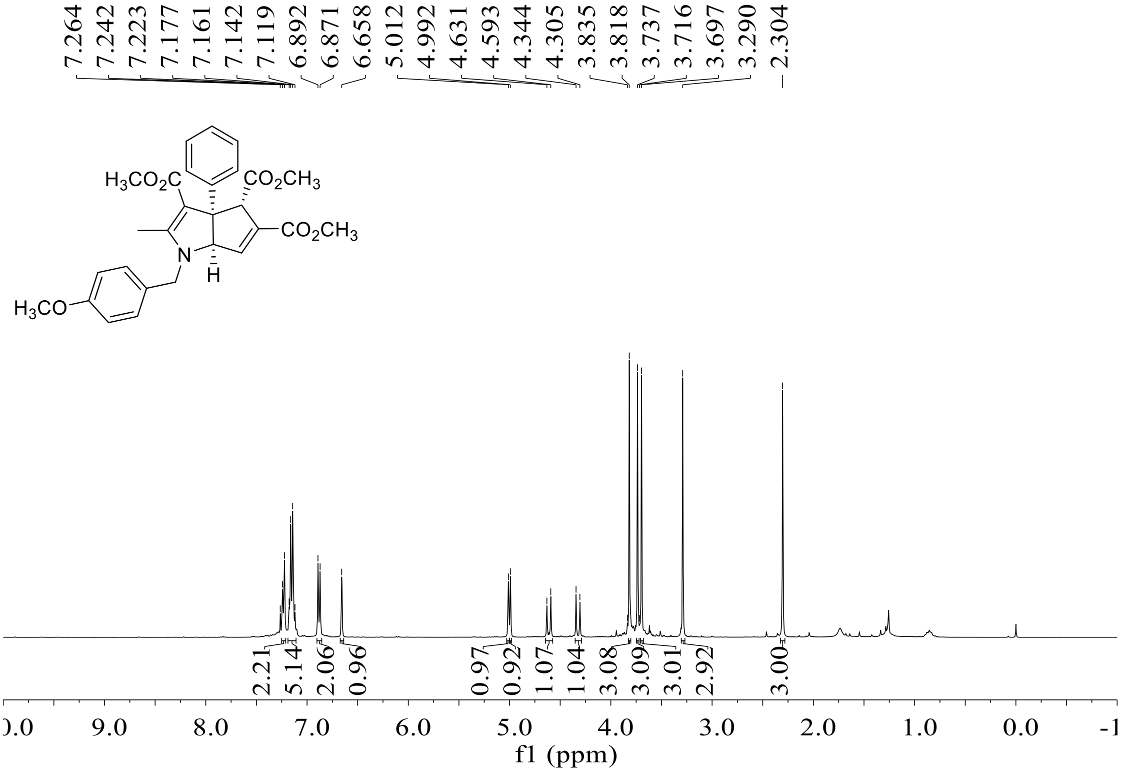

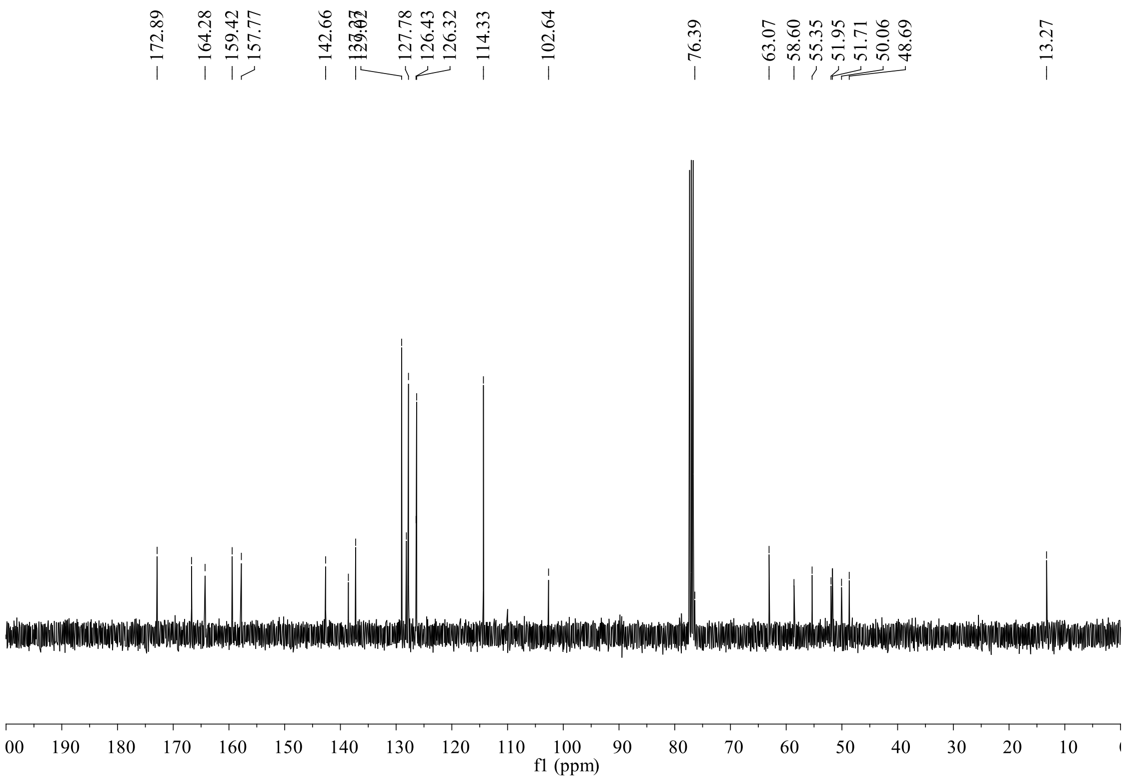
172.89
164.28
159.42
157.77
142.66
129.02
127.78
126.43
126.32
114.33
102.64
76.39
63.07
58.60
55.35
51.95
51.71
50.06
48.69
13.27
00 190 180 170 160 150 140 130 120 110 100 90 80 70 60 50 40 30 20 10
f1 (ppm)

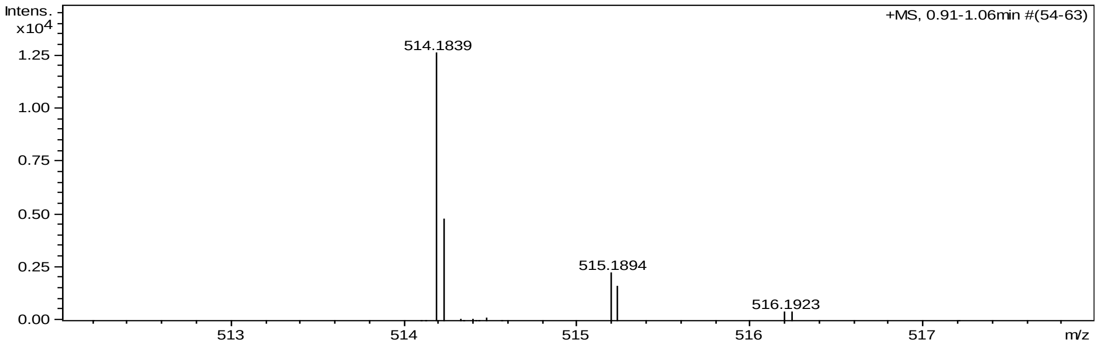
Intens.
x10^4
1.25
1.00
0.75
0.50
0.25
0.00
+MS, 0.91-1.06min #(54-63)
514.1839
515.1894
516.1923
513
514
515
516
517
m/z

4,5-Diethyl 3-methyl 1-(4-methoxybenzyl)-2-methyl-3a-(4-nitrophenyl)-1,3a,4,6a-tetrahydro-cyclopenta[*b*]pyrrole-3,4,5-tricarboxylate (6i): yellow solid, 39%, m.p. 159-161 ºC; ^{1}H NMR (400 MHz, $CDCl_3$) δ 8.01-7.95 (m, 2H, ArH), 7.41-7.37 (m, 2H, ArH), 7.23-7.18 (m, 2H, ArH), 6.96-6.91 (m, 2H, ArH), 6.70-6.67 (m, 1H, CH), 5.04 (s, 1H, CH), 4.99-4.97 (m, 1H, CH), 4.69 (d, J = 15.2 Hz, 1H, CH_2), 4.31 (d, J = 15.2 Hz, 1H, CH_2), 4.26-4.21 (m, 1H, CH_2), 4.21-4.13 (m, 1H, CH_2), 3.91-3.86 (m, 1H, CH_2), 3.85 (s, 3H, OCH_3), 3.81-3.73 (m, 1H, CH_2), 3.68 (s, 3H, OCH_3), 2.33 (s, 3H, CH_3), 1.29 (t, J = 7.2 Hz, 3H, CH_3), 0.99 (t, J = 6.8 Hz, 3H, CH_3) ppm. ^{13}C NMR (100 MHz, $CDCl_3$) δ 172.2, 166.1, 163.5, 159.7, 157.9, 149.9, 146.3, 137.8, 137.5, 129.3, 127.6, 127.2, 123.0, 114.5, 101.8, 75.3, 62.8, 61.1, 61.0, 58.2, 55.4, 50.2, 48.6, 14.1, 13.8, 13.4 ppm. IR (KBr) υ: 3726, 3696, 2985, 1724, 1672, 1561, 1514, 1413, 1347, 1232, 1186, 1028, 851, 769 cm^{-1}; MS (*m*/*z*): HRMS (ESI) Calcd. for $C_{30}H_{32}NaN_2O_9$ ($[M+Na]^+$): 587.2000, found: 587.1995.

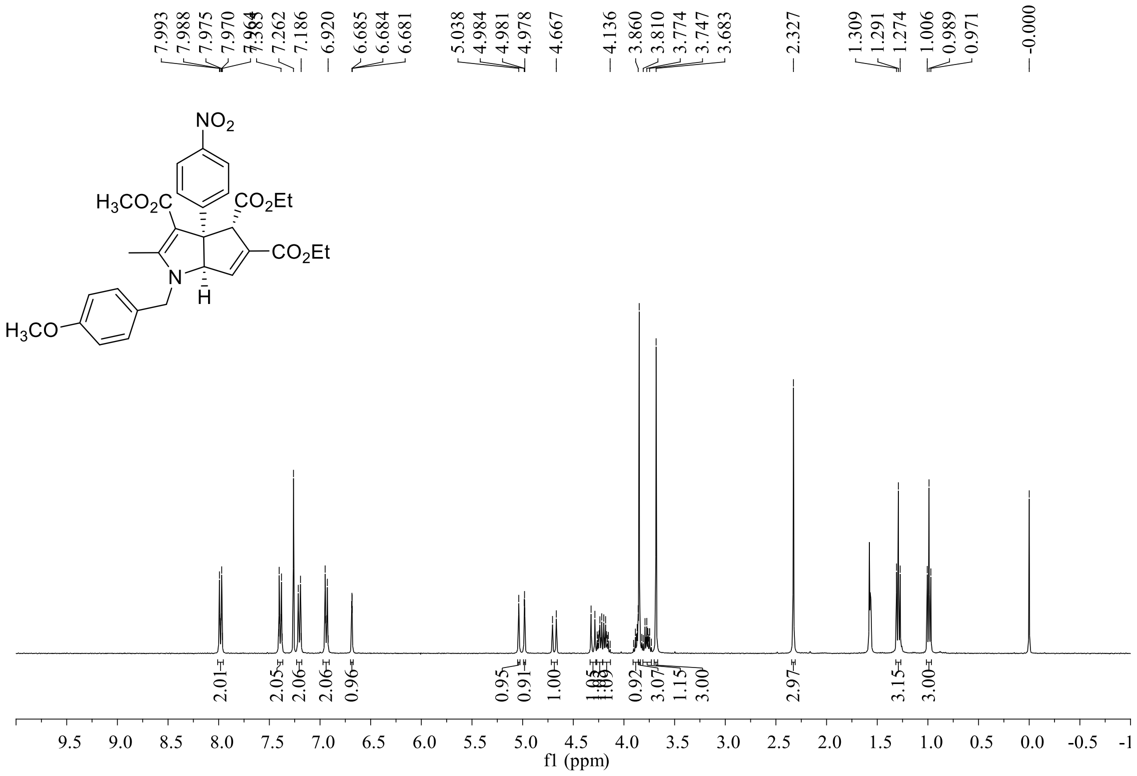

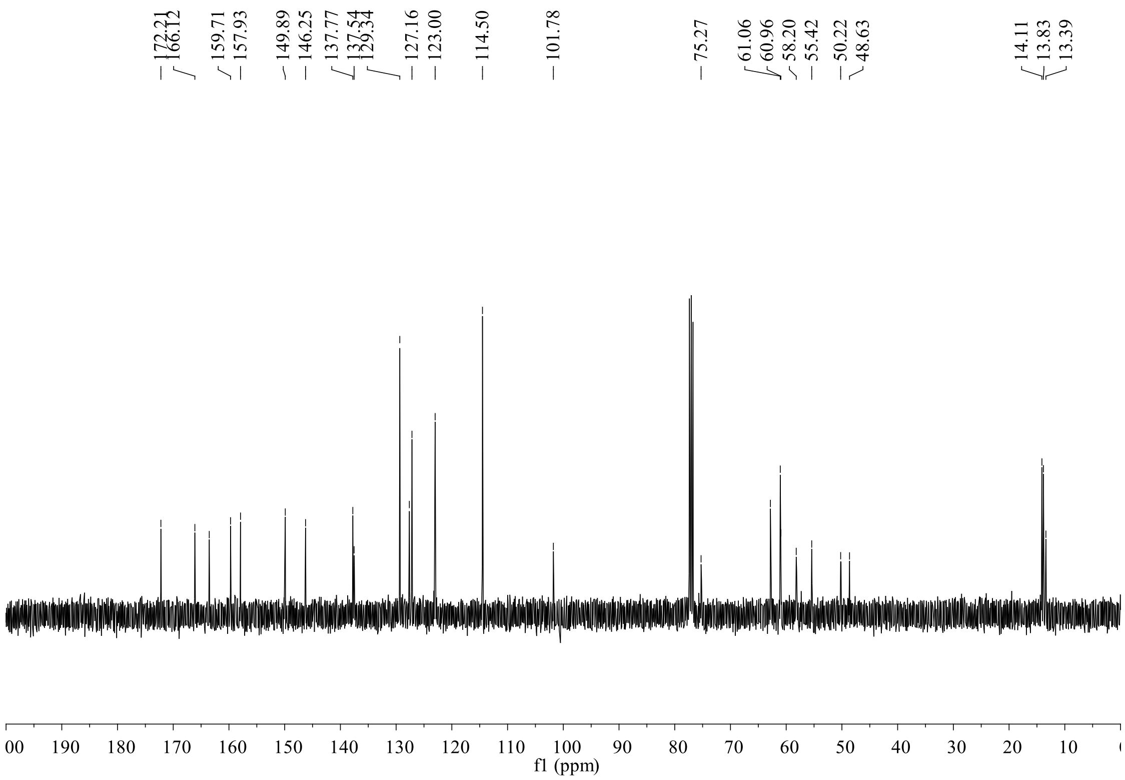
172.21
166.12
159.71
157.93
149.89
146.25
137.77
137.54
129.34
127.16
123.00
114.50
101.78
75.27
61.06
60.96
58.20
55.42
50.22
48.63
14.11
13.83
13.39
00
190
180
170
160
150
140
130
120
110
100
90
80
70
60
50
40
30
20
10
f1 (ppm)

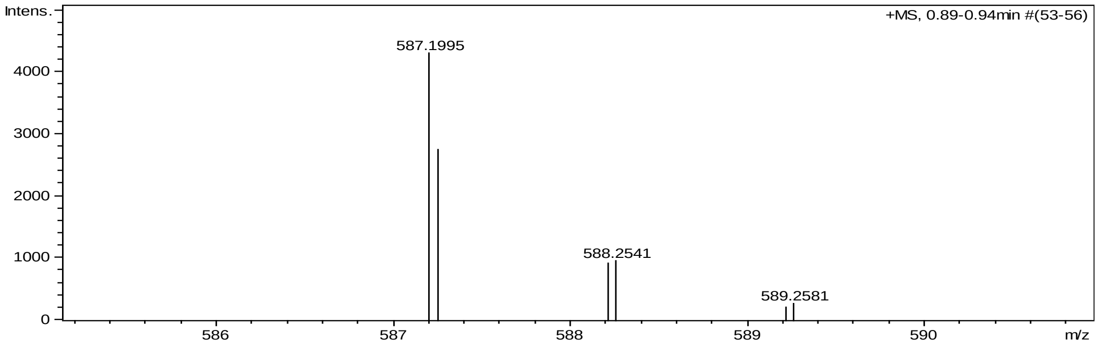
Intens.
+MS, 0.89-0.94min #(53-56)
587.1995
588.2541
589.2581
4000
3000
2000
1000
0
586
587
588
589
590
m/z

Trimethyl 1-(4-methoxyphenyl)-2-methyl-3a-phenyl-1,3a,4,6a-tetrahydrocyclo-penta[*b*]pyrrole-3,4,5-tricarboxylate (6k): white solid, 36%, m.p. 169-171 ºC; ^{1}H NMR (400 MHz, $CDCl_3$) δ 7.47 (d, J = 7.6 Hz, 2H, ArH), 7.30-7.27 (m, 1H, ArH), 7.25-7.24 (m, 1H, ArH), 7.21-7.16 (m, 1H, ArH), 7.10 (d, J = 8.8 Hz, 2H, ArH), 6.95 (d, J = 8.8 Hz, 2H, ArH), 6.76 (s, 1H, CH), 5.46 (s, 1H, CH), 5.06 (s, 1H, CH), 3.85 (s, 3H, OCH_3), 3.76 (s, 3H, OCH_3), 3.72 (s, 3H, OCH_3), 3.34 (s, 3H, OCH_3), 2.09 (s, 3H, CH_3) ppm. ^{13}C NMR (100 MHz, $CDCl_3$) δ 172.9, 166.6, 164.3, 158.3, 156.9, 142.8, 139.3, 137.4, 132.6, 128.0, 127.8, 126.6, 126.2, 114.8, 105.5, 80.5, 63.4, 58.4, 55.5, 52.0, 51.8, 50.2, 14.9 ppm. IR (KBr) υ: 3000, 2950, 1733, 1679, 1563, 1514, 1327, 1246, 1176, 1105, 1033, 944, 844, 772 cm^{-1}; MS (m/z): HRMS (ESI) Calcd. for $C_{27}H_{27}NaNO_7$ ($[M+Na]^+$): 500.1680, found: 500.1672.

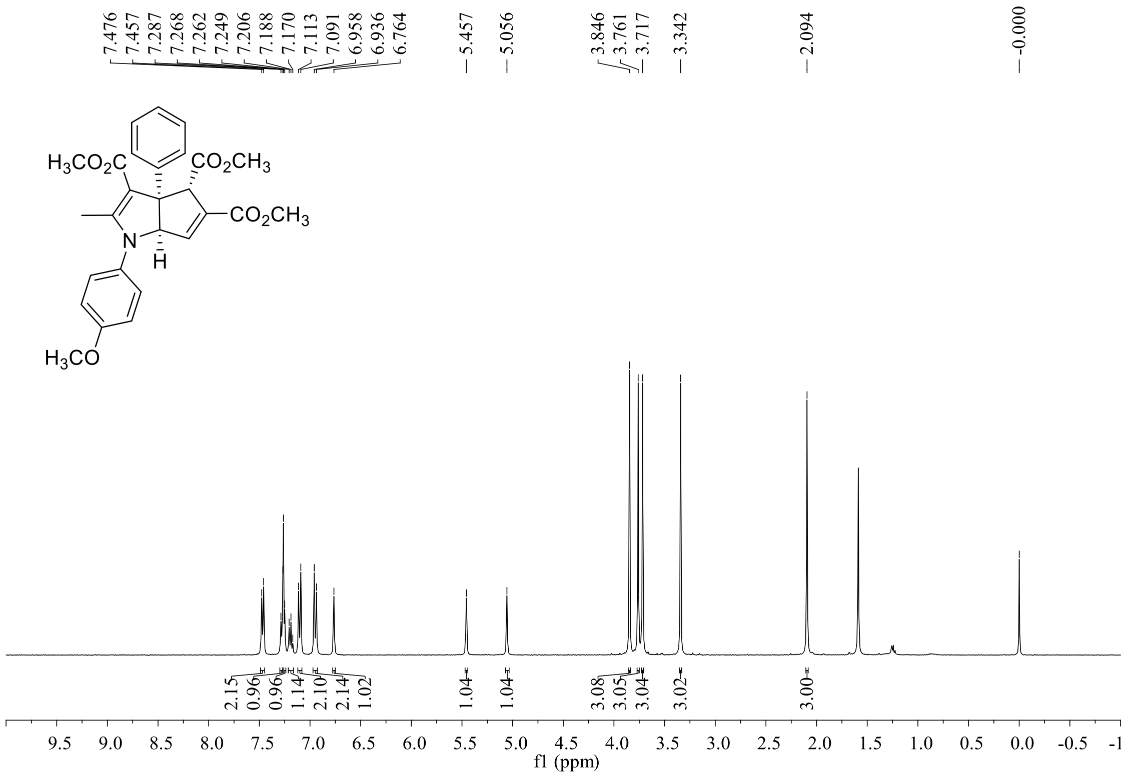

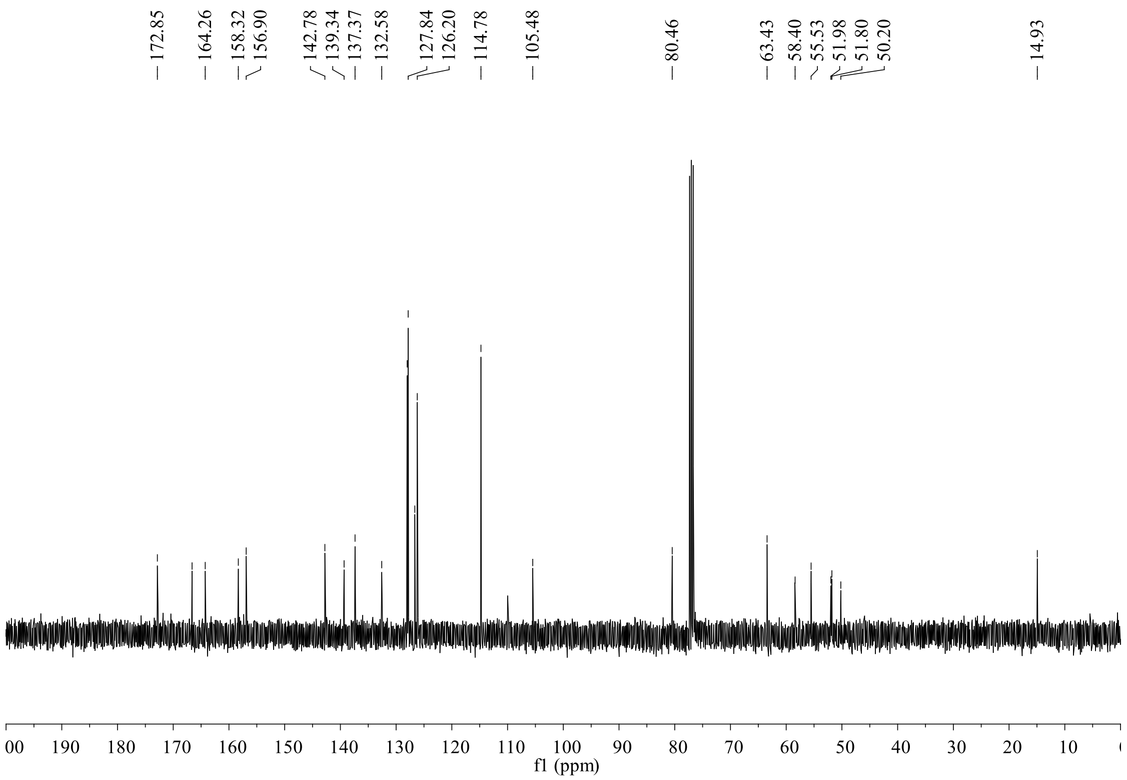
172.85
164.26
158.32
156.90
142.78
139.34
137.37
132.58
127.84
126.20
114.78
105.48
80.46
63.43
58.40
55.53
51.98
51.80
50.20
14.93
f1 (ppm)

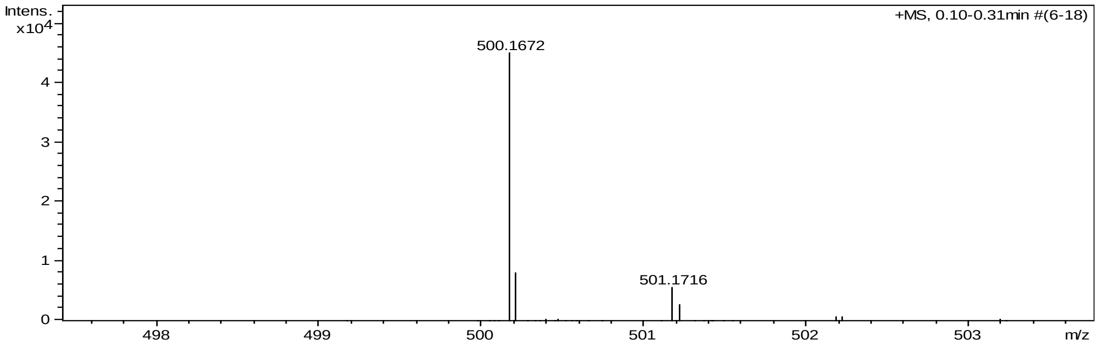
Intens.
x10^4
+MS, 0.10-0.31min #(6-18)
500.1672
501.1716
m/z

4,5-Diethyl 3-methyl 2-methyl-3a-phenyl-1-(p-tolyl)-1,3a,4,6a-tetrahydrocyclo-penta[*b*]pyrrole-3,4,5-tricarboxylate (6l): white solid, 33%, m.p. 164-166 °C; ^{1}H NMR (400 MHz, $CDCl_3$) δ 7.48 (s, 1H, ArH), 7.46 (s, 1H, ArH), 7.24 (s, 2H, ArH), 7.22 (s, 2H, ArH), 7.20-7.15 (m, 1H, ArH), 7.08 (m, 1H, ArH), 7.05 (m, 1H, ArH), 6.79 (s, 1H, CH), 5.50 (s, 1H, CH), 5.03 (s, 1H, CH), 4.28-4.22 (m, 1H, CH_2), 4.21-4.15 (m, 1H, CH_2), 3.90-3.82 (m, 1H, CH_2), 3.79-3.73 (m, 1H, CH_2), 3.71 (s, 3H, OCH_3), 2.39 (s, 3H, CH_3), 2.14 (s, 3H, CH_3), 1.30 (t, J = 7.2 Hz, 3H, CH_3), 0.93 (t, J = 7.2 Hz, 3H, CH_3) ppm. ^{13}C NMR (100 MHz, $CDCl_3$) δ 172.3, 166.6, 163.9, 156.1, 142.7, 139.2, 137.9, 137.4, 136.3, 130.2, 128.0, 126.6, 126.3, 125.5, 106.7, 80.1, 63.6, 61.0, 60.7, 58.2, 50.2, 21.0, 15.2, 14.2, 13.7 ppm. IR (KBr) υ: 2980, 2944, 1715, 1664, 1559, 1516, 1352, 1270, 1228, 1125, 1087, 976, 818, 774 cm^{-1}; MS (m/z): HRMS (ESI) Calcd. for $C_{29}H_{31}NaNO_6$ ($[M+Na]^+$): 512.2004, found: 484. 512.2036.

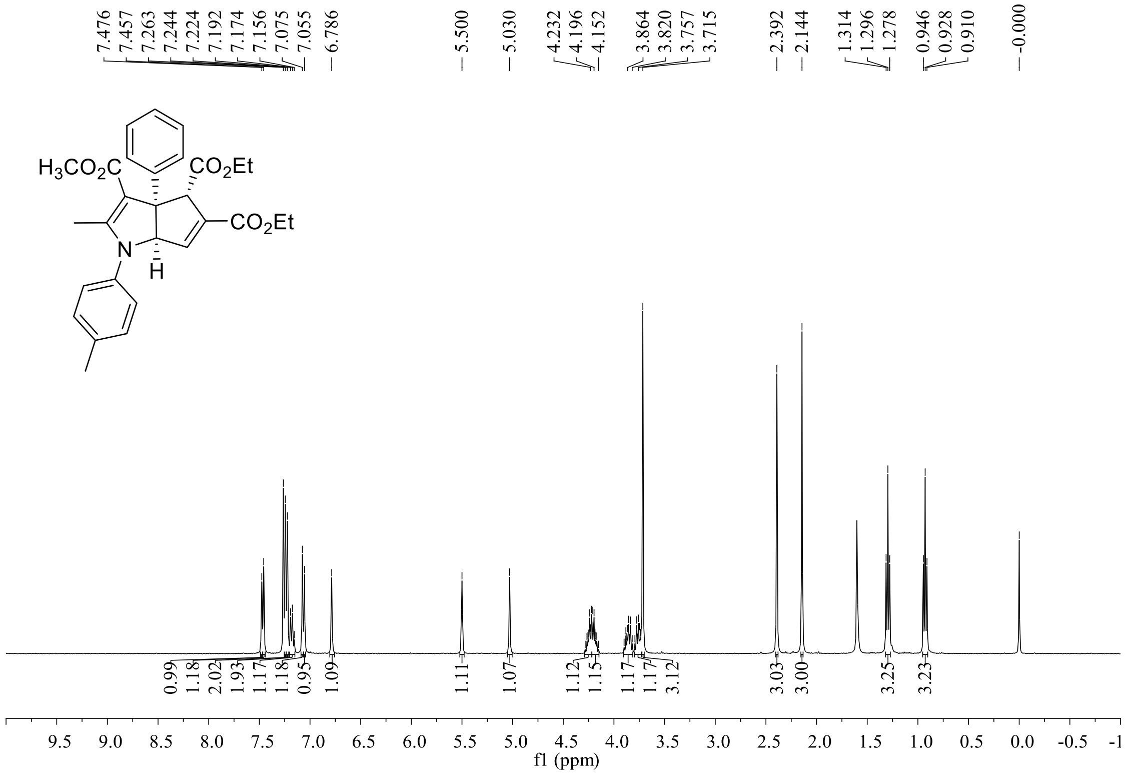

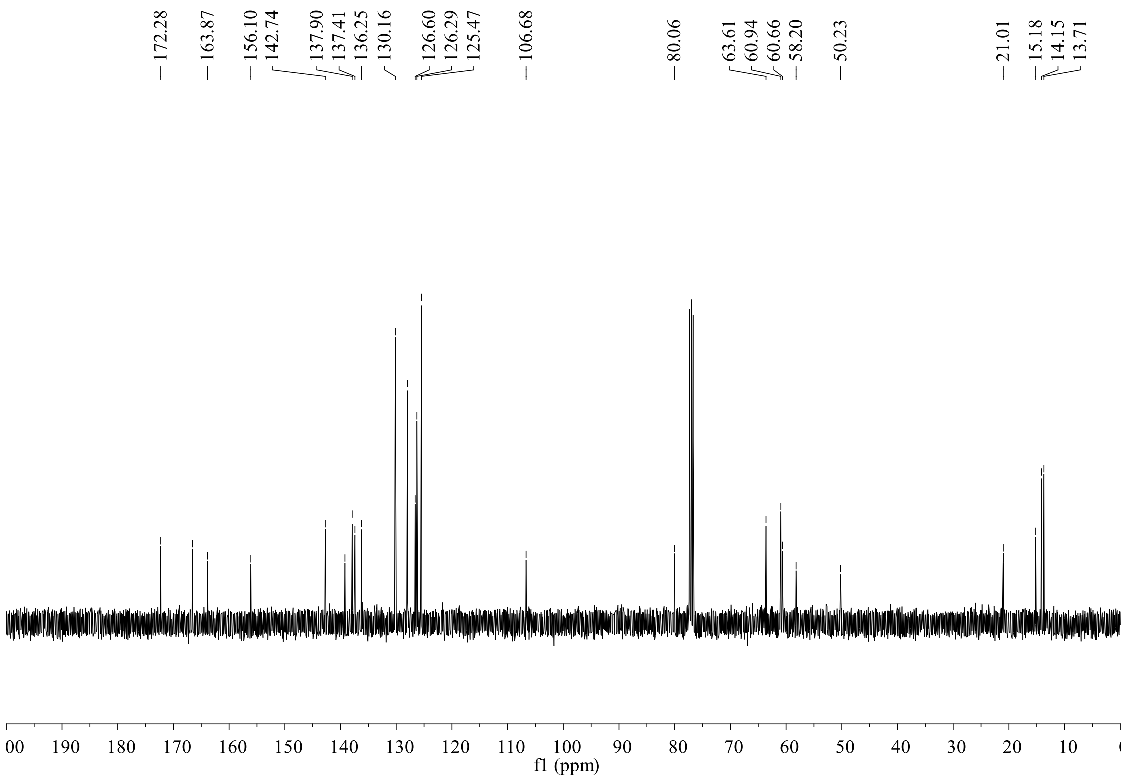

172.28
163.87
156.10
142.74
137.90
137.41
136.25
130.16
126.60
126.29
125.47
106.68
80.06
63.61
60.94
60.66
58.20
50.23
21.01
15.18
14.15
13.71
f1 (ppm)

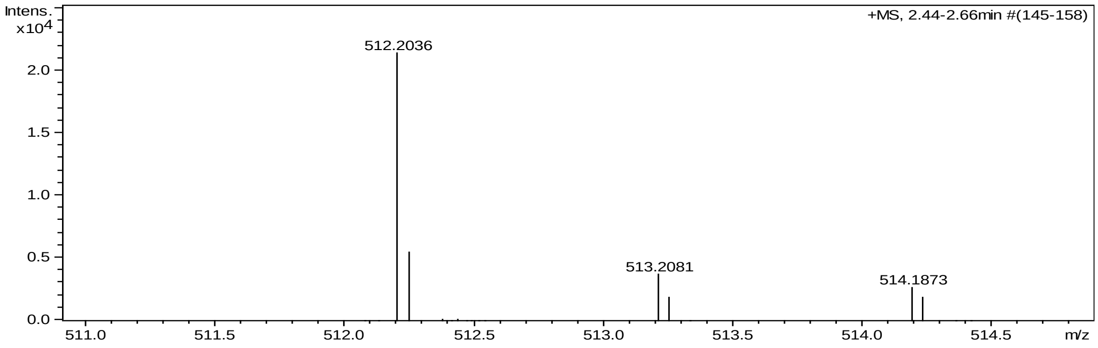

Intens.
x10^4
+MS, 2.44-2.66min #(145-158)
512.2036
513.2081
514.1873
m/z

Trimethyl 1-(4-bromophenyl)-2-methyl-3a-phenyl-1,3a,4,6a-tetrahydrocyclopenta[*b*]pyrrole-3,4,5-tricarboxylate (6m): white solid, 35%, m.p. 206-208 °C; ^{1}H NMR (400 MHz, $CDCl_3$) δ 7.54 (d, *J* = 8.4 Hz, 2H, ArH), 7.38 (d, *J* = 7.2 Hz, 2H, ArH), 7.25-7.18 (m, 3H, ArH), 7.02 (d, *J* = 8.4 Hz, 2H, ArH), 6.79 (s, 1H, CH), 5.51 (s, 1H, CH), 5.07 (s, 1H, CH), 3.77 (s, 3H, OCH_3), 3.73 (s, 3H, OCH_3), 3.36 (s, 3H, OCH_3), 2.17 (s, 3H, CH_3) ppm. ^{13}C NMR (100 MHz, $CDCl_3$) δ 172.7, 166.3, 164.1, 154.9, 142.2, 139.3, 139.2, 137.8, 132.7, 128.2, 126.8, 126.3, 126.0, 119.1, 108.6, 79.9, 63.9, 57.9, 52.1, 51.9, 50.5, 15.3 ppm. IR (KBr) υ: 3726, 2949, 1720, 1668, 1576, 1561, 1494, 1415, 1352, 1230, 1009, 954, 849, 771 cm^{-1}; MS (*m/z*): HRMS (ESI) Calcd. for $C_{26}H_{24}BrNaNO_6$ ([M+Na]$^+$): 548.0679, found: 548.0678.

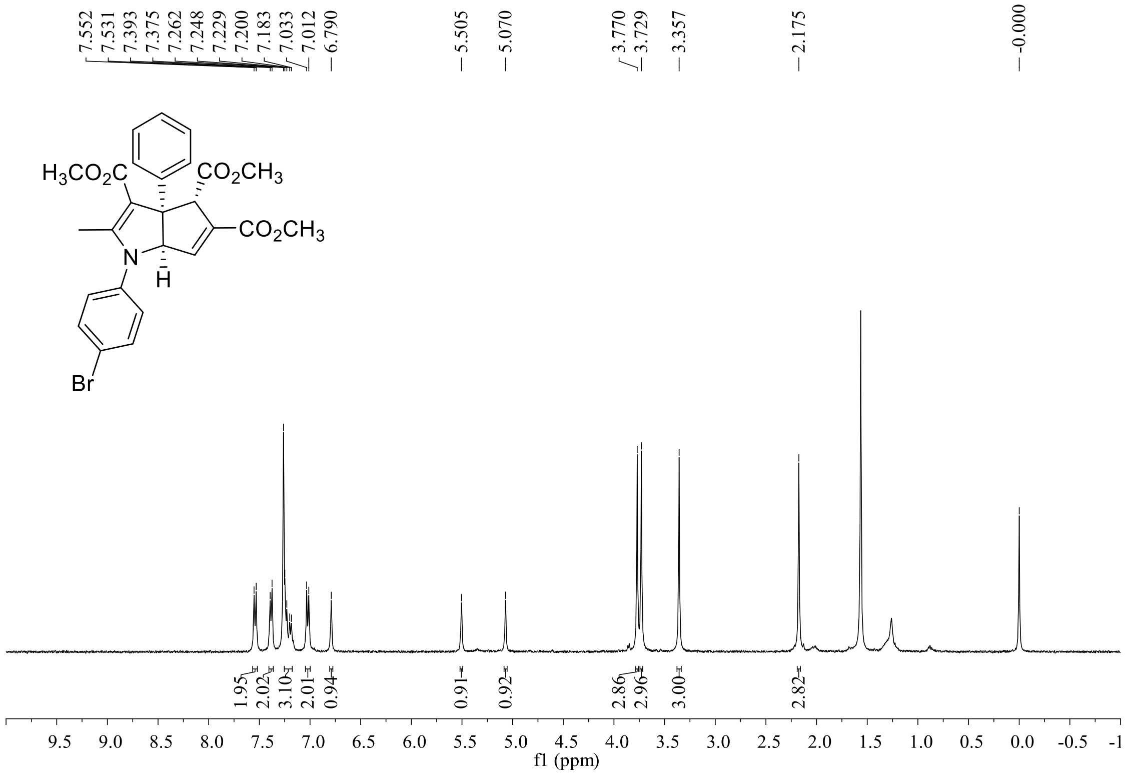

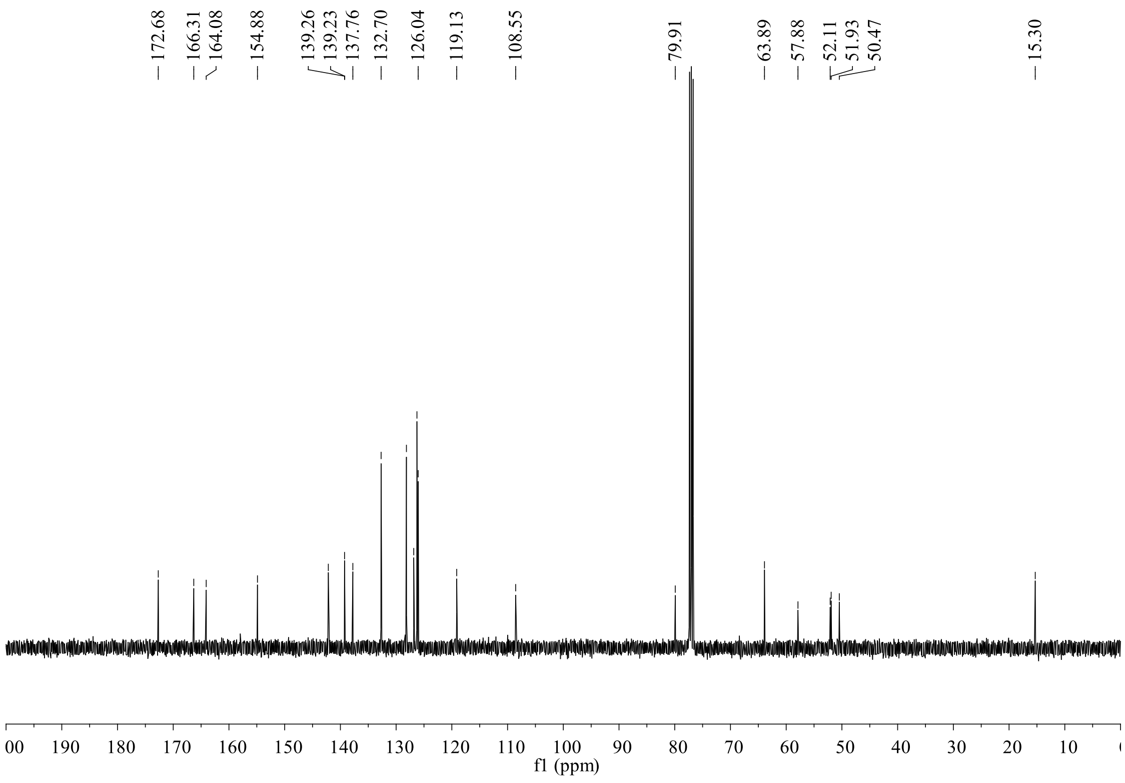
172.68
166.31
164.08
154.88
139.26
139.23
137.76
132.70
126.04
119.13
108.55
79.91
63.89
57.88
52.11
51.93
50.47
15.30
f1 (ppm)

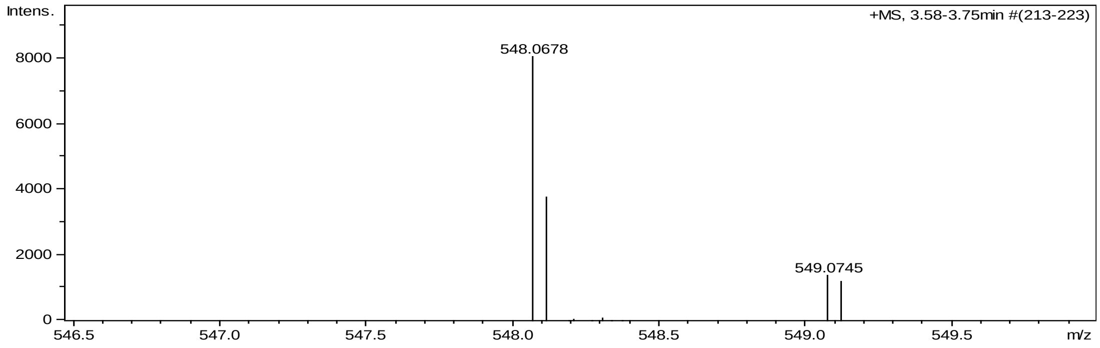
Intens.
+MS, 3.58-3.75min #(213-223)
548.0678
549.0745
m/z
